# Surface and Interfacial Engineering toward Durable Mechanically Coupled Flexible Electronics

**DOI:** 10.1002/advs.76797

**Published:** 2026-08-06

**Authors:** Qian Wang, Fangfang Dai, Wei Wu

**Affiliations:** ^1^ Department of Obstetrics and Gynecology of Renmin Hospital, School of Physics and Technology Wuhan University Wuhan P. R. China; ^2^ School of Future Technology Wuhan Textile University Wuhan P. R. China

**Keywords:** device reliability, flexible electronics, interface, mechanical sensing, surface

## Abstract

Mechanically coupled flexible electronics enable real‐time, conformal monitoring of diverse mechanical signals. However, their operation in dynamic and complex environments places stringent demands on surface and interfacial reliability. Insufficient interfacial functionalization, unstable contact, as well as mechanical and electrical mismatch have emerged as key factors limiting device durability, signal fidelity, and practical deployment. This review summarizes the fundamental surface and interfacial challenges in mechanically coupled flexible electronics from the perspective of surface and interfacial physics, including contact instability, interfacial failure, stress concentration, and environmental degradation. To address these challenges, various material systems designed to enhance surface and interfacial performance are systematically reviewed, focusing on surface chemical modification, interfacial chemical engineering, as well as processing‐ and manufacturing‐enabled physical reinforcement approaches. In addition to isolated chemical or physical methods, multiscale surface and interface architectures are further engineered through bioinspired interfaces, mechanical interlocking, adhesive interlayers, buffering layers, percolating interpenetrating networks, and homogeneous structural design. The resulting improvements in reliability and functional stability are illustrated through representative applications across multiple operating scenarios. Finally, current challenges and future directions are outlined in terms of standardization, environmental robustness, manufacturing repeatability, and large‐scale implementation.

## Graphical abstract

This Review highlights how surface and interfacial engineering governs the reliability, durability, and functional stability of mechanically coupled flexible electronics. By integrating fundamental interfacial theory, failure mechanisms, multiscale engineering strategies, and representative applications, it provides a design‐oriented framework for overcoming contact instability, interfacial degradation, and performance loss in practical flexible electronic systems.

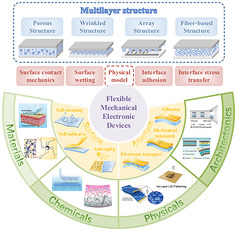

## 1 Introduction

Flexible electronics have emerged as a transformative platform for next‐generation wearable healthcare [1, 2, 3, 4], human‐machine interfaces [5, 6], intelligent robotics, the recognition and manipulation of irregularly shaped objects, and structural health monitoring in industrial systems [7, 8, 9, 10, 11]. These advantages are attributed to their lightweight nature, conformability, bendability, and ability to integrate with complex and dynamic surfaces [12, 13, 14]. Compared with conventional silicon‐based electronics, they can maintain functional coupling with soft biological tissues, deformable structures, and irregular engineering objects under mechanical disturbance. Within this broad field, mechanically coupled flexible electronics constitute a particularly important category because their operating and service conditions are intrinsically associated with pressure, strain, shear, vibration, or frictional contact. Accordingly, their performance is influenced by how external surfaces establish adaptive contact with target objects and how internal interfaces maintain structural integrity and functional continuity in multilayer architectures.

Reliable operation is therefore governed by both external surface behavior and internal interlayer stability. On the external side, device surfaces govern conformal adhesion, wettability, antifouling behavior, environmental exposure, and contact stability with biological tissues, textiles, elastomers, metals, or other target substrates. On the internal side, interfaces among substrates, electrodes, active layers, adhesive interlayers, and encapsulation layers determine adhesion, stress transfer, modulus mismatch accommodation [15, 16], crack propagation, delamination, and interlayer separation [17, 18], as well as interfacial ion migration and electrical transport continuity [19, 20] during cyclic deformation. Therefore, for mechanically coupled flexible electronics, achieving durable function is not merely a matter of improving material performance, but of coordinating surface behavior, interfacial stability, and structural design throughout the entire device architecture.

**FIGURE 1:**
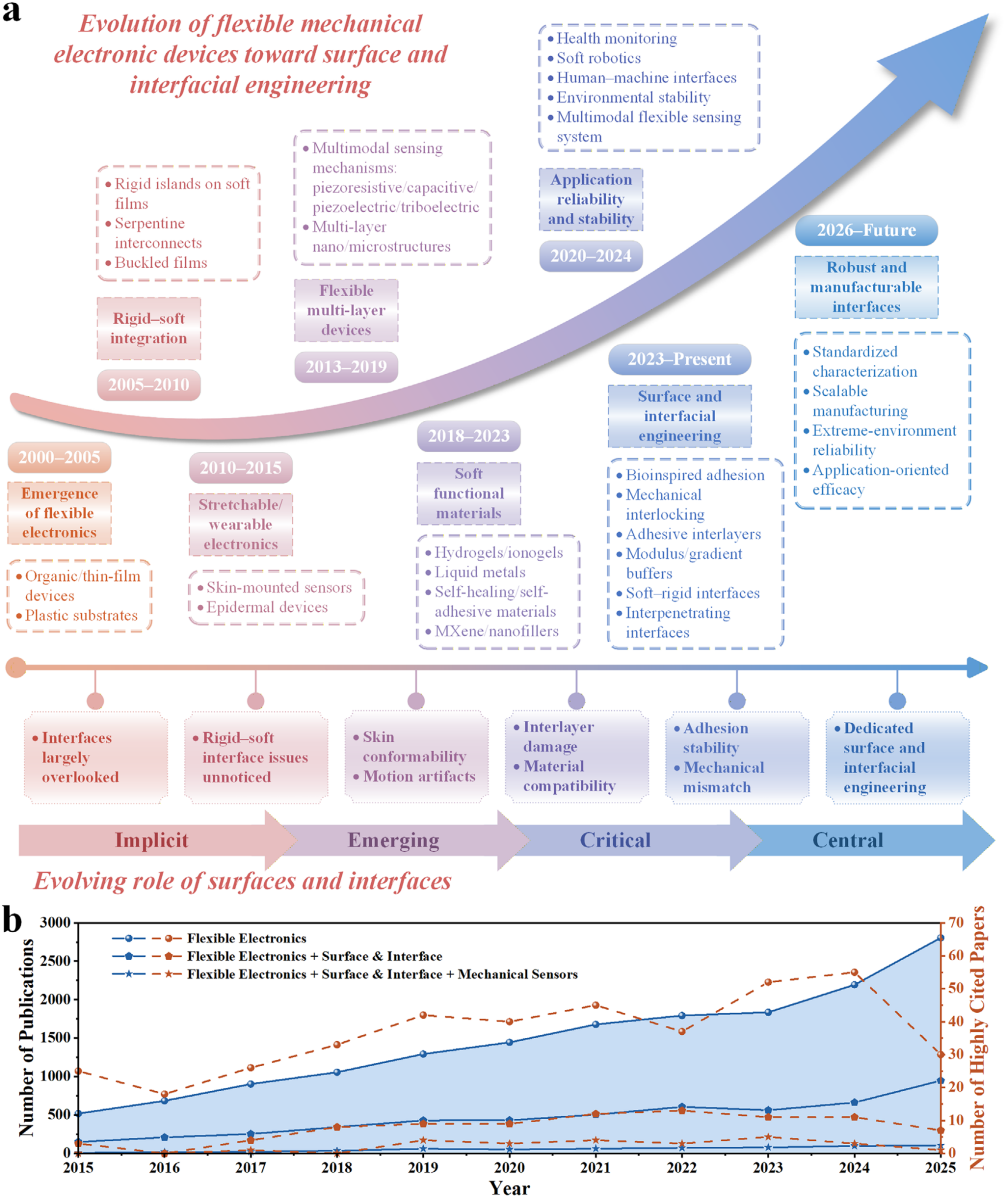
Research evolution and growing attention to surface/interfacial issues in mechanically coupled flexible electronics. (a) Schematic timeline of representative developments in this field. (b) Publication trends and highly cited papers related to flexible electronics, surface and interface studies, and mechanical sensing devices from 2015 to 2025, retrieved from the Web of Science database.

The importance of this topic has become increasingly evident over the past decade. As shown in Figure 1a, flexible electronics has evolved from early mechanically compliant materials and relatively simple device configurations into increasingly sophisticated systems with greater structural complexity, multifunctionality, and application adaptability [21, 22]. In parallel, surfaces and interfaces have emerged as increasingly critical design determinants. Beyond the pursuit of higher performance metrics and more elaborate device architectures, growing attention has been directed to the surface and interfacial factors that ultimately govern long‐term durability, functional fidelity, and practical deployability under realistic operating conditions. This trend is further reflected in Figure 1b, which shows the sustained growth in publications and highly cited papers related to flexible electronics, surface/interface studies, and mechanically responsive devices in recent years. Taken together, these developments suggest that contact and interface reliability are no longer secondary considerations in flexible device design, but central to achieving durable operation and practical deployment.

**FIGURE 2:**
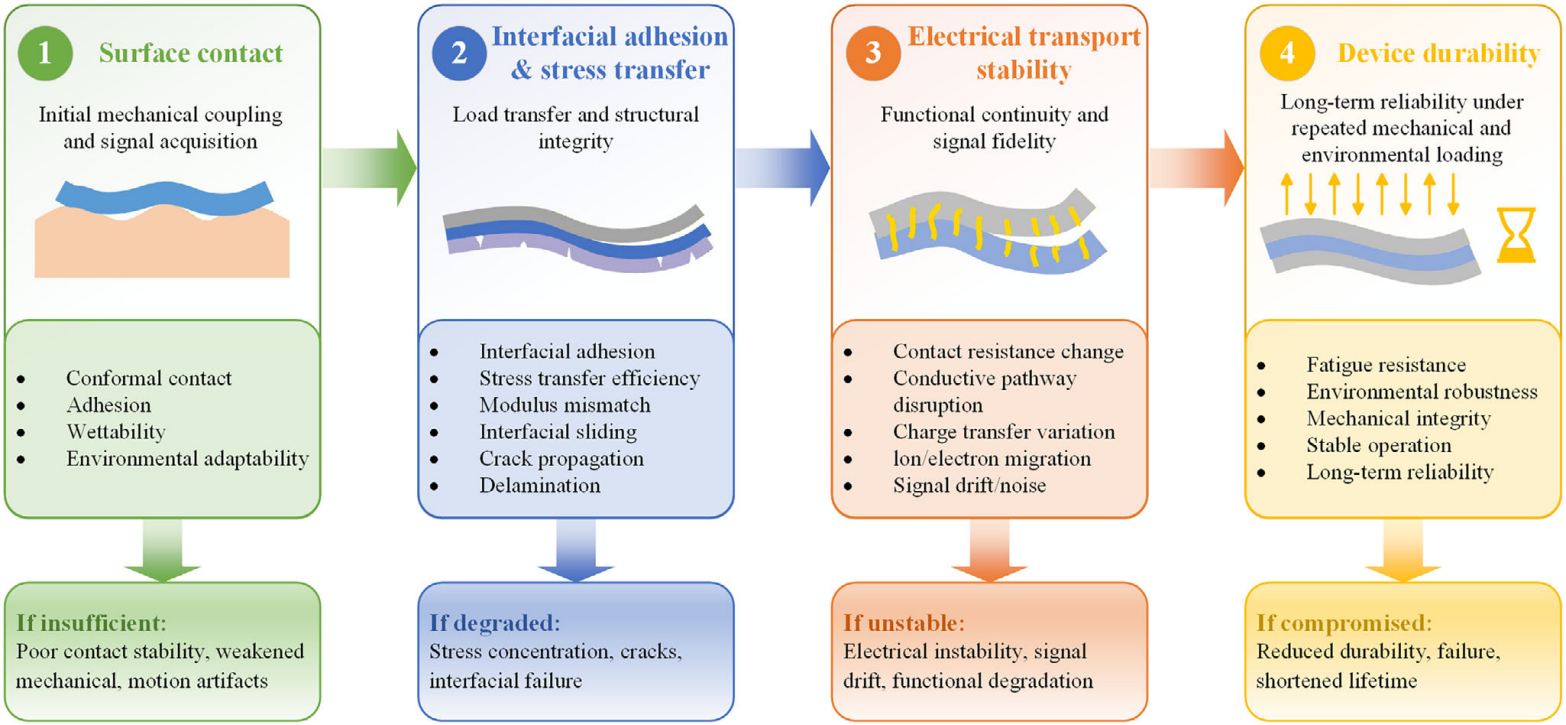
Reliability evolution of mechanically coupled flexible electronics governed by the coupled effects of surface contact, interfacial adhesion/stress transfer, electrical transport stability, and device durability.

In recent years, research on mechanically coupled flexible electronics has mainly focused on material innovation, sensing mechanisms, structural design, fabrication strategies, and application‐specific performance. These efforts have greatly improved sensitivity, deformability, multifunctionality, and system integration. However, contact behavior and interlayer stability are still often discussed only in relation to specific materials or isolated failure phenomena. Existing reviews have provided valuable summaries of surface wettability, interfacial adhesion, binding‐strength enhancement, and application‐oriented flexible devices. However, a unified perspective is still needed to explain how these issues are connected during device operation and failure. In mechanically coupled flexible electronics, the external surface determines whether mechanical stimuli can be stably captured from the target object. Internal interfaces determine whether the captured load can be transferred without severe stress concentration, interfacial sliding, cracking, or delamination. The evolution of these interfaces further determines whether conductive pathways, contact resistance, charge transfer, and signal output remain stable during repeated deformation. From this perspective, device durability should be traced back to the continuous process by which mechanical signals enter, transfer, and remain electrically readable within the device. This process is schematically illustrated in Figure 2.

**FIGURE 3:**
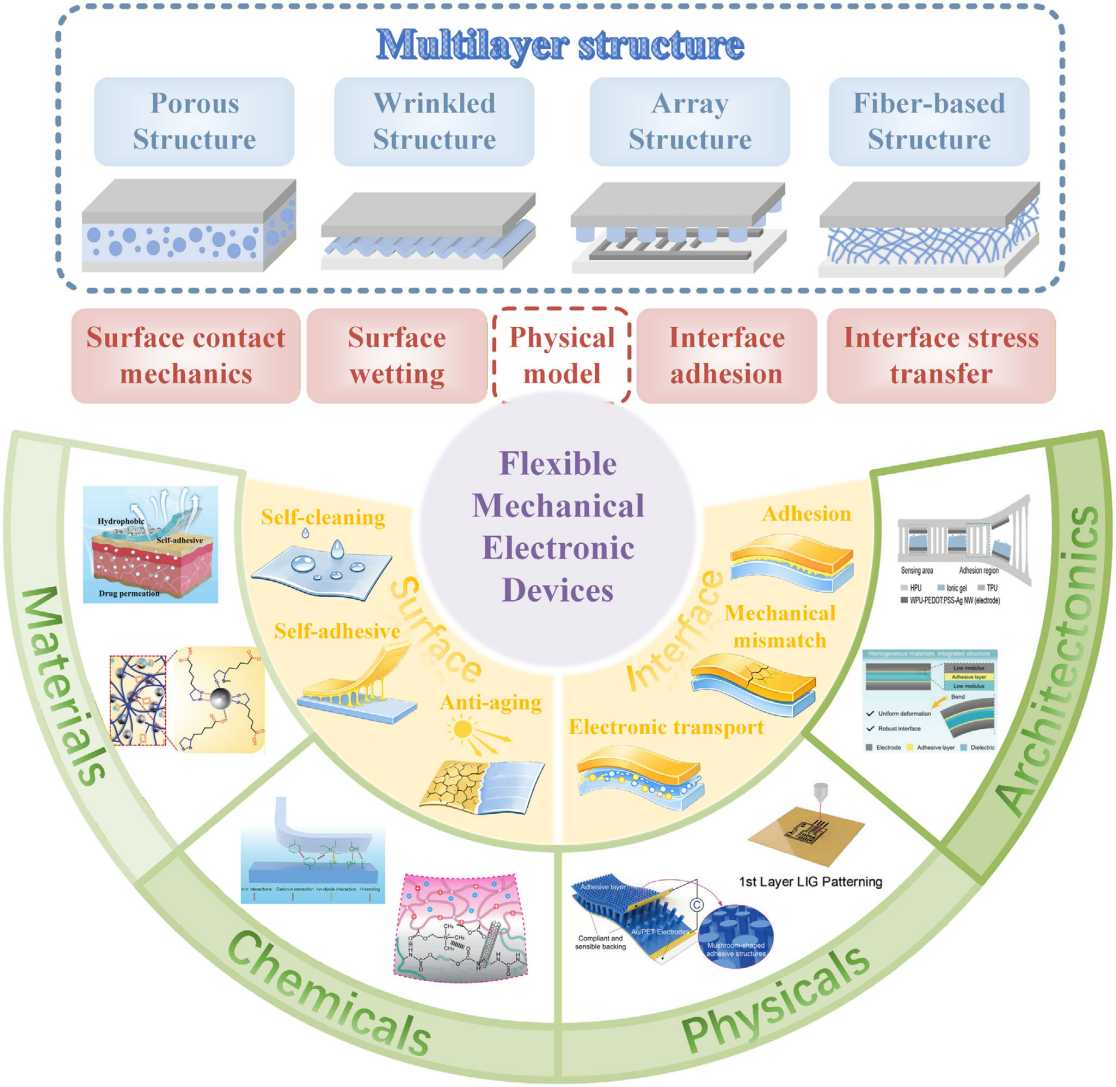
Schematic overview of major surface/interfacial challenges and corresponding enhancement strategies in mechanically coupled flexible electronics [23, 24, 25, 26, 27, 28, 29, 30]. Reproduced under the terms of the Creative Commons Attribution 4.0 International License [23]. Reproduced with permission [24, 25, 26, 27, 28, 29, 30]. Copyright 2025, Wiley‐VCH. Copyright 2022, Wiley‐VCH. Copyright 2023, Elsevier Ltd. Copyright 2025, Elsevier Ltd. Copyright 2024, Wiley‐VCH.

On this basis, this review provides an overview of the major theoretical principles, surface and interfacial challenges, engineering strategies, and representative applications of surface/interfacial regulation in mechanically coupled flexible electronics, with particular emphasis on flexible mechanical sensors (Figure 3). It first outlines the fundamental principles governing surface and interfacial behavior, including contact mechanics, adhesion theory, stress‐based physical models, and typical failure modes of heterogeneous interfaces. It then discusses the major surface and interfacial issues encountered during the fabrication and operation of flexible mechanical devices, together with finite element analysis (FEA) and characterization‐assisted approaches for evaluating interfacial stress distribution, deformation behavior, and failure evolution. Representative strategies for addressing these issues are subsequently summarized from the perspectives of materials design, chemical modification, physical processing, and multiscale interfacial engineering. The resulting improvements in device reliability, functional stability, and operational performance are further discussed, along with representative applications enabled by surface functionalization and well‐regulated interfacial control. Finally, current challenges and future directions in surface and interfacial design are highlighted, including the development of more sustainable and durable engineering strategies for flexible electronic systems. Such a perspective may provide useful guidance for the development of reliable flexible electronics in healthcare monitoring, soft robotics, aerospace, civil infrastructure, and intelligent mechanical systems.

## 2 Fundamental Theories of Surfaces and Interfacial Behaviors in Mechanically Coupled Flexible Electronics

Currently, researchers often overlook the fact that homogeneous properties of materials, multi‐layer interface stability, surface functionalization, and enhanced roughness are key factors in addressing device stability and driving their application. From a theoretical perspective, exploring the mechanical relationships of 2D interfacial layers, physical models of surface interactions with the measured object, and heterogeneous interfacial theory forms the theoretical foundation for the surface and interfacial issues discussed in this review.

### 2.1 Surface Contact, Adhesion, and Wetting Theories

The surface functionalization and performance improvement of flexible electronic devices are mainly achieved from three perspectives: the design of surface morphology and structure changes the surface roughness or patterning to further change the contact area with the measured object; surface chemical modification alters the surface tension and hydrophilic, hydrophobic, oleophilic and other abilities of the device; changes in the physical properties of the film itself further alter the thermodynamic properties of the material, such as thermal conductivity.

#### 2.1.1 Surface Morphology‐Regulated Contact Mechanics

**FIGURE 4:**
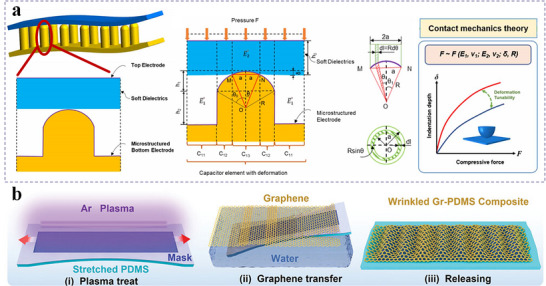
Surface morphology and contact theory of thin films. (a) Analysis of contact behavior between microstructure and planar dielectric layer of flexible capacitive devices with compressive deformation. Adapted with permission [34]. Copyright 2022, IOP Publishing Ltd. (b) The manufacturing process of wrinkling structure. Reproduced with permission [42]. Copyright 2022, Wiley‐VCH.

The design of surface morphology can accurately adjust the contact area and stress distribution between flexible devices and the measured object. By adjusting the surface roughness or introducing micro‐nano patterned structures such as pyramids, cylinders, cones, grooves, wrinkles, and mushroom‐shaped structures, the interaction between the surface of flexible electronic devices and the contact object can be changed, especially significantly affecting the contact force characteristics and surface adhesion strength [24, 31, 32]. The Hertz model is the fundamental mathematical model of contact mechanics, and the normal force on the contact surface can be described by the following Hertz contact Equation (1):

(1) $$P=\frac{4}{3}E^{*}\sqrt{R\delta^{3/2}}$$

where *P* is the contact force, *E* ^*^ is Young's modulus of the composites, *R* is the effective radius of the contact object, and *δ* is the compressive displacement between objects [33]. The Hertz model is most suitable for non‐adhesive, frictionless, elastic contact between smooth bodies under small deformation, such as the first‐order estimation of local contact force between a microstructured elastomer surface and a rigid indenter. Altering the surface micro/nanostructure of a device changes the size and shape of the contact area with the measured object, thereby optimizing the contact force and friction performance. Particularly, the surface micro/nanostructure design can endow the device with surface functionalities such as superhydrophobicity, superadhesion, and self‐cleaning, enabling adaptation to the surfaces of skin, soft robots, and measured components, as shown in Figure 4a [34]. However, the Hertz contact theory is more suitable for rigid physical models with rough surfaces, and many key physical quantities have not been considered, marking the beginning of the development of adhesion force calculations [35]. The adhesion theory models, including the Greenwood model [36], Johnson‐Kendall‐Roberts (JKR) model [37, 38, 39], Derjaguin‐Muller‐Toporov (DMT) model [37, 38, 39], Maugis‐Dugdale (MD) model [38, 40], and Makushkin model [41], are commonly used to explain the contact behavior between surfaces. The Greenwood model introduces the effect of surface roughness. Surface roughness can significantly improve adhesion strength, reduce contact stress concentration, and slow down crack propagation [36]. Nonetheless, excessive roughness can lead to stress concentration. Therefore, optimizing surface morphology design is an important condition for balancing surface contact mechanical properties. The design of hierarchical structures and gradient structures is the development trend for solving this theoretical problem. The JKR model is more applicable to soft, compliant, and high‐surface‐energy materials. The DMT model is suitable for stiff and weakly adhesive interfaces, and Maugis‐Dugdale for the transition regime. For wet, contaminated, or dynamically loaded interfaces, these models should be combined with capillary adhesion, viscoelastic dissipation, or cyclic fatigue analysis.

In addition, dynamic surface morphology (such as reversible wrinkles) allows flexible electronic devices to adjust their contact state under external strain and achieve more stable contact behavior, providing new ideas for the stability of flexible mechanical sensors under dynamic loads (Figure 4b) [42]. Under external stress, reversible structures undergo periodic folding or unfolding on their surfaces, thereby altering the surface contact force during sensor deformation and reducing energy loss. Different thickness ratios and modulus ratios can lead to the occurrence of multiple wrinkled paths, such as wrinkling‐local ridge, wrinkling‐sinusoidal period, and wrinkling‐period doubling [43]. These buckling and wrinkling models are especially useful for thin‐film/substrate systems under compressive strain, where the film is much thinner and stiffer than the substrate. These buckling paths not only change the contact state of the surface, but also achieve a more stable surface morphology through surface sliding and folding behavior, providing a theoretical basis for the design of flexible mechanical sensors.

The synergistic effect of film thickness and surface morphology is also an important factor affecting the contact mechanics of thin film surfaces [44]. Thin films are highly sensitive to surface roughness and contact time. As the film thickness increases, the sensitivity to surface roughness and contact time decreases. The synergistic effect of thin films and moderate film thickness with submicron surface roughness can enhance surface adhesion, while an increase in roughness leads to a decrease in surface adhesion. The critical roughness is an important parameter that determines surface behavior [45]. The relationship between soft elastomers and the critical roughness parameters of films provides an important theoretical foundation for the design of novel flexible electronic adhesive surfaces.

#### 2.1.2 Surface Chemical Modification, Surface Energy, and Wettability

**FIGURE 5:**
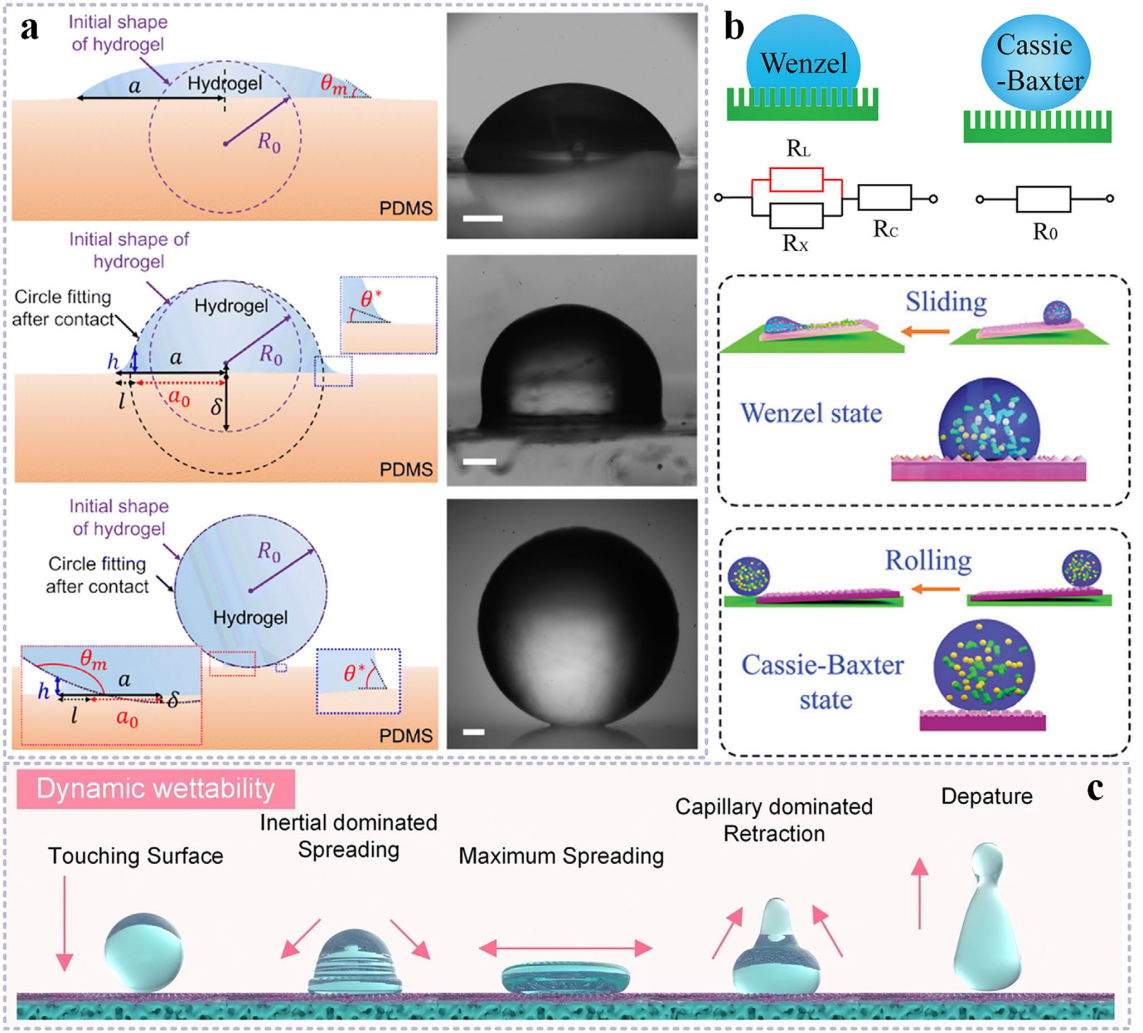
Relationship between surface contact angle, droplet type, and wettability. (a) Wettability of hydrogels with different elasticity on PDMS substrate. Reproduced with permission [47]. Copyright 2024, Royal Society of Chemistry. (b) Wetting states of Wenzel droplet model and Cassie‐Baxter droplet model on microstructure surfaces. Reproduced with permission [52]. Copyright 2020, Wiley‐VCH. (c) The dynamic wetting process of superhydrophobic droplets. Reproduced under the terms of the Creative Commons Attribution 4.0 International License [8]. Copyright 2025, Springer Nature.

Surface chemical modification regulates surface wettability, adhesion strength, and chemical reactivity by changing the surface tension and free energy of surface materials for flexible devices. Surface energy is a fundamental parameter that describes the interactions between surface molecules and directly affects the wetting behavior of liquids on the surface. Wettability refers to the ability of a liquid to spread on a solid surface, often quantified by contact angle [46]. The contact angle is directly related to the change in surface energy, and is an important indicator for measuring surface hydrophilicity, hydrophobicity, or oleophilicity (Figure 5a) [47]. The changes in surface energy and wettability not only affect the external performance of flexible electronic devices, but also play an important role in the interface adhesion ability between the device and the measured object, as well as between different functional layers.

For flexible electronic devices, surface energy regulation is usually achieved through chemical modification. Common substrate materials, such as polydimethylsiloxane (PDMS), Ecoflex, thermoplastic polyurethane (TPU), polyethylene terephthalate (PET), and polyimide (PI), typically exhibit low surface energy (e.g., the surface energy of PDMS is less than 25 mJ m^−2^), making it difficult to achieve effective adhesion with other functional layers of the device or conformal attachment to the surface of the measured object [48]. Therefore, surface chemical modification methods such as plasma treatment, ultraviolet ozone (UVO) oxidation, and chemical grafting of polar groups can significantly increase the surface energy of these substrates, thereby improving their adhesion and wettability with the measured object. Plasma treatment involves the interaction between high‐energy ions and low surface energy substrates, causing the breaking of surface atomic or molecular bonds, while introducing polar groups (such as –COOH, –OH, and –NH_2_) to enhance surface chemical activity. UVO oxidation involves the exposure of the substrate surface to UV irradiation, which breaks the molecular bonds on the surface, generating free radicals. Ozone then participates in the chemical reactions between the free radicals and surface molecules, forming functional groups such as –COOH and –OH, thereby increasing the surface energy. The common methods for chemically grafting polar functional groups include self‐assembled monolayers or organic polymerization reactions, which directly graft functional groups onto the surface of low surface energy substrates. Surface modification also involves the use of acid, base, and salt solutions to induce acid‐base reactions, redox reactions, or coordination reactions with the low surface energy substrate, thereby altering its chemical structure [49, 50]. In summary, the addition of surface polar groups alters the chemical reactivity of flexible device surfaces, thereby affecting wettability and adhesion strength.

The surface energy theory provides the fundamental theory for quantifying wetting behavior, with the Young's equation offering a quantitative framework, as shown in Equation (2):

(2) $$\cos\theta =\frac{\gamma_{s}-\gamma_{l}}{\gamma_{sl}}$$

where *θ* is the contact angle, *γ_s_* is the solid surface energy, *γ_l_* is the liquid surface tension, *γ_sl_* is the solid‐liquid interfacial tension [51]. Young's equation provides a useful baseline for evaluating hydrophilicity, hydrophobicity, and surface‐energy modification. The change in surface energy directly affects the wetting behavior of liquids on the surface. The smaller the water contact angle (WCA), the more hydrophilic the film surface becomes. The wetting behavior of planar and microstructural surfaces can be described by Cassie's law and the Cassie‐Baxter equation, which can guide the design and functionalization of flexible electronic device surfaces. According to the surface material properties, permeable Wenzel state droplets can also be formed under the same structure, while a hydrophobic surface can form the Cassie‐Baxter state droplets that do not come into contact with the conductive layer, as shown in Figure 5b [52]. The Wenzel model is suitable for rough surfaces where the liquid fully penetrates into surface microstructures, thereby amplifying the intrinsic wettability of the material. It is relevant to hydrophilic coatings, porous hydrogel surfaces, and rough elastomeric interfaces. The Cassie‐Baxter model is more suitable for micro/nanostructured hydrophobic surfaces where air pockets remain underneath the droplet, leading to high apparent contact angles and low rolling angles. It is useful for designing self‐cleaning, anti‐fouling, and liquid‐resistant surfaces. The use of high‐speed cameras can analyze the dynamic wettability and morphological evolution of droplets on superhydrophobic surfaces, revealing the depletion process of their kinetic energy, ultimately leading to droplet rebound and confirming the superhydrophobic properties of the surface (Figure 5c) [8]. In addition, water or solution also affects the adhesion of polymer surfaces, and the adhesion work can be calculated using the Dupré formula, which is applicable to ideal equilibrium adhesion between two clean phases and provides a thermodynamic estimate of the reversible work needed to separate an interface, as shown in Equation (3):

(3) $$W_{ad}=\gamma_{s}+\gamma_{l}-\gamma_{sl}=\gamma_{l}\left(1+\cos\theta \right)$$

where *W_ad_* is the thermodynamic work of adhesion, usually expressed in J m^−2^ or mJ m^−2^. When this value is less than 0, the polymer film cannot stably adhere to the surface of the object and will automatically detach [53]. However, this model does not directly include interfacial roughness, mechanical interlocking, chemical bonding, viscoelastic dissipation, plastic deformation, rate‐dependent peeling, or fatigue damage. The surface energy and wettability theory provide important theoretical frameworks for studying the surface physical properties of flexible electronic devices. The wetting angle model provides a mathematical basis for quantifying wetting behavior and helps understand the mechanisms of different surface modification methods [53]. Their combined use helps explain why plasma treatment, chemical grafting, hydrophobic coatings, and micro/nanostructures can regulate adhesion, liquid resistance, and long‐term signal stability.

#### 2.1.3 Thermomechanical Coupling and Thermodynamic Stability in Flexible Films

Under external environmental changes, the surface free energy, thermal stress, and deformation of thin films are subject to the combined effects of thermodynamics and mechanics. The thermodynamic properties of thin films determine their phase transition behavior and surface stability, and there is a direct relationship between surface tension, film thickness, and contact angle [54]. The physical parameters such as thermal conductivity, elastic modulus, and thermal expansion coefficient of flexible films directly affect the thermal‐mechanical coupling behavior of devices, such as thermal deformation, stress distribution, and surface stability. These then affect the surface physical properties and functional design of flexible devices. Thermomechanical coupling models are applicable to multilayer thin films, elastomer‐supported electrodes, encapsulated sensors, and conductive composites operating under temperature variation, Joule heating, or environmental aging. These models are particularly useful for evaluating thermal‐expansion mismatch, residual stress, local heat accumulation, and thermally induced delamination. The low melting point of metal nanoparticles can cause them to melt at lower temperatures, thereby affecting the local thermal accumulation of polymer films when metal nanoparticles are deposited in flexible thin film systems, further leading to thermal expansion and contraction of the film surface and affecting device stability [55]. Furthermore, a low elastic modulus causes deformation of the film under thermal stress, while a high thermal expansion coefficient leads to interface mismatch of the film at high temperatures, resulting in local stress and surface damage [55]. The thermodynamic behavior of films is influenced by film thickness, desorption pressure, and gravity [56]. Optimizing film parameters is a prerequisite for reducing thermal stress and improving the stability of flexible devices. For wearable and outdoor flexible electronics, these models should therefore be combined with experimental aging tests under temperature cycling, humidity, UV exposure, and repeated deformation to evaluate practical reliability.

### 2.2 Theory, Modeling, and Failure Mechanisms of Heterogeneous Interfaces

Flexible devices integrate components such as flexible substrates, electrodes, circuits, sensing layers, and packaging surfaces, and are typically mounted onto soft biological tissues like skin or onto the curved surfaces of intelligent electronic systems. This multi‐layer structure inevitably leads to stability issues in the 2D interface layer of the devices. The instability factors of the 2D interface layer mainly come from the mismatch of interface stress and the limitations of adhesion ability. Researchers have established mathematical models based on adhesion mechanics, fracture mechanics, interface creep theory, and energy balance of interface damage to study the adhesion force, interface sliding, surface fracture, and propagation mechanisms of fatigue cracks. In addition, the application of techniques such as finite element simulation and atomic force microscopy (AFM) has made it possible to achieve multiscale characterization of interfacial mechanical behavior.

#### 2.2.1 Interfacial Adhesion Theories and Models

The existing processing methods aim to improve the surface roughness of thin films and reduce the adhesion issues caused by high surface roughness. However, micron/nanometer‐scale processing variations still exist. Under the influence of size effects, some non‐covalent interactions (such as electrostatic forces and van der Waals forces) can affect the adhesion ability and contact deformation between multi‐layer films in flexible electronic devices. Researchers have refined classical adhesion theory models, including the Hertz model [33], Greenwood model, JKR model [37, 38, 39], DMT model [37, 38, 39], MD model [38, 40], and Makushkin model [41], which have been incorporated into the interfacial adhesion analysis of flexible electronic devices. For example, in microelectromechanical systems (MEMS), adhesion contact behavior under different roughness conditions, based on the Hertz model, shows that the adhesion between carbon nanotubes (CNT) and substrates is closely related to the roughness height distribution (such as Gaussian, fractal, and exponential distributions), external physical conditions (such as temperature, humidity, and electromagnetic fields), mechanical behavior, and multiscale adhesion contact mechanisms [35]. The Hertz models are suitable for estimating elastic compression between layers when adhesion is weak. Other adhesion models based on Hertz are more suitable for the adhesion behavior between flexible elastomers. The Greenwood models are useful when roughness or asperity contact determines the real contact area. The JKR model is used to describe the interfacial adhesion characteristics of high surface energy soft materials. The interface pull‐out force *P_C_* calculated by this model is shown in Equation (4):

(4) $$P_{C}=\frac{3}{2}\pi \gamma R$$

where *γ* is the surface energy [57]. Its limitation is that it assumes elastic, quasi‐static, and relatively smooth contact, and may underestimate rate‐dependent adhesion loss or hysteresis in viscoelastic materials. The DMT model assumes that the adhesive force does not affect the deformation of the object during contact, and the interfacial pull‐out force is shown in Equation (5): [58]

(5) $$P_{C}=2\pi \gamma R$$

However, it is less accurate for very soft or highly adhesive interfaces, where adhesion strongly changes the contact area. The MD model is a combination of JKR and DMT models, which is suitable for more complex nonlinear contact conditions. But it requires additional parameters related to interaction range and cohesive stress, which may limit their direct use in complex device architectures. The Makushkin model and related extended models can further account for nanoscale adhesion, surface forces, or non‐ideal interface effects, but their parameters are often difficult to determine experimentally in complex flexible devices.

Many researchers have compared the accuracy of adhesion force calculations using JKR, DMT, and MD models for different flexible material layers, through AFM measurements combined with mathematical modeling. When the phenomenon of adhesion hysteresis is considered, the adhesive forces calculated by the JKR and DMT models are relatively small. This is due to factors such as molecular reorientation at the interface, elastic or plastic deformation of the material, and diffusion between different interface layers [39]. For PDMS flexible substrates, the adhesion force and Young's modulus calculated by the JKR and MD models are closest to the experimental values [37]. Comparing the JKR, DMT, and Maugis models for predicting the adhesion ability of electrode layers (such as gold, silver, and TiO_2_ composite materials) deposited by thermal evaporation on the substrate surface of electronic devices, the JKR model is found to predict the adhesion ability more accurately [38]. When electrostatic forces are taken into account, the influence of Casimir forces on the adhesion between interfaces separated by nanoscale gaps has also been investigated [59]. Under the influence of humidity, the MD model also considers the effect of capillary adhesion on interfacial adhesion [40]. In addition, the interfacial adhesion strength (*τ*) directly affects the durability of flexible devices. A linear relationship between the number of stable cycles (*C*_n_) of crack strain sensors and τ has been established, from which a durability‐adhesion coefficient (*C*_DA_) was derived to quantify their correlation [60]. The research and improvement of mathematical models for contact adhesion in coating or film interfaces are the theoretical basis for studying interface adhesion issues in electronic devices, providing mathematical explanations for the influence of surface roughness and external conditions on interface adhesion ability. Although these adhesion models provide useful theoretical guidance, the practical adhesion of flexible electronics is also governed by testing geometry, loading rate, peeling angle, cyclic deformation, solvent swelling, sweat, and temperature. Therefore, pull‐off force, peel strength, interfacial toughness, and cycling lifetime should not be directly compared unless the measurement method and conditions are specified.

#### 2.2.2 Interfacial Stress‐Transfer Mechanisms

At the stress concentration location, when the Mises stress of the material exceeds the yield stress, the sensor will be damaged [61]. Modeling and analyzing interfacial stress transfer and related issues forms the theoretical foundation for studying the interface stability of devices. The interface mechanical behavior between wearable MEMS and human skin can be quantified by using the 2D viscoelastic model. Pressure/strain sensors receive responses from the surface of human skin. These responses are quantified as static and harmonic waves and are further used to develop physical models in different strain/stress component directions. The 2D Kelvin‐Voigt constitutive model was used to quantify the interfacial behavior between the flexible sensor and the skin surface. But its limitation is that it cannot fully describe nonlinear viscoelasticity, permanent deformation, interfacial slip, poroelastic fluid transport, or long‐term creep of hydrogels and biological tissues. Both computational and FEA confirm that the nanocomposite PVDF/BaTiO_3_ nanowire fibrous membrane of the sensor did not induce distortion in the skin at the sensor's maximum external force [62]. FEA combined with a stress transfer model can analyze in detail the changes in stress and strain on the interface layer.

In addition, the functional layer is usually deposited onto the substrate in ink or solution state through various physical and chemical methods. For fiber or porous structures such as paper or fabric, the manufacturing of composite interfaces in flexible electronic devices can also be achieved through capillary action and infiltration [63, 64, 65]. Functional materials spontaneously flow through capillary action and fully expand in paper or fabric. The capillary permeation of liquids in a substrate is related to the surface tension, dispersibility, viscosity of the liquid as well as the contact angle and microstructure of the substrate. Capillary infiltration models are applicable to porous substrates, fiber networks, textile electrodes, and paper‐based sensors, where liquid precursor transport determines interfacial contact area and bonding uniformity. In addition to the permeation mechanism, functional materials also exhibit aggregation processes on the substrate surface, which are an adsorption phenomenon. The adsorption amount is related to the material concentration. The coupling effect of interface permeation and adsorption will affect the interface stress between functional materials and substrates.

#### 2.2.3 Interfacial Sliding, Fracture, Debonding, and Failure

Interface sliding, fracture, and debonding are common issues in the long‐term operation of flexible electronic devices, ultimately leading to overall failure of the flexible devices. These interfacial issues typically occur under the influence of external loads or environmental factors, particularly stress concentration and variations in temperature and humidity, which accelerate interfacial damage. Therefore, mathematical modeling of the interfacial behavior of flexible electronic films, based on fracture mechanics and elasticity, underpins the theoretical framework for damage‐critical conditions. Their predictive power is high for well‐defined thin‐film/substrate structures, but decreases for rough, wet, chemically heterogeneous, self‐healing, or strongly viscoelastic interfaces. Interfacial sliding and fracture failure, along with interfacial delamination failure, are two common modes of interfacial failure. Interfacial sliding and fracture failure usually go through several stages. Firstly, interfacial sliding occurs, caused by interface stress exceeding adhesion force or interface toughness. Secondly, the redistribution of stress promotes crack propagation. Thirdly, the film peels off. Interfacial debonding failure is caused by the continuous separation process among different flexible layers due to insufficient interfacial adhesion energy and interfacial strength, without obvious crack propagation on the interface.

**FIGURE 6:**
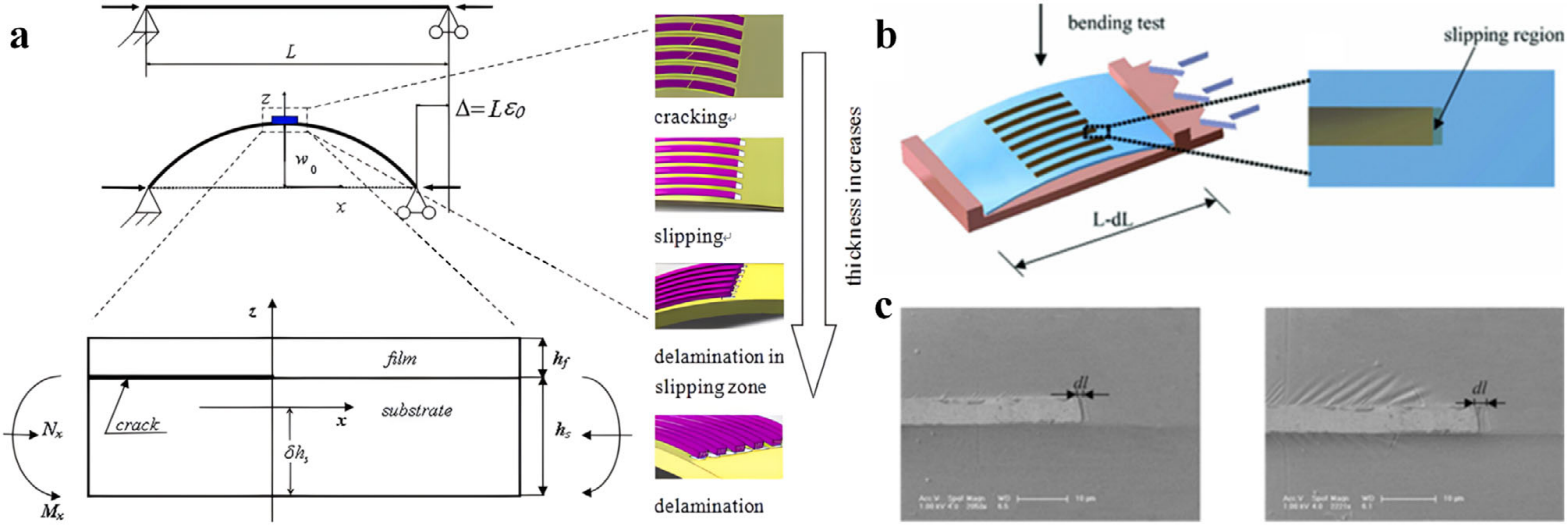
Interface models and failure mechanisms in flexible devices: (a) Interfacial failure mode of thin electronic film with thickness variation during the bending process. Reproduced with permission [67]. Copyright 2015, American Institute of Physics. (b) Sliding of thin film during bending process. (c) Scanning electron microscopy (SEM) images show the film sliding during bending. Figures 6b and 6c reproduced with permission [69]. Copyright 2011, American Institute of Physics.

The adhesion and delamination conditions of different patterned films on PDMS substrates can be used to analyze the entire process of device interfacial failure. In terms of steady‐state peeling, the energy release rate is calculated by using energy conservation and by considering self‐collapse of patterns. The pattern spacing can be adjusted to provide sufficient energy release, thus preventing crack propagation and damage [66]. The fracture of the film is the beginning of surface damage, while sliding is the first step in interfacial failure, and delamination of deposited metal thin films is further interfacial damage. Researchers have established analytical models for surface fracture and interfacial sliding failure of flexible devices, as well as mathematical models for delamination failure (Figure 6a), and obtained the critical curvature radius of the electronic layer [67].

Due to interfacial sliding being the first stage of device failure, the calculation and measurement of sliding toughness at soft interfaces are often used as important methods to verify the fatigue life of devices. When the applied strain causes the interfacial stress to exceed a certain value, the interface will slide, and the critical sliding stress is shown in Equation (6):

(6) $$|e_{\mathrm{Slip}}^{C}\left|=\right|e_{\mathrm{Delamination}}^{C}|\mathrm{if}\tau_{\max}^{0}=\tau_{c}$$

where $|\varepsilon_{\mathrm{Slip}}^{C}|$ is sliding displacement, $|\varepsilon_{\mathrm{Delamination}}^{C}|$ is the critical sliding strain, $\tau_{\max}^{0}$ is the maximum shear stress, and *τ*_c_ is the shear strength [68]. This criterion is applicable to thin‐film/substrate systems, such as rigid metal films on elastomers. From bending tests on Si/PDMS films, a fracture mechanics‐based formula for interfacial sliding toughness is derived. The critical sliding conditions, based on the energy release rate, help prevent detachment of the deposited layer from the substrate in practical applications [69].

The surface defect propagation model based on fracture mechanics, such as the Griffith theory, indicates that crack propagation occurs when the critical strain energy release rate (*G_c_*) at the interface is equal to the change in energy per unit area (*G*), as given by Equation (7):

(7) $$G=\frac{\pi \sigma^{2}a}{E}>G_{c}=2W_{ad}$$

where *σ* is the applied stress, *a* is the length of the crack, *E* is the elastic modulus, and *W_ad_* is the adhesion work. This formula can be used to analyze the propagation of interfacial cracks and further derive the interfacial failure behavior of flexible devices under different loads and environmental conditions [70]. Griffith fracture theory is most suitable for brittle or elastic materials containing a pre‐existing crack, where crack growth is governed by the balance between released elastic energy and newly created surface or interface energy. It is useful for analyzing crack propagation in deposited films, brittle conductive layers, and interfacial delamination paths. In addition to the analysis of interfacial damage caused by the deposition of rigid metal films on flexible substrates, Griffith's theory can also be used to infer the different states of interfacial crack propagation between stretchable flexible inorganic elastomers and viscoelastic substrates [71]. But its limitations are significant for soft, viscoelastic, toughened, self‐healing, or wet interfaces, because energy dissipation, crack‐tip blunting, plasticity, swelling, and chemical bonding can make the apparent fracture energy much larger than the thermodynamic adhesion work. Therefore, in flexible electronics, Griffith theory should be used together with measured interfacial toughness, cyclic fatigue tests, and environmental aging data.

Interfacial debonding is the result of the adhesion force being overcome by external mechanical forces or environmental factors such as temperature, humidity, and stress concentration. Insufficient interfacial adhesion may cause detachment among films of different layers. The work done by the adhesive interface peeling (*W_p_*) can be calculated by the peeling of the heterogeneous film, as shown in Equation (8):

(8) $$W_{p}=U+\Phi$$

where *U* represents the elastic bending energy stored in the film and Φ is the energy overcoming the interfacial interaction potential [72]. Peeling models are applicable to thin flexible films, adhesive interlayers, transfer printing, and encapsulation layers where separation occurs progressively from an edge or crack front. The peeling force can also reflect that the bonding and debonding ability of the interface is related to the bending stiffness of the heterogeneous film. The interfacial bonding area can be expressed by the cohesive law of constant‐stress [73]. As shown in Figure 6b, different interfaces have different strengths and separation distances [69]. When the interface exceeds the critical separation distance, it represents interfacial separation, debonding, and interfacial failure. Furthermore, the interfacial strength ratio, membrane elastic modulus ratio [74] and the film thicknesses of the upper and lower layers at the interface [75] will significantly affect the path of interfacial delamination. However, peel strength depends strongly on peeling angle, loading rate, film thickness, bending stiffness, viscoelasticity, and environmental conditions. Therefore, values obtained from 90° peel, 180° peel, T‐peel, or lap‐shear tests should not be treated as directly interchangeable. In summary, a theoretical basis can be provided for the prevention of interfacial failure, avoiding interfacial fracture and delamination issues in practical applications.

#### 2.2.4 Finite Element Simulation and Experimental Characterization of Interfacial Behavior

Researchers not only establish mathematical models to analyze interfacial behavior, but also combine FEA and AFM to further clarify the types and locations of interfacial damage. FEA shows that interfacial damage in physical vapor deposition (PVD) films arises from the mismatch in elastic moduli at the heterogeneous interface. Adding a transition layer, like an anodized film, improves bonding and reduces stress concentration, while also guiding experimental optimization [76, 77]. However, FEA results should be interpreted carefully because they depend on the accuracy of material parameters, mesh quality, interfacial boundary conditions, and damage criteria. In many soft devices, viscoelasticity, swelling, frictional sliding, and environmental aging must be included to obtain realistic predictions. In addition to finite element analysis and contact‐mechanics modeling, computer‐vision‐based methods provide a useful route for quantifying coupled mechanics in skin‐interfaced electronics [78]. AFM tip loading measurements are commonly used to measure the adhesion and sliding behavior of films and deposited layers. As shown in Figure 6c, laser scanning confocal microscopy can be used to measure the spontaneous buckling dimensions of the material, enabling the calculation of the interfacial fracture energy of the buckling [69, 79]. Nanoindentation technology can be used to detect the interfacial stress of flexible devices, revealing the relationship between the mechanical stability and the magnitude of interface stress [80]. Analyzing the adhesive force at interfaces of different films, exploring various interfacial damage situations, and combining mathematical model solving, FEA, and characterization testing are the theoretical basis for solving interfacial issues in flexible electronic devices, providing conditions for seeking suitable materials and reducing interfacial damage schemes.

Overall, no single physical model can fully describe all surface and interfacial behaviors in mechanically coupled flexible electronics. A rational theoretical framework should combine contact mechanics for surface coupling, wetting and adhesion theory for surface‐energy regulation, viscoelastic and stress‐transfer models for soft interfaces, fracture and peeling models for interfacial failure, and FEA/experimental characterization for multiscale validation. This model‐selection strategy helps connect fundamental theory with practical device reliability, including reduced signal drift, suppressed hysteresis, delayed delamination, and improved stability under cyclic and environmental loading.

## 3 Surface and Interfacial Challenges in Mechanically Coupled Flexible Electronics

### 3.1 Surface Challenges in Mechanically Coupled Flexible Electronics

#### 3.1.1 Surface Self‐Adhesion

**FIGURE 7:**
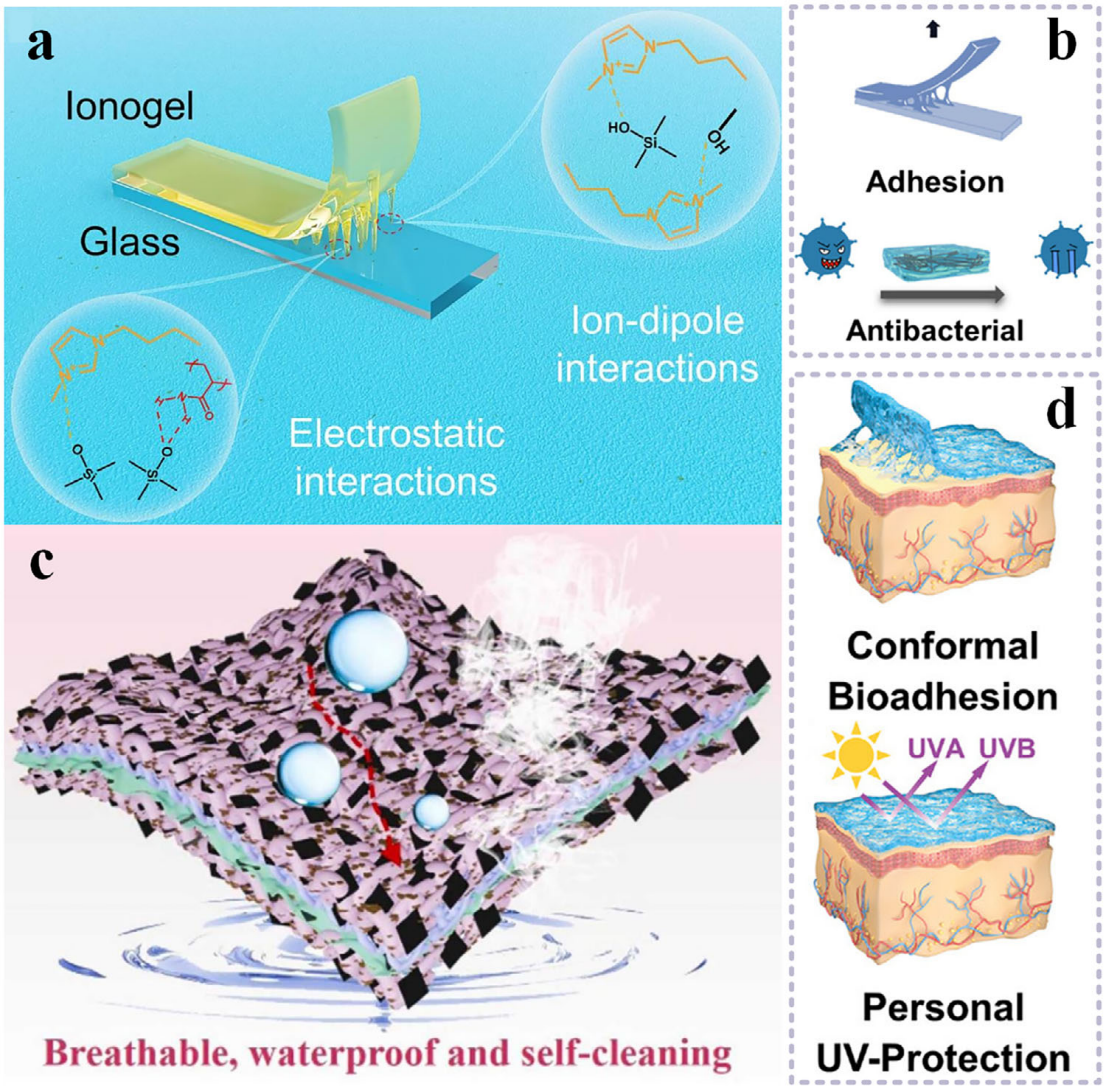
Surface issues of flexible devices: (a) Adhesion between the surface of flexible devices and glass. Reproduced with permission [82]. Copyright 2023, Elsevier Ltd. (b) Surface antibacterial activity of flexible devices. Reproduced with permission [88]. Copyright 2023, American Chemical Society. (c) Breathable, waterproof, and self‐cleaning of flexible fabric devices. Reproduced with permission [92]. Copyright 2023, Elsevier Ltd. (d) Adhesion and UV protection ability between flexible substrates and biological surfaces. Reproduced with permission [94]. Copyright 2024, Wiley‐VCH.

Mechanically coupled flexible electronics used for measuring physical vibrations and deformation signals require high conformability with the measured object. However, in the application of various mechanical sensing devices, sensors are usually attached to surfaces such as metal, human skin [81], fabrics, building materials, rubber, and glass (Figure 7a) [82]. Fixing the sensors only with tapes can cause them to deviate, fall off, or fail to conform when the measured object undergoes mechanical changes, resulting in signal offset, hysteresis, or distortion. Therefore, it is important to enhance the surface adhesion of flexible mechanical devices through chemical or physical methods, and overcome external temperature, humidity and mechanical changes.

The specific requirements for the surface self‐adhesion of flexible mechanical devices include the following: (1) The ability to achieve adhesion on surfaces of materials with different surface energies and structural characteristics. (2) The ability to repeatedly adhere and peel off while maintaining high adhesion strength. (3) The effect of high and low temperatures on the surface adhesion strength of the device. (4) The effectiveness of surface adhesion strength under exposure to water, sweat, lubricating oils, or various beverages. (5) The potential for the adhesive material or physical adhesion to cause contamination, structural damage, or health risks to the surface of the measured object. (6) The impact of physical or chemical adhesion on the testing performance of the flexible mechanical device.

#### 3.1.2 Surface Self‐Cleaning and Anti‐Fouling

When applied in human health monitoring, mechanically coupled flexible electronics are exposed to environments with sweat, water, saline solutions, and even microorganisms, which can affect the surface adhesion and sensing performance of the device. Therefore, the surface of flexible devices needs to possess self‐cleaning capabilities. Environmental stability strategies for these surfaces can be understood through several common mechanisms, including hydrophobic coatings, anti‐fouling surfaces, liquid‐resistant microstructures, antibacterial interfaces, anti‐freezing and moisture‐retention systems, and protective encapsulation. These designs reduce external interference from water, sweat, oil, dust, and biological contaminants. For example, the surface of the device needs to have hydrophobic properties, with a contact angle greater than 90°, and a hydrophobic effect on various beverages such as milk, coffee, cola, and tea, while the rolling contact angle is less than 10° [83, 84]. The excellent hydrophobicity allows dirt and dust on the surface of the device to be easily cleaned by liquid [85]. The surface of flexible devices has certain resistance to liquid interference and bacterial adhesion, which can not only prevent liquid from penetrating into the device and affecting sensing performance, but also prevent bacterial invasion (Figure 7b). Otherwise, the device may detach or slide off from the human skin or clothing [52], posing a potential health risk [86, 87, 88, 89]. In equipment monitoring, construction, and aerospace applications, flexible mechanical devices are exposed to more complex operating conditions. These include lubricating oils, construction dust, low temperatures, strong wind and rain, rubber softening, and contamination on vehicle tires. With oleophobic properties, resistance to liquid interference, and ice prevention on the surface, these flexible devices can ensure their reliability in complex operating environments. Therefore, the self‐cleaning ability of flexible mechanical devices to resist interference of water, oil, acid, alkali, and microorganisms on their surfaces is crucial.

#### 3.1.3 Surface Aging and Environmental Degradation

The device may be subject to freezing, drying, high temperature, and strong ultraviolet (UV) radiation, which will cause the device surface material to age [90]. It is essential to treat the device surface material with anti‐oxidation, anti‐UV, high‐temperature, freeze, and moisture resistance. For example, for the hydrogel‐based mechanical sensor, the internal water will freeze at low temperatures, significantly affecting its mechanical properties and conductive sensing ability. The use of salt solutions, organic solvents, and ionic liquids (ILs) to suppress water crystallization is a method of resisting aging [91]. Dust pollution and harmful liquid invasion can occur due to the inherent porous structure of fabric sensors. The device will be penetrated by sweat and affected by secreted lipids when applied in human health monitoring (Figure 7c) [92]. Some easily oxidizable materials such as Ag and Cu in flexible sensors may undergo oxidation failure without surface protection or composite and modification of conductive materials. In addition, strong UV radiation can cause photodegradation of some flexible polymer materials and damage their surface adhesion ability, leading to detachment and damage of flexible devices [93]. When applied to human health monitoring, it can also cause skin photodamage and photoaging due to poor protective performance of sensors (Figure 7d) [94].

### 3.2 Interfacial Challenges in Mechanically Coupled Flexible Electronics

#### 3.2.1 Interfacial Adhesion

**FIGURE 8:**
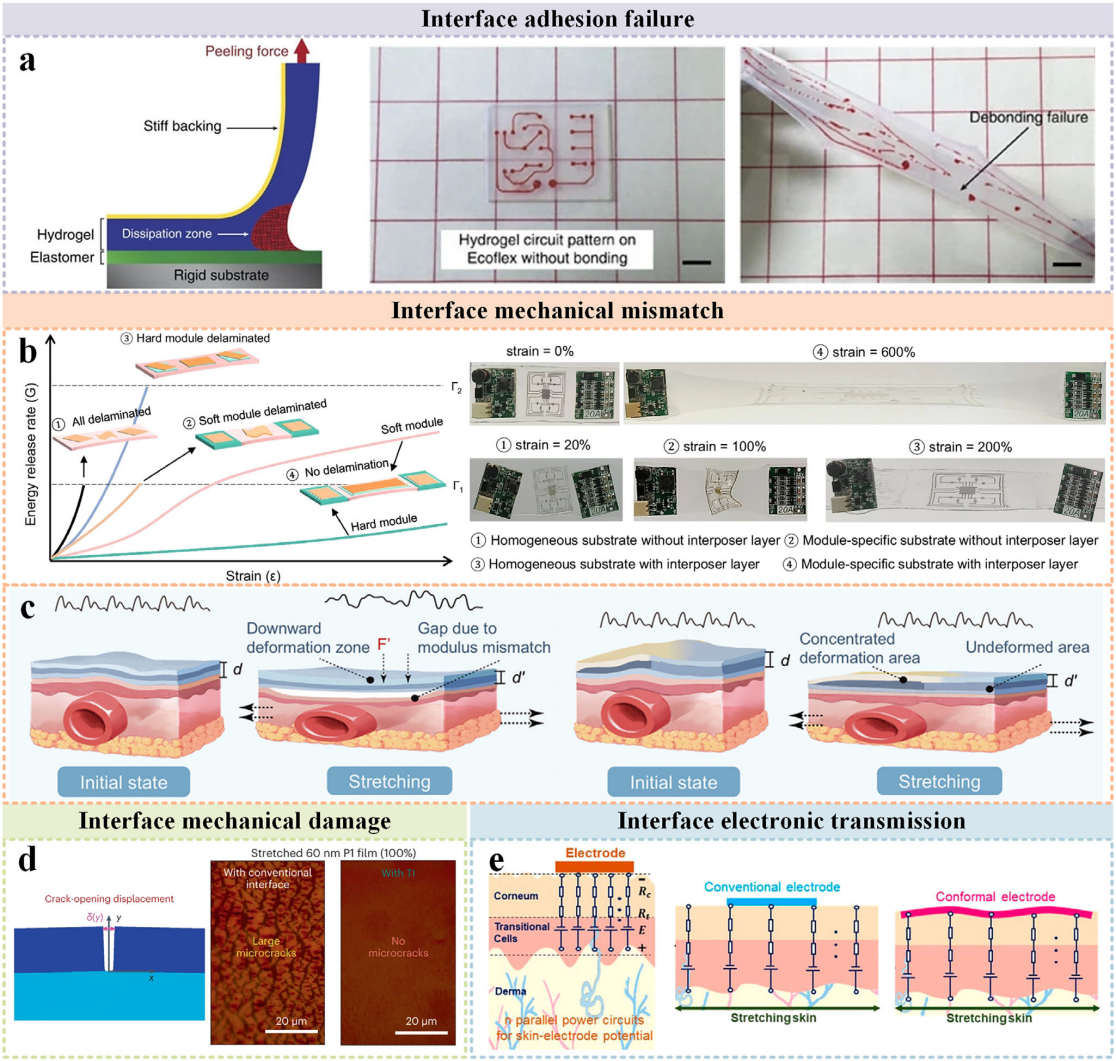
Interface issues of flexible electronic devices: (a) Delamination and failure of flexible electronic devices without interfacial bonding treatment after stretching. Reproduced under the terms of the Creative Commons Attribution 4.0 International License [95]. Copyright 2016, Springer Nature. (b) Interfacial toughness of the device with or without adhesive and interfacial layer. Reproduced under the terms of the Creative Commons Attribution 4.0 International License [101]. Copyright 2024, Springer Nature. (c) Signal error of devices with different modulus designs. Reproduced under the terms of the Creative Commons Attribution 4.0 International License [16]. Copyright 2025, Wiley‐VCH. (d) Optical images of film cracks in flexible mechanical devices and schematic of multi‐layer crack structure. Reproduced with permission [18]. Copyright 2022, Springer Nature. (e) Electrical signal transmission mechanism of rigid and conformal electrodes on skin. Reproduced with permission [109]. Copyright 2020, American Chemical Society.

As shown in Figure 8a, flexible electronic devices without any interfacial bonding treatment experience delamination, electrode cracking, and even circuit failure under tensile stress [95]. Even with simple packaging, it is inevitable for the internal layers of the device to experience displacement and even damage to the device structure when subjected to external forces. 3M tape is commonly used as an adhesive layer to tightly contact multiple layers of films at the interface of flexible devices [96, 97]. However, tape does not have long‐term adhesion to low surface energy films, and insufficient interface toughness can also lead to debonding. As an adhesive, anisotropic conductive adhesive can effectively bond multi‐layer devices, but it will lose its adhesion in high‐humidity environments, leading to device separation [98]. Changing the surface energy of the substrate is a potential solution, but it poses the following challenges: (1) Ideal adhesive materials require excellent interfacial adhesion to each layer simultaneously, and there is no need to develop complex and demanding coating processes for different adhesive materials. (2) Some processes involving high‐energy physics can cause thermal losses to polymer substrates. In addition, interfacial interlocking and adsorption can be achieved through structural design such as biomimetic physical adsorption structures, but the manufacturing process of these structures is complex or has precise requirements for the curing stage. In conclusion, using different interfacial bonding methods or developing universal strategies are key to solving the adhesion problems in flexible electronic devices.

#### 3.2.2 Interfacial Mechanical Mismatch

The interface between sensors and complex surfaces such as human skin is non‐conformal, which can lead to poor contact and unreliable interface [99]. The multi‐layer sensor is composed of rigid or brittle electrodes or sensing materials deposited on a flexible and stretchable substrate, with a large difference in Young's modulus between them. In the stretch state, the tensile strain of the rigid film exceeds the yield strain, making it prone to fracture [100]. Even if the interfacial adhesion force is increased, stress concentration still exists between the soft and hard interface. The energy release rate of the interface increases monotonically with the degree of modulus mismatch after stretching, leading to delamination failure. As shown in Figure 8b, depending on the different interfacial mechanical conditions and the presence of an interfacial mechanical release layer and adhesive layer, different types of delamination occur at the device interface in the stretch state [101]. The heterogeneous material interface between integrated devices can lead to stress concentration and fracture of conductive materials under high stretch due to mechanical mismatch [102]. Furthermore, mechanical sensors can produce motion artifacts (MAs) when subjected to stress, as shown in Figure 8c. Low modulus, strain‐sensitive materials generate normal stress and additional signals from abnormal vibrations, while high modulus materials hinder force transmission and cause gaps at the interfaces [16].

#### 3.2.3 Interfacial Mechanical Damage

When mechanically coupled flexible electronics are subjected to external wear and scratches, surface cracks may appear. As the mechanical device is continuously loaded, these cracks and surface wear will gradually expand, forming microcracks, as shown in Figure 8d [18]. For mechanical sensors with microcrack mechanisms, sensing can be achieved through uniform cracks, but there are high demands for the stability and mechanical properties of the materials. Designing self‐healing materials is one way to solve device damage, and the main mechanisms are divided into two categories: the release of microencapsulated self‐healing agents and the design of self‐healing materials with dynamic cross‐linking. The microcapsule method uses capillary action to reach the crack for repair, but only one repair can be achieved for the same location [103]. Self‐healing materials with dynamic covalent and non‐covalent interactions are currently a popular method for solving surface damage [104, 105]. However, the proportion control of crosslinkers and chain extenders not only determines the self‐healing efficiency, but also has strict requirements for self‐healing conditions such as temperature, pressure, and humidity, which also affect the mechanical properties of the device. Combining good mechanical and self‐healing properties is the key to solving the issue. After the formation of microcracks in the films, interfacial mechanical damage will continue to occur under complex operating conditions. After the complete rupture of a single‐layer film, crack propagation and interfacial damage will occur in the multi‐layer structure of the device [18].

#### 3.2.4 Interfacial Electronic Transport

When different components of flexible devices are connected, the inherent differences in physical and chemical properties inevitably result in bonding strength and ohmic contact at the interface. When adhesives are used to enhance the interfacial bonding strength, the introduction of a new interface may significantly alter the electrical behavior, increase total resistance, or alter Schottky barriers [106]. The resistance effect of resistive flexible mechanical devices is affected by the tunneling effect, contact resistance, and cracks when the single‐layer conductive layer is strained, while the interfacial contact resistance of multi‐layer sensors also changes in the electronic channel when subjected to external forces, thereby affecting electron transfer [107]. A stable interface is a prerequisite for improving the interface resistance effect. Capacitive flexible mechanical devices need to improve their capacitance response to avoid external noise interference. Although nanoscale dielectric layers can enhance signal response, they have problems such as decreased mechanical performance and reduced charge storage capacity due to electron tunneling effects [108]. In addition, the occurrence of MAs when flexible electronic devices are attached to curved surfaces such as skin is due to the potential difference between the skin and external electronic devices being influenced by the skin's metabolism. The electric model of the skin established by Talhouet and Webster confirms the relationship between electrode area and voltage, as shown in Figure 8e. As the skin stretches and its area increases, flexible devices expand accordingly, while rigid devices' electrode area remains unchanged. This alters the output voltage and results in fluctuating interfacial current transfer [109].

## 4 Material‐Driven Strategies for Surface and Interfacial Enhancement

### 4.1 Intrinsic Functional Materials

Some materials inherently possess self‐adhesion, self‐healing, hydrophobicity on the surface, and also have flexibility or extensibility. These materials can be used as the base and conductive layer of flexible mechanical devices, including multifunctional hydrogels, liquid metals (LM), and other polymers with low surface energy.

#### 4.1.1 Hydrogels

Hydrogels exhibit good biocompatibility and are widely used in flexible mechanical sensors. Their self‐adhesion, self‐healing, high robustness, and conductivity can be regulated by internal crosslinkers, dynamic covalent bonds, and non‐covalent interactions. Their development has become a popular trend in the application of flexible mechanical sensors. According to the dominant interfacial interactions, adhesive hydrogels and organohydrogels can be broadly categorized into hydrogen‐bonding, electrostatic‐interaction, ionic‐interaction, metal‐coordination, and hydrophobic‐association systems. This mechanism‐based classification helps explain why different gel networks are suitable for different substrates, contact conditions, and service environments.

**FIGURE 9:**
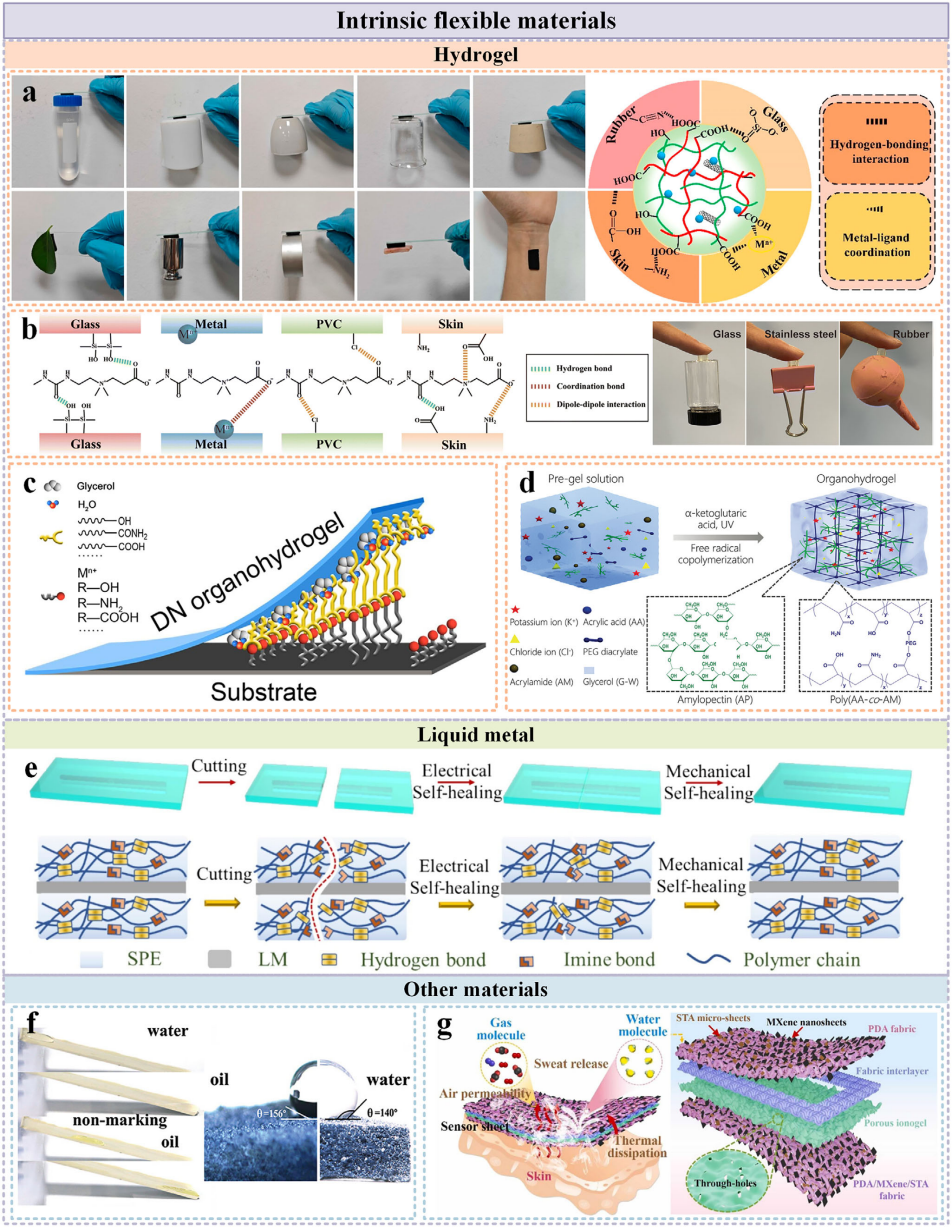
Intrinsic flexibility material design: (a) Self‐adhesion mechanism of PVA/PAA‐Al‐cCNTs hydrogel. Reproduced with permission [110]. Copyright 2024, American Chemical Society. (b) Surface adhesion ability and mechanism of PCBUIA hydrogel. Adapted with permission [113]. Copyright 2024, Royal Society of Chemistry. (c) Self‐adhesion mechanism of PVA_α_‐GA/P(AA_X_‐*co*‐Am_y_). Reproduced with permission [117]. Copyright 2021, Elsevier Ltd. (d) Surface adhesion mechanism of Poly(AA‐*co*‐AM)/AP hydrogel. Reproduced with permission [120]. Copyright 2021, Wiley‐VCH. (e) Self‐healing mechanism of flexible strain sensor prepared by LM and self‐healing PDMS. Reproduced with permission [130]. Copyright 2024, Elsevier Ltd. (f) Water and oil droplets on the surface of PEDOT:PSS/SrTiO_3_ foam with TPU coating exhibit contact angles of 140° and 156°, respectively, demonstrating the hydrophobic property of the TPU coating. Reproduced with permission [133]. Copyright 2024, Wiley‐VCH. (g) Hydrophobic effect of STA on the surface of textile sensor. Reproduced with permission [92]. Copyright 2023, Elsevier Ltd.

A self‐adhesive hydrogel containing metal coordination bonds and hydrogen bonds was prepared by adding acrylic acid (AA) to a polyvinyl alcohol (PVA) solution, followed by the incorporation of Al^3+^, cCNTs (carboxylated multi‐walled carbon nanotubes, MWCNT‐COOH), and ammonium persulfate. This hydrogel, named PVA/polyacrylic acid (PAA)‐Al‐cCNTs, forms hydrogen bonds with nitrogen groups in biological tissues and rubber, and coordination bonds with metal oxides, exhibiting strong adhesion to various substrates (Figure 9a). The presence of cCNTs also enhances its conductive sensing properties [110]. A *β*‐CD‐*g*‐(pAAm/pHMAm) hydrogel was prepared by polymerizing acrylamide (AAm) and *N*‐hydroxy methyl acrylamide (HMAm) with *β*‐cyclodextrin (β‐CD), which exhibits surface adhesion due to multiple hydrogen bonds and non‐covalent interactions [111]. Another hydrogel was prepared by incorporating ILs and tannic acid (TA) into an AAm and poly(ethylene glycol) diacrylate (PEGDA) copolymer. Hydrogen bonding and ionic interactions endow this network with self‐adhesion. TA forms a strong adhesive complex with iron, resulting in an adhesion strength of up to 97 kPa [112]. The zwitterionic monomer was synthesized and UV‐cured into a hydrogel, which exhibits high adhesion strength to various substrates due to hydrogen bonding and electrostatic interactions, as shown in Figure 9b [113]. Hydrogels were prepared by incorporating various imidazolium‐based ILs into lipoic acid, exhibiting excellent mechanical and self‐adhesive properties due to hydrogen bonding, cation–π and π–π interactions, and electrostatic interactions [114]. Adding glycerol can inhibit the evaporation and crystallization of water molecules in the hydrogel, thereby enhancing the freeze resistance of the sensor and preventing material aging [91]. At the device level, this anti‐freezing effect helps maintain ionic conductivity and softness under low‐temperature operation, thereby reducing sensitivity loss and baseline drift caused by hydrogel stiffening or water crystallization. The conductive hydrogel PBSTCE, composed of PVA, starch, and tea polyphenol (TP), exhibits surface self‐adhesion due to hydrogen bonding and dynamic borate ester linkages. The ethylene glycol (EG) solvent provides freeze resistance, while TP reduces and stabilizes silver nanoparticles (AgNPs), endowing PBSTCE with antibacterial activity against Staphylococcus aureus and Escherichia coli [115]. Such combined anti‐freezing, antibacterial, and adhesive functions are beneficial for wearable sensing in sweaty or contaminated environments, where unstable adhesion and microbial contamination can lead to signal drift and poor long‐term reproducibility. Hydrogel‐based flexible electronics should not be judged solely by sensitivity. Stable long‐term motion monitoring requires a balance of adhesion, self‐healing, anti‐freezing, moisture retention, antibacterial activity, and fatigue resistance. Optimizing one property at the expense of others can still cause signal drift, hysteresis, or reduced cycling stability in practice. The hydrogel prepared by using bacterial cellulose nanowhisker (BCW), TA, PAA, Fe^3+^, and glycerol/water exhibits both self‐adhesion and self‐healing properties due to the multiple hydrogen bond interactions [116]. The double network hydrogel PVA_α_‐GA/P(AA_X_‐*co*‐Am_y_), composed of glutaraldehyde cross‐linked PVA (PVA‐GA), poly(acrylic acid‐*co*‐acrylamide) (P(AA‐*co*‐Am)) and KCl, features non‐covalent interactions that provide adhesion, as shown in Figure 9c [117]. The hydrogel sensor prepared by adding sodium alginate (SA) and TA to PVA and using TA to modify BaTiO_3_ nanoparticles has high adhesion strength due to the dynamic borate ester bond and hydrogen bond interactions [118]. Furthermore, in the design of nano‐composite hydrogels, poly(N‐isopropyl acrylamide‐co‐acrylamide) (P(NIPAAm‐*co*‐Am)) was added to PVA, along with Fe_3_O_4_ and LiCl. Under heating conditions, the hydrogel undergoes a phase transition, enabling ionic conductivity, while the adhesion properties of the hydrogel can be regulated by adjusting the temperature [119]. By regulating the glycerol content and altering the adhesion strength of Poly(Acrylamide‐*co*‐Acrylic acid) (Poly(AA‐*co*‐AM))/Amylopectin (AP) hydrogel, a large number of glycerol molecules bond with water molecules, weakening the hydration effect of the polar groups within the polymer chain. More exposed surface polar groups enhance the adhesion (Figure 9d) [120]. The phase separation mechanism can also be used to construct gel materials with alternating hard and soft phases, enabling them to have both a hard phase for energy dissipation and a soft phase for resisting stress. A phase‐locked composite architecture with alternating soft‐hard domains was constructed from 2‐hydroxyethyl methacrylate and cellulose/ILs phase‐separated networks. This structure relies on competitive hydrogen‐bonding‐induced phase separation. Abundant hydrogen‐bonding interactions allow the material to form robust and stable adhesion with the contacted substrate. Under mechanical loading, the heterogeneous network redistributes interfacial strain across the soft and hard domains.This behavior helps maintain synchronous contact with human skin and improves environmental stability [121]. This type of soft‐hard phase regulation is particularly useful for reducing MAs. Strong adhesion maintains conformal contact with skin, while the soft‐hard network helps dissipate repeated mechanical loading and suppress fatigue‐induced deformation. This balance is especially important for wearable sensors that must operate during continuous joint movement, sweating, and environmental temperature changes.

In summary, by tuning monomer types and ratios and optimizing solvents and ILs, hydrogels can be engineered to incorporate abundant non‐covalent interactions, dynamic covalent bonds, and metal coordination bonds. These features intrinsically endow the materials with strong surface adhesion, hydrophilic or hydrophobic self‐cleaning, antibacterial activity, and self‐healing capability, making them promising intrinsic materials for enhancing the interfacial performance of flexible mechanical devices. For practical devices, these properties translate into more stable pressure, strain, and electrophysiological signals by reducing contact loss, interfacial slipping, and deformation‐induced conductive‐pathway disruption. Nevertheless, the intrinsic limitations of hydrogels cannot be overlooked. In flexible electronic applications, hydrogels are prone to permanent deformation under long‐term and repeated mechanical loading, such as stretching and bending, which progressively degrades their structural integrity and compromises device durability. Furthermore, the high water content that imparts excellent softness to hydrogels also renders them vulnerable to dehydration in ambient environments. The resulting water loss leads to reduced compliance and causes performance drift in the corresponding devices. Beyond the intrinsic properties of hydrogel networks, their interfacial compatibility with conductive fillers is also critical for stable sensing. Conductive components such as carbon nanotubes, silver nanoparticles, MXene sheets, and liquid metals must be uniformly anchored within the hydrogel matrix to avoid aggregation, interfacial slipping, and conductive‐pathway discontinuity. These challenges significantly undermine the long‐term operational stability and reliability of hydrogel‐based flexible electronics, thereby limiting their effectiveness in addressing critical surface and interfacial issues. Therefore, hydrogel‐based strategies should be evaluated not only by adhesion strength or sensitivity, but also by signal drift, hysteresis, performance retention, and fatigue resistance under repeated deformation and environmental exposure.

#### 4.1.2 Liquid Metals

LM possesses low melting points and weak intermolecular interactions, which endow it with high fluidity. Therefore, when surface cracks occur in electrodes made of LM, the cracks will be filled under the drive of surface tension, achieving self‐healing ability. During the stirring process of LM within polymer materials, it exhibits non‐Newtonian flow characteristics, namely, under specific shear rates, a shear‐thinning phenomenon occurs [122]. Especially, the viscoelasticity of the oxide layer of the LM enables stress transfer and alleviation at the interface with other materials [123, 124]. Furthermore, in the presence of solid microparticles, the high surface tension of LM helps maintain the structural stability of the material after healing, a feature that has been widely exploited in flexible electronics and stretchable conductors. The most widely used LMs include eutectic gallium‐indium (EGaIn), eutectic gallium‐indium‐tin (Galinstan), and other gallium‐based LMs. The fluidity of LM enables it to form a soft/soft interface with the flexible substrate and effectively alleviates the instability problem of the composite material caused by the mismatch of modulus [125]. The strain sensor fabricated on the TPU surface using the printing method with an elastic modulus of 0.4 MPa can be stretched to the elongation rate required for TPU applications [126]. The 3D‐printed PVA‐LM composite material is stabilized at the sensor interface on the PVA substrate [127]. When LM is mixed with PDMS to embed the LM, the ink exhibits shear‐thinning properties and can be used for manufacturing electrodes [128]. The high viscosity of the non‐eutectic GaIn alloy enables it to adhere to the Ag/AgCl electrode [122]. A flexible strain sensor was prepared by mixing Galinstan with Ecoflex. The resulting mixture has excellent fluidity and high conductivity. Ecoflex can also prevent the LM particles from melting [129]. EGaIn was injected into the self‐healing PDMS using the negative pressure injection method to form a flexible strain sensor with three‐dimensional microchannels. It can achieve self‐ healing even when subjected to micro‐damage on the surface and interface, as shown in Figure 9e [130]. A 2, 2, 6, 6‐tetramethylpiperidin‐1‐oxyl (TEMPO)‐oxidized lignin‐LM material was designed to form a stable interface with PAA hydrogel. This modified lignin forms ionic and electrostatic interactions with the Ga_2_O_3_ layer on the EGaInSn surface, effectively preventing the aggregation of LM in the polymer and enhancing the conductivity of the sensor [131]. Polymer chains, hydrogel networks, nanocellulose, MXene sheets, or other fillers can confine liquid‐metal droplets and improve their dispersion. By stabilizing the LM interface within the polymer or hydrogel matrix, these designs help suppress aggregation, leakage, and discontinuous conductive pathways, which are common causes of resistance fluctuation and output drift during cyclic deformation [132]. In summary, the integration of LMs with other materials in flexible mechanical sensors can effectively alleviate soft‐hard interfacial mismatch while enabling self‐healing upon damage, making them promising intrinsic materials for addressing interfacial challenges in flexible electronic devices. However, LM systems still require careful interfacial confinement, because oxidation, leakage, and weak bonding with elastomers or hydrogels may increase hysteresis and reduce long‐term cycling reliability.

#### 4.1.3 Other Intrinsic Materials

Some materials have surface energies lower than 30 mN m^−1^ and possess hydrophobic, anti‐adhesive, and self‐cleaning properties. Common examples of such intrinsic materials include fluoropolymers, silicone rubbers, resins, and polyolefins. These materials generally exhibit weak intermolecular interactions. For instance, in fluoropolymers, although the C–F bond is highly polar, the small atomic radius of fluorine and the highly symmetric molecular structure of polytetrafluoroethylene (PTFE) result in an almost negligible net dipole moment and low cohesive energy density. Similarly, in silicone rubbers such as PDMS, the Si–O bonds possess high bond energy and long bond lengths, while intermolecular interactions are dominated by weak van der Waals forces. Moreover, the dense surface arrangement of methyl groups forms a low‐surface‐energy surface. These materials, as the base and encapsulation coating for flexible sensors, can effectively address the surface and interfacial issues of the devices. For device operation, low‐surface‐energy materials mainly improve reliability by reducing liquid contamination, surface fouling, and environmental interference at the sensing interface. This can stabilize contact resistance and capacitance response in wet, dusty, or chemically complex environments.

The TPU coating has excellent hydrophobic properties. It can prevent the penetration of water and oil on the surface of the poly(3, 4‐ethylenedioxythiophene):poly(styrenesulfonate) (PEDOT:PSS)/SrTiO_3_ foam structure, as shown in Figure 9f, where the contact angles of oil and water are greater than 120° [133]. By dip‐coating polydopamine (PDA)‐modified MXene onto textile substrates, the conductive nanosheets can be firmly anchored within the fabric architecture via strong hydrogen‐bonding interactions, as shown in Figure 9g. Subsequently, hydrophobic stearic acid (STA) is spray‐coated onto the surface of the textile sensor. Owing to its intrinsic hydrophobicity, STA effectively suppresses MXene restacking and oxidation, while simultaneously imparting waterproofing, sweat resistance, and self‐cleaning capability, thereby enabling the sensor to operate reliably in underwater monitoring applications [92]. A layer of MWCNTs and a layer of organosilicon dioxide (o‐SiO_2_) were successively deposited on the surface of the natural latex film, serving as the sensing layer and the superhydrophobic surface layer, respectively. Through the combination of the surface microstructure caused by swelling and the vinyl groups on the surface of o‐SiO_2_, the surface achieved superhydrophobicity. Even when immersed in solutions of different pH values and different concentrations of salt for more than 24 hours, this property remained intact, thus being applicable in underwater sensing fields [134]. Such waterproof, antifouling and drag‐reducing surfaces are useful for dynamic fluid environments, because they reduce surface contamination and mechanical disturbance that may otherwise cause unstable signal output. The glass transition temperature of PI is greater than 300°C, while that of CNT is greater than 500°C. The sensor prepared by electrophoretic deposition of CNT onto PI fibers can still detect touch signals from fingertips at 249°C. The inherent properties of the material enable the sensor to withstand high‐temperature environments and delay device aging [135]. The PI‐based sensor containing silver nanomixes (AgNMs) and silver microflakes (AgMFs) can also be used stably for 1000 cycles when operated at 250°C [136].

### 4.2 Self‐Healing Materials

By incorporating dynamic covalent bonds and non‐covalent interactions, crosslinked networks or healing‐agent/matrix composites can be engineered to enable self‐healing under diverse conditions, including room temperature, elevated temperature, and high humidity. This capability suppresses interfacial cohesive failure caused by bending, stretching, or impact, thereby preventing microcrack formation and fracture at device interfaces. In terms of device output, self‐healing interfaces can reduce fatigue‐induced resistance drift, signal discontinuity, and sudden failure caused by microcrack accumulation. Consequently, self‐healing materials effectively mitigate mechanical damage and electrical degradation, improving the durability, reliability, and service lifetime of mechanically coupled flexible electronic devices.

**FIGURE 10:**
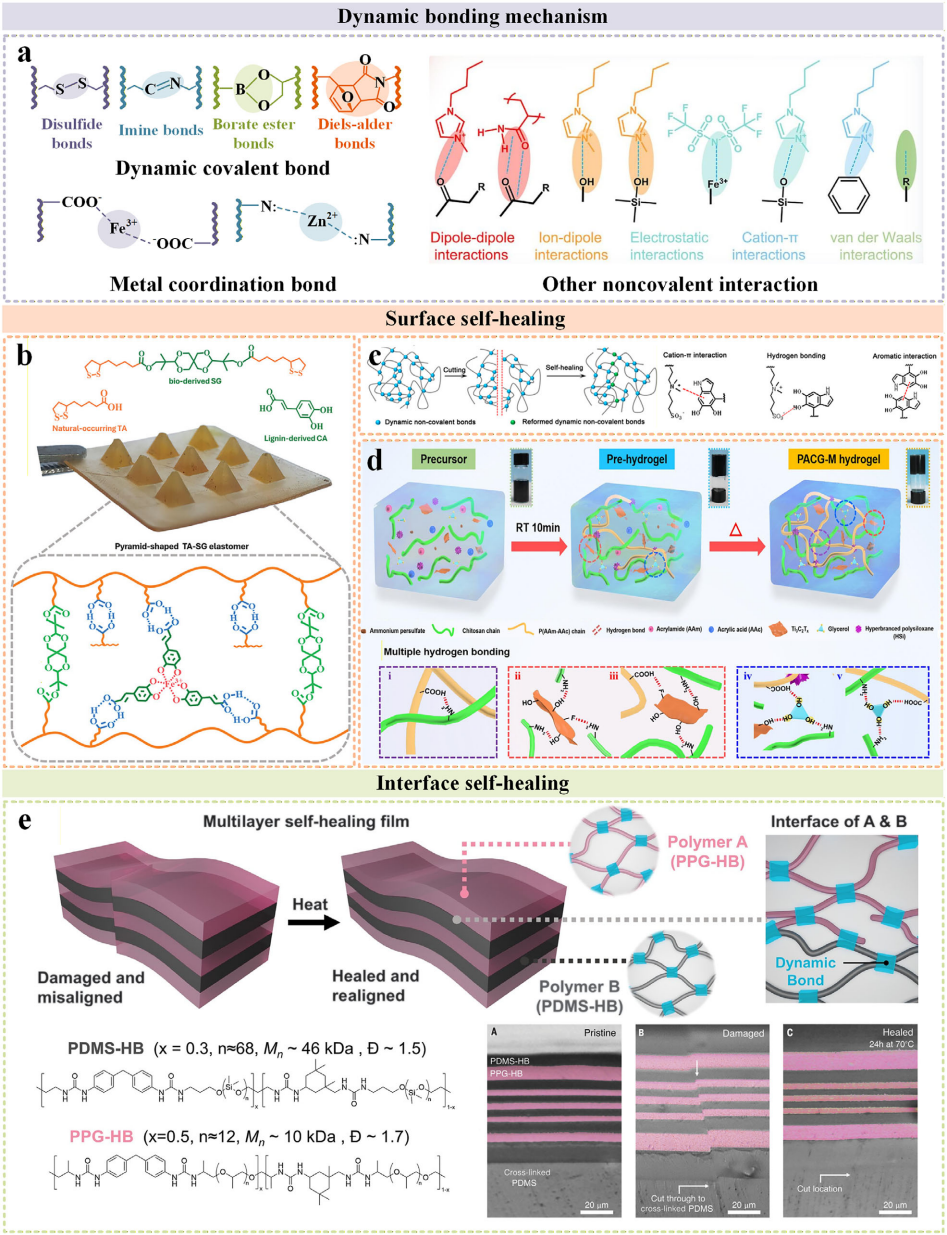
Dynamic bonding mechanisms: (a) Various dynamic bonding mechanisms. Reproduced with permission [82]. Copyright 2023, Elsevier Ltd. Surface self‐healing: (b) The structure of the dielectric layer and self‐healing mechanism of the capacitive pressure sensor. Reproduced with permission [140]. Copyright 2024, Wiley‐VCH. (c) The self‐healing mechanism of DTG hydrogels. Reproduced with permission [141]. Copyright 2021, American Chemical Society. (d) The multiple hydrogen bond of PACG‐M. Reproduced with permission [142]. Copyright 2021, Elsevier Ltd. Interface self‐healing: (e) The self‐healing and alignment mechanism of incompatible multi‐layer elastomers. Adapted with permission [145]. Copyright 2023, American Association for the Advancement of Science.

Self‐healing systems are generally categorized as extrinsic and intrinsic. Extrinsic systems rely on microcapsules containing healing agents that rupture upon damage to fill cracks, but this process is typically irreversible and limited to a single repair event. In contrast, intrinsic self‐healing arises from reversible chemical networks that allow dynamic bond dissociation and reformation under external stimuli. Representative mechanisms include dynamic covalent bonds, metal–ligand coordination, hydrogen bonding, ionic interactions, and π–π stacking (Figure 10a) [82]. This classification clarifies how different healing mechanisms contribute to mechanical recovery, interfacial reconstruction, and electrical stability. Dynamic covalent reactions, such as imine exchange, Diels‐Alder cycloaddition, amidation, olefin metathesis, and thioester exchange, allow reversible bond rearrangement and facilitate interfacial bonding between polymers or surface‐modified functional materials. Similarly, metal coordination bonds and hydrogen‐bond networks provide dynamic, reversible interactions that can dissociate and reform in response to environmental stimuli. Under external energy input, these dynamic bonds reversibly break and reform at crack interfaces. This process converts external energy into bond reorganization and interfacial reconstruction, thereby enabling efficient self‐healing [105, 137, 138]. Owing to this intrinsic dynamic reversibility, such materials exhibit greater long‐term reliability and application potential in flexible electronics.

Either a single or multiple dynamic mechanisms can achieve the self‐healing of materials and enhance the stability of flexible mechanical devices. A disulfide bond‐containing double‐network fabricated via direct ink writing (DIW) was employed as a collagen‐fiber‐inspired framework, which was subsequently combined with a self‐healable ionogel elastomer. Upon co‐curing, strong covalent bonding forms at the interface, while dynamic disulfide exchange enables autonomous self‐healing. The resulting composite exhibits high mechanical strength and electrical conductivity, making it well suited for wearable strain‐sensing applications [139]. The covalent interface and dynamic disulfide exchange jointly help maintain conductive continuity during repeated stretching, which is beneficial for reducing hysteresis and cycle‐to‐cycle signal variation. As shown in Figure 10b, the natural dynamic covalent poly(disulfide) was used as the dielectric layer of the flexible capacitive pressure sensor. Modified with lipoic acid and sprioglycol, this material contains hydrogen bonds and iron‐phenolic coordination bonds, which enable the material to achieve intrinsic self‐healing at room temperature [140]. PEGDA, methyl methacrylate sulfonate (SBMA), and PDA were polymerized and cross‐linked to prepare conductive dopamine‐triggered gelation (DTG) hydrogels, which contain hydrogen bonds, cation‐π interactions, and aromatic interactions, as shown in Figure 10c. These hydrogels can achieve self‐healing and can be applied in strain sensors [141]. The sensor fabricated by poly(acrylamide‐acrylic acid)/chitosan/MXene hydrogel (PACG‐M) exhibits self‐healing properties due to the multiple hydrogen bonding interactions as shown in Figure 10d [142]. The α‐thioctic acid was ring‐opened to construct macromolecular chains containing dynamic disulfide linkages, into which butyl acrylate was introduced to obtain a self‐healable polymer network featuring both dynamic disulfide bonds and hydrogen‐bonding interactions. After surface hydroxylation, the polymer was bonded with the EGaIn, yielding a flexible sensor with overall self‐healing capability [143]. This integrated self‐healing design is especially valuable for LM sensors, because both the conductive phase and the surrounding polymer interface must recover to prevent permanent resistance drift after damage. Carboxylic CNTs (C‐CNTs) were incorporated into a polyacrylamide (PAM)/carboxylated cellulose nanofiber (CCNF)/phytic acid (PA) system to construct a conductive composite hydrogel (MCTP). The CCNF network provides excellent dispersibility, enabling uniform distribution of C‐CNTs within the porous MCTP matrix, while PA introduces abundant hydrogen bonds and acts as a toughening agent. The dynamic hydrogen‐bonding interactions, together with amide and phosphate functional groups in the composite, endow the resulting strain sensor with both self‐adhesive and self‐healing capabilities [144]. In addition to the self‐healing within a single material of flexible electronic devices, simultaneous self‐healing between different elastomers and precise alignment can be achieved, enabling the synchronous self‐healing and self‐aligning of multi‐layer flexible mechanical devices. This ensures the stability and reliable application of the complex design and manufacturing of the devices. Multi‐layer self‐healing systems can be constructed by stacking polymers with thermodynamically incompatible backbones but identical dynamic bonding motifs, as shown in Figure 10e. In this design, methylphenylurea (MPU) and isocyanurate (IU) form dual urea bonds that serve as a common self‐healing mechanism within PDMS and poly(propylene glycol). Although the two polymers are thermodynamically incompatible, they exhibit an overlapping modulus crossover temperature range. At high temperatures, the dynamic urea bonds drive interfacial self‐healing while restricting excessive polymer chain diffusion across the interface [145]. Overall, self‐healing strategies improve long‐term reliability by repairing local mechanical damage and restoring electrical pathways, but excessive dynamic bonding may reduce mechanical strength, slow response speed, or increase hysteresis. Therefore, healing efficiency should be balanced with sensing accuracy and fatigue resistance.

### 4.3 Composite Functional Materials

Flexible electronic mechanical devices are usually composed of multi‐layer sensing layers, electrode layers, and substrates. A single material design has limitations in addressing the surface interface issues of the devices. Combining different materials with different properties can optimize the surface and interfacial performance of the devices.

**FIGURE 11:**
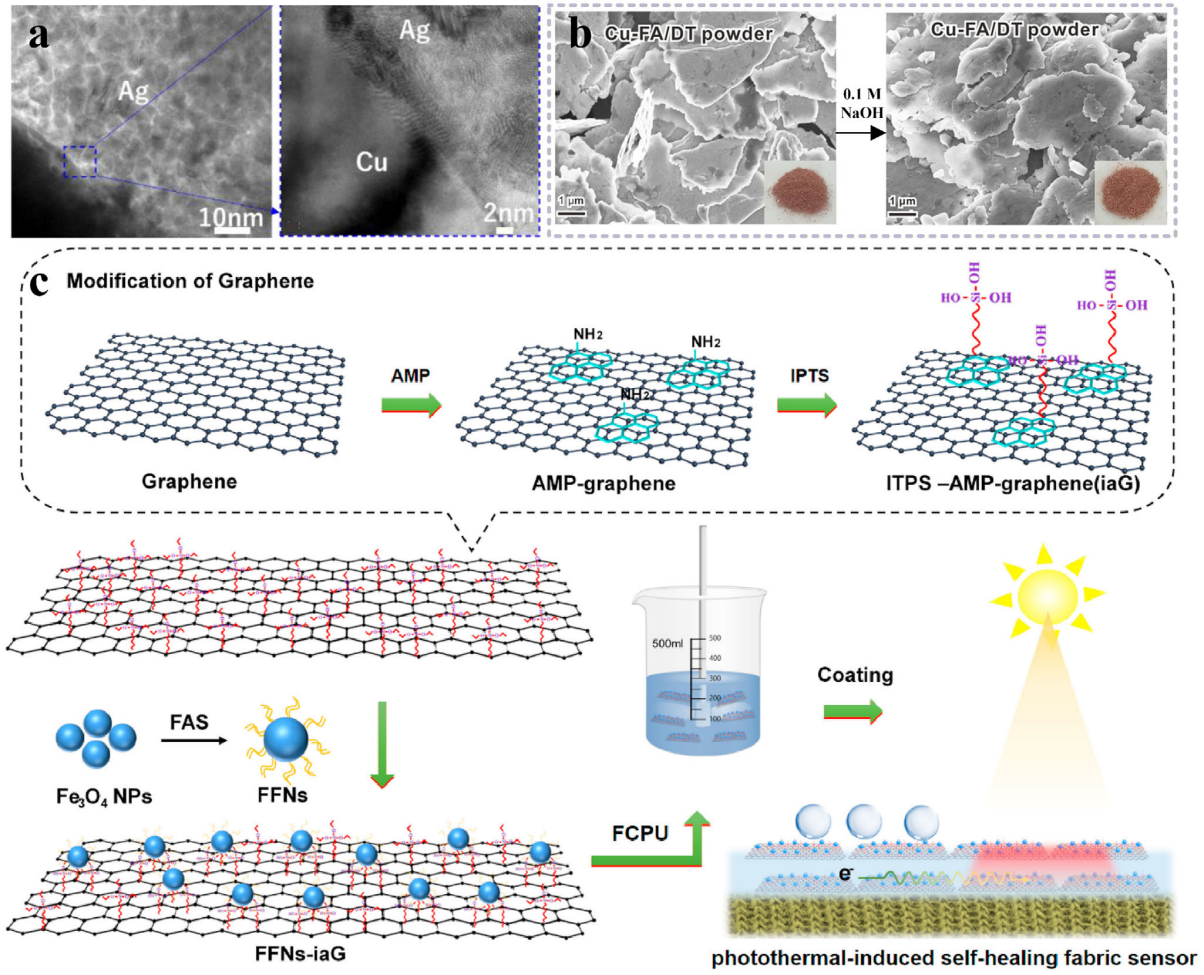
(a) Transmission electron microscopy (TEM) image of the interface in the sintered Cu–Ag composite. Reproduced under the terms of the Creative Commons Attribution 4.0 International License [148]. Copyright 2023, Elsevier Ltd. (b) SEM image of the copper microflakes passivated with formate ions and thiols. Reproduced under the terms of the Creative Commons Attribution 4.0 International License [150]. Copyright 2022, Springer Nature. (c) Synthesis of the FCPU/graphene composite coating and its hydrophobic property. Reproduced with permission [85]. Copyright 2023, Elsevier Ltd.

Conductive materials are indispensable components in electronic devices. Excellent conductors such as AgNPs and gold nanoparticles are costly, while copper nanomaterials are prone to oxidation. The surface of conductive copper nanoparticles (CuNPs) was coated and modified to endow them with antioxidant properties, thereby delaying the oxidation aging process when they were incorporated into the composite conductive sensor. The CuNPs were coated on the surface of the styrenedivinylbenzene copolymer (PS‐DVB) to form a core‐shell structure. Then, the resin was used as the matrix to prepare anisotropic conductive adhesives (ACA) [146]. The hydrothermal method was used to in situ coat graphite carbon on the surface of CuNPs, serving as the filler for the conductive adhesive of the sensor [147]. The Cu‐Ag paste prepared by sintering forms a bonding interface. The surface of Cu has no oxide layer (Figure 11a). The composite conductive material is low in cost and effectively prevents oxidation [148, 149]. The copper microflakes were passivated with formate ions and thiols (Figure 11b), which enhanced the corrosion and oxidation resistance of the copper paste and endowed the copper paste with high printing suitability [150]. For conductive adhesives and printed electrodes, oxidation‐resistant fillers help stabilize electrical transport and reduce resistance drift during long‐term exposure to air, humidity, and thermal cycling.

After achieving intrinsic stability of the functional materials, flexible electromechanical devices must still maintain reliable operation under practical environmental conditions, such as humidity, corrosion, and UV irradiation. The composite design actively endows the surface with protective properties by incorporating low surface energy components and functional fillers. For instance, by combining fluorinated comb‐shaped PU (FCPU) with graphene through perfluorooctyltriethoxysilane, an ultra‐hydrophobic conductive coating is fabricated, as shown in Figure 11c. The contact angles of this coating with water, milk, coffee, sauce, acidic and alkaline liquids are all greater than 150° [85]. Due to the rough porous surface structure of the aerogel, it inherently possesses hydrophobic properties. By modifying the composite conductive reduced graphene oxide@polyacrylonitrile nanofiber/CNTs aerogel with octadecylamine, which is environmentally friendly and hydrophobic, a pressure sensor was prepared. This sensor has a high oil/water separation ability, with a water contact angle of up to 154° and a rolling angle of only 6° [151]. A sensor was fabricated by dispersing PDA‐Ga into carbon black (CB)‐filled PDMS. After curing, the presence of nanoscale Ga imparted robust antibacterial activity. By further introducing a bioinspired lotus‐leaf microstructured surface together with the intrinsically low surface energy of PDMS, the sensor also became hydrophobic, exhibiting a static water contact angle of 121.7° and a low sliding angle of 25° [86]. The strain sensor prepared with the composite material of PVA/sodium lignosulfonate (LS)/LiCl/EG has excellent UV resistance, because the unsaturated groups in lignin absorb the energy of the incident UV radiation in the ground state [93]. MXene was incorporated into a hydrogel network composed of phenylboronic acid‐grafted hyaluronic acid and TA, where interfacial supramolecular interactions endowed the composite with excellent dispersion stability. Moreover, this material exhibited good biocompatibility, water retention capacity, biodegradability, cell adhesion ability, and excellent conformal mechanical properties. The phenol and ketone groups in TA have UV light absorption ability. The MXene nanosheets can undergo interband transitions during UV light absorption [94]. These protective composite designs improve device‐level stability by reducing humidity‐induced swelling, UV‐induced degradation, and contamination‐related signal fluctuation. Their limitation is that additional fillers or coatings may increase stiffness or weaken mechanical compliance if not properly optimized.

Based on the stability and tolerance of mechanically coupled flexible electronics, the optimized interface needs to achieve a firm bond with biological tissues or heterogeneous substrates, and maintain conformal contact and dynamic adaptability. The optimization of the interface can be achieved by constructing an extensive dynamic interaction network. The sensor made of PVA/CNF@PDA/CNT composite material, due to the abundant catechol groups provided by PDA, has led to the formation of a large number of non‐covalent interactions both within the device and with the conformal substrate, resulting in excellent surface adhesion strength [152]. By incorporating biocompatible supramolecular solvents (SMS) into PEDOT:PSS and PVA, a self‐adhesive conductive polymer (SACP) was prepared. This material possesses both the conductivity and flexibility of polymers, and the hydroxyl groups and weak interactions of the SMS have an adhesion strength greater than 30 N m^−1^ with various films [153]. The simultaneous improvement in adhesion and conductivity is important for wearable devices, because it can reduce motion artifacts caused by local detachment while maintaining stable electrical readout. Controlling the size of the ILs to adjust the layer spacing of MXene can prevent its self‐aggregation and mix it into the PAA‐PDA@SiO_2_ hydrogel. The fabricated flexible sensor exhibits excellent surface adhesion [154]. The interfacial synergy between MXene and liquid metals is critical. MXene stabilizes liquid‐metal droplets, enhances interfacial contact with hydrogels, and builds hybrid conductive pathways. Liquid metals contribute deformable conductivity, MXene promotes interfacial anchoring and electron transport, and the hydrogel matrix provides stretchability and adhesion. This cooperative design reduces conductive‐network fracture, resistance drift, and hysteresis under repeated deformation [155]. A shape memory polymer was integrated with poly(stearyl acrylate)/dodecyl methacrylate, and the composite was incorporated into a skin patch. Through the combined regulation of temperature and water vapor, the system provides strong skin adhesion and excellent adaptability [156]. In summary, composite functional materials can simultaneously improve environmental tolerance, interfacial adhesion, and conductive stability, thereby reducing motion artifacts, signal drift, and output instability under practical operating conditions. However, the interactions among fillers, matrices, and substrates must be carefully controlled to avoid aggregation, stiffness mismatch, and reduced reproducibility.

### 4.4 Novel Flexible Substrates

Some flexible sensors based on non‐polymer elastomers, such as paper and fabric, inherently possess the capabilities of bending, folding, and stretching. Moreover, paper and natural fabrics have microscopic or macroscopic porous characteristics. When liquid functional materials are deposited on them, capillary action will spontaneously drive the materials to infiltrate the internal pores of the base, ensuring that the materials penetrate the three‐dimensional network inside the base. After solidification, the functional materials are firmly anchored in the three‐dimensional fiber network or fabric network structure, achieving mechanical interlocking, which significantly enhances the interface bonding strength between the functional materials and the substrates. This effect helps reduce material peeling and conductive‐layer displacement during bending, folding, or stretching, which is beneficial for lowering hysteresis and improving cyclic signal stability.

**FIGURE 12:**
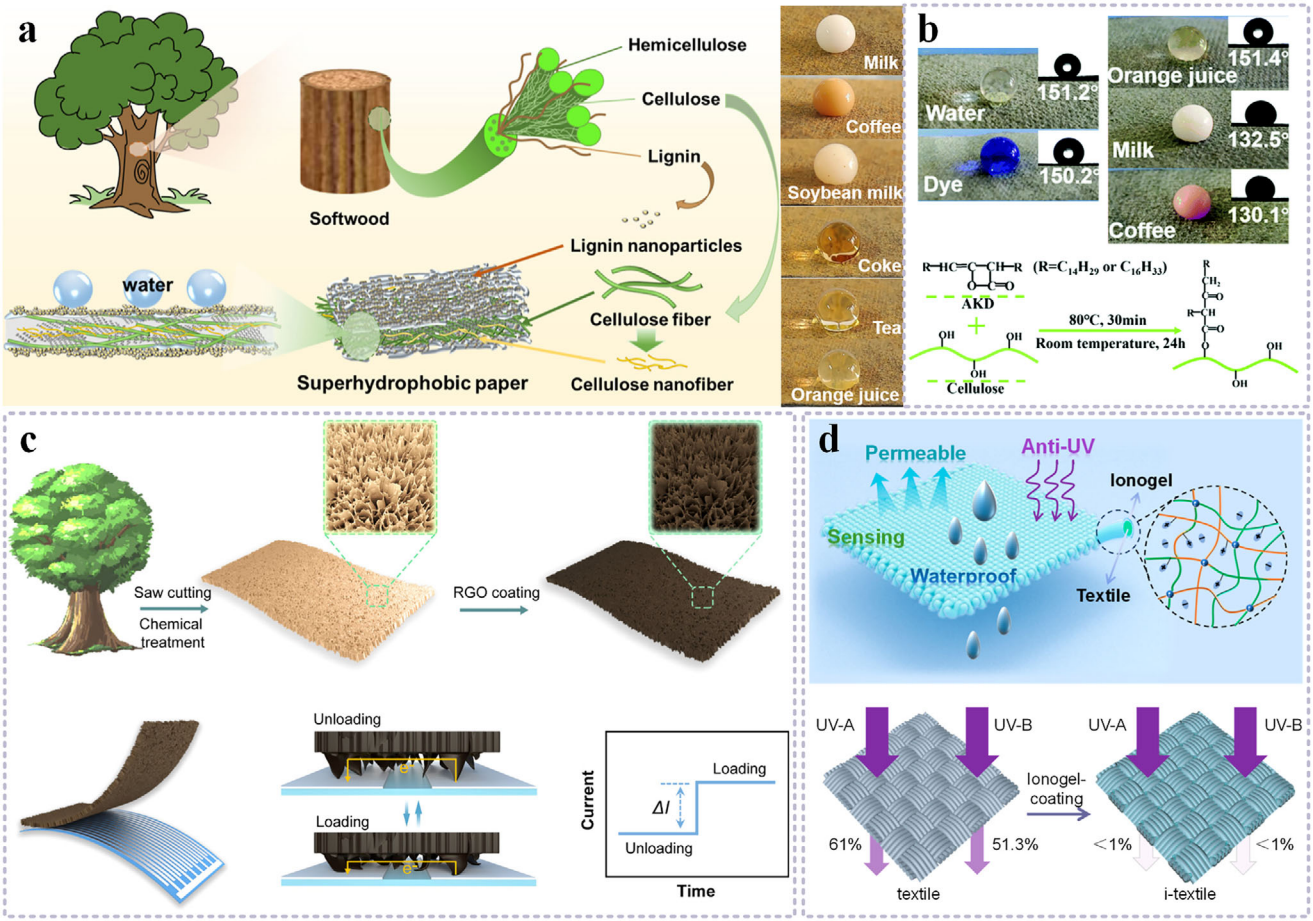
Novel Flexible Base Material Sensors: (a) Manufacturing process and surface hydrophobicity of the paper‐based sensor coated with PDMS. Reproduced with permission [84]. Copyright 2023, American Chemical Society. (b) Surface hydrophobicity of the paper‐based sensor coated with AKD. Adapted with permission [83]. Copyright 2022, Royal Society of Chemistry. (c) Wooden‐based pressure sensor. Reproduced with permission [159]. Copyright 2020, American Chemical Society. (d) UV absorption capacity of the i‐textile. Adapted with permission [160]. Copyright 2024, American Chemical Society.

Lignin intrinsically possesses a certain degree of hydrophobicity. By integrating papermaking techniques, cellulose nanofibers (CNF) extracted from wood were combined with graphene to fabricate a composite paper‐based sensor. Through controlled high‐temperature treatment and PDMS spray coating, targeted superhydrophobicity was achieved. As shown in Figure 12a, droplets of different liquids all exhibit stable hydrophobic contact angles on the surface of the paper‐based sensor [84]. The paper‐based sensor made by mixing CNF with graphene can also achieve hydrophobicity by coating with alkyl ketene dimer (AKD) emulsion, and the hydrophobic contact angles with different liquids are shown in Figure 12b [83]. Commercial ceramic fiber paper (CFP) possesses thermal stability. When it is made into a cracked structure, after sputtering AuNPs and then spin‐coating SiO_2_ aerogel, the coating exhibits both hydrophobicity and high‐temperature resistance, with a WCA of up to 159°. The crack sensor can operate stably at a temperature of 220°C [157]. Multi‐layer fibrous paper was constructed by alternately stacking flat and corrugated sheets, followed by coating with two‐dimensional SnS to fabricate a pressure sensor. The multi‐layer paper together with the puckered lattice structure of SnS, synergistically enhances the sensing performance, while the SnS film simultaneously imparts surface hydrophobicity [158].

The rough‐surfaced wood is directly sliced and chemically treated with hemicelluloses to form a sustainable wood‐based flexible substrate. During the wood cutting process, it has a microstructure that can provide high sensitivity for flexible pressure sensors (Figure 12c), and the presence of the structure and the surface properties of the wood enable the strong conductivity of reduced graphene oxide (rGO) after coating [159].

Textiles possess an intrinsically porous structure, allowing functional materials to penetrate into the interior of fibers and disperse within the interwoven fabric network. This configuration not only increases sensing sites by leveraging the three‐dimensional porous structure of the textile, thereby enhancing sensing performance, but also reduces material detachment and interfacial instability. A kind of ionically conductive polymer was prepared by using butyl acrylate, poly(urethane acrylate) (PUA), and PEGDA for crosslinking. 1‐hydroxycyclohexyl phenylketone was used as a photoinitiator, and (1‐ethyl‐3‐methylimidazole bistrifluoromethionimide ([EMIm] [TFSI]) was added as an IL, and the mixture was immersed in ionic textile (i‐textile). The excellent hydrophobic property of PUA ensures that the waterproofing ability of the fabric [EMIm] [TFSI] firmly wraps around the fabrics, as shown in Figure 12d. The ketones, conjugated bonds, and imidazole groups in the iontronic polymer enhance the UV absorption of the i‐textile, making it much greater than that of ordinary textiles [160]. Although paper, wood, and textile substrates offer porosity, flexibility, and sustainable features, their long‐term reliability still depends on moisture resistance, fiber fatigue, and stable bonding between the porous matrix and conductive materials.

## 5 Chemical‐Driven Strategies for Surface and Interfacial Enhancement

Material design and modification can address various surface‐related issues in flexible devices and enable surface functionalization for multi‐layer integration, as these strategies originate from the intrinsic design of the material itself. However, chemical‐driven strategies provide more direct routes to change the surface chemistry characteristics of flexible substrates or functional films, for example by introducing functional groups, tuning surface energy, or creating additional reactive sites. Furthermore, interfacial chemical engineering enables the rational design of chemical structures and reaction pathways at the interface between different material layers, allowing interfacial grafting or covalent bonding to strengthen multilayer adhesion. These strategies effectively suppress interfacial delamination, reduce interfacial energy dissipation in mechanical sensors, stabilize conductive networks, and improve electrode signal stability in flexible devices.

### 5.1 Surface Chemical Grafting and Adsorption

Through chemical modification, the properties of the material surface can be altered, including those of composites, elastomers, sensing materials, and electrode materials. Common surface modification methods include surface functionalization, surface graft polymerization, coupling agent grafting, and surface chemical adsorption. Table 1 summarizes representative surface modification strategies for flexible materials, highlighting their key characteristics and corresponding typical materials.

**TABLE 1:** Classification of surface chemical modification strategies, representative materials, and their effects in surface engineering.

Classification of surface chemical modification strategies		Characteristics	Typical materials	Effects
Covalent Bond Modification	Surface Grafting Polymerization	Polymer chain grafting to form brush‐like or dendritic surface structures	Polymer grafting on metal surfaces	Enhanced surface wettability and adhesion
			Grafting hydrophilic polymer chains onto low‐surface‐energy polymers	Improved polymer hydrophilicity
			Grafting polyethyleneimine (PEI) or PVA onto AuNPs/AgNPs surfaces	Altered surface charge; improved dispersibility
	Coupling Agent Grafting	Coupling‐agent‐induced covalent bonding	Silane coupling for metal‐polymer interfaces	Improved interfacial adhesion
	Surface Functionalization	Conversion of native surface groups via chemical reactions	Oxidation and doping of graphene, CNTs, carbon fibers	Enhanced hydrophilicity; introduced reactive sites; improved covalent bonding; tunable conductivity
			Oxidation/reduction and coordination of metals and metal oxides	Improved compatibility; enhanced chemical stability; tunable conductivity
			Surface modification and oxidation of PDMS, PU, Ecoflex	Enhanced hydrophilicity; introduced reactive sites; improved adhesion
			Secondary doping of PEDOT:PSS	Enhanced conductivity; improved mechanical properties; strengthened interfacial bonding
Non‐covalent Adsorption	Electrostatic Adsorption	Electrostatic adsorption of oppositely charged molecules or ions onto surfaces	Surface adsorption on AuNPs/AgNPs	Enhanced surface conductivity; target molecule adsorption; charge density regulation; improved mechanical properties
			polyaniline (PANI), polypyrrole (PPy), PEDOT:PSS	
			PAAm, PEI	
	Van der Waals Adsorption	Surface interaction via weak van der Waals forces	Graphene, CNT, nanoparticles	Improved surface mechanical strength and conductivity; surface functionalization
	Hydrogen Bond Adsorption	Hydrogen‐bond‐driven molecular adsorption for surface modification	PU, hydrogel	Tunable wettability for self‐cleaning and antibacterial properties

Surface covalent modification immobilizes molecules on material surfaces through covalent bonding, providing strong and irreversible attachment that enables the long‐term stabilization of surface functional groups or molecules, thereby addressing surface‐related challenges in flexible electronic devices. Surface graft polymerization is the most common method for surface modification, including grafting‐to and grafting‐from. The former involves the pre‐synthesized polymer chains being chemically attached to the surface, while the latter involves the growth of polymer chains on the surface through a polymerization reaction. They can all modify the surface of materials that originally have low surface energy, poor hydrophobicity, or poor dispersibility, to achieve surface hydrophilicity, adhesion, and excellent electrical properties. For example, through Norrish II photochemical hydrogen abstraction, isopropyl thioxanthone semi‐pinacol (ITXSP) dormant groups were covalently introduced onto the surface of low‐density polyethylene (LDPE). Subsequent visible‐light‐initiated graft crosslinking of PEGDA constructed a PEG network on the PE surface, within which PEDOT:PSS was in situ embedded. This surface photo‐initiated graft crosslinking strategy enables the fabrication of conductive layers on flexible sensor surfaces [161]. Under acid catalysis, Si–H groups from poly(methylhydrosiloxane) (PHMS) were introduced onto the PDMS surface, followed by grafting of PEG methyl ether acrylate. This modification enabled PDMS to maintain a low surface WCA for an extended period, while increasing the surface energy by approximately 20% [162]. It is also possible to perform grafting with coupling agents on the surface of the materials, especially by grafting silane coupling agents onto the surfaces of various polymer elastomers, thereby enhancing the adhesion to other materials. For example, PET was immersed in a 10% (3‐mercaptopropyl)triethoxysilane (MPTES) hydrolysis solution, activating the surface of PET to form a silane monolayer. CuNPs were deposited on it using inkjet printing technology and then cured. The bonding of Cu with the thiol group significantly enhanced the interface bonding ability between the electrode and the substrate [163].

Surface functionalization introduces reactive sites and specific functional groups onto material surfaces through chemical reactions. As one of the most widely used surface chemical strategies, it enables functional modification of flexible electronic devices, enhancing adhesion between metals and polymers, coatings, or adhesives, while improving corrosion resistance, oxidation stability, self‐cleaning capability, and tunable electrical properties. The surface of PMMA microcolumn structures coated with Ag was chemically modified with perfluorooctanoic acid or Drysurf solution, which resulted in a two‐sided hydrophobic surface and excellent self‐cleaning ability [164]. The proportions of vinyl‐multifunctional PDMS, hydride‐terminated PDMS, silanol‐terminated PDMS and resin composite materials were optimized to prepare adhesives. The functional group modification can change the bonding strength and material modulus [165]. However, surface functionalization must be carefully optimized, because excessive chemical treatment may damage soft substrates, alter modulus, or introduce brittle surface layers that accelerate crack initiation.

**FIGURE 13:**
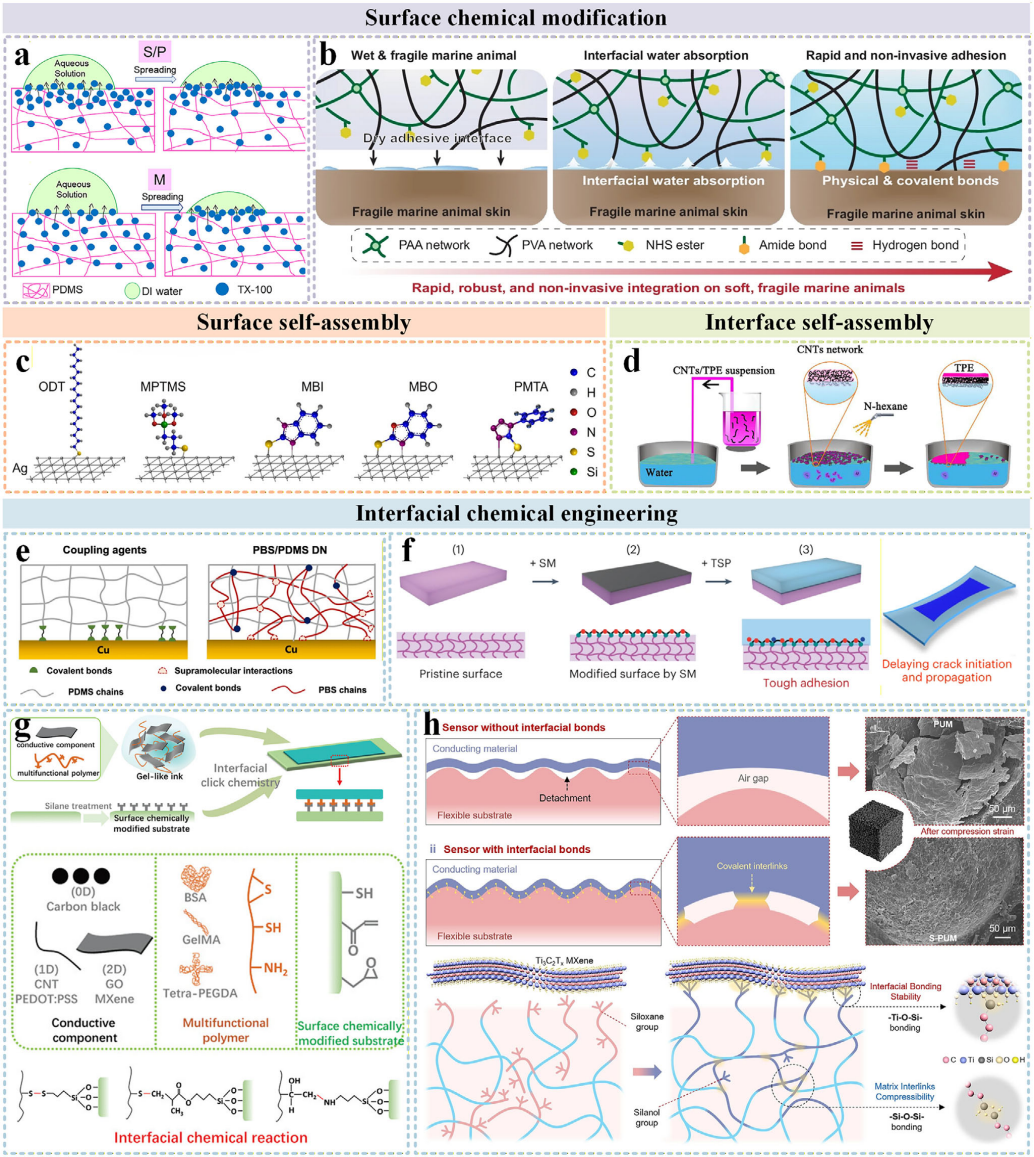
Surface chemical modification: (a) Hydrophilic mechanisms of different PDMS processing methods by TX‐100. Reproduced with permission [168]. Copyright 2020, Elsevier Ltd. (b) Adhesion mechanism of BIMS. Reproduced under the terms of the Creative Commons Attribution 4.0 International License [169]. Copyright 2024, Springer Nature. Surface self‐assembly: (c) Modification of different SAMs on AgNWs. Reproduced with permission [172]. Copyright 2023, Elsevier Ltd. Interface self‐assembly: (d) Fabrication process of a TPE/CNTs superhydrophobic sensor via air‐water interfacial self‐assembly. Reproduced with permission [176]. Copyright 2021, Elsevier Ltd. Interfacial chemical engineering: (e) Interfacial bonding mechanism of DN and Cu. Reproduced with permission [179]. Copyright 2023, Elsevier Ltd. (f) Dynamic chemical bonding interface to prevent crack propagation. Reproduced with permission [18]. Copyright 2022, Springer Nature. (g) General click chemistry strategy to enhance sensor interfacial adhesion. Reproduced with permission [60]. Copyright 2023, Wiley‐VCH. (h) Interfacial bonding mechanism of Ti–O–Si bond to enhance modification of PU and MXene. Reproduced with permission [180]. Copyright 2024, Elsevier Ltd.

Without forming chemical bonds, molecules or small molecules can also adhere to the surface of the material through non‐covalent interactions such as van der Waals forces and electrostatic forces. This can temporarily adjust the surface properties. The adhesion force is relatively weak and is prone to detachment under changes in external conditions. However, no chemical reactions are required, making the preparation process simpler and more efficient. In addition, during the preparation stage, surfactants can be added to the silicone rubber before it is cured. For example, Triton X‐100 (TX‐100) can be adsorbed onto the PDMS surface. Because the hydrophilic groups in TX‐100 diffuse and accumulate on the PDMS surface when water is present on it, the WCA on the PDMS surface gradually decreases over time. Moreover, this non‐covalent adsorption effect is reversible [166, 167]. However, directly modifying PDMS with TX‐100 would reduce the transparency of the film. When TX‐100 is swollen in toluene and then used to modify the surface of the PDMS film, as shown in Figure 13a, compared to direct mixing, TX‐100 can penetrate the surface layer of the PDMS and does not affect the mechanical properties of the PDMS, and can also maintain the surface hydrophilicity [168]. Compared with covalent grafting, non‐covalent adsorption offers simpler and more reversible surface regulation, but weaker bonding may lead to gradual functional loss and unstable signal output under washing, sweating, or repeated contact.

In practice, the most commonly employed strategy involves combining multiple surface modification approaches, where covalent bonds and diverse non‐covalent interactions jointly address surface‐related challenges in flexible electronic devices. A film composed of two networks interpenetrating each other, namely PVA and PAA grafted with N‐hydroxysuccinimide (PAA‐NHS) ester, was designed for the bioadhesive interface of marine sensors (BIMS), as shown in Figure 13b. The PAA‐NHS ester has amide covalent bonds and multiple non‐covalent interactions with the marine biological epidermis, forming a stable adhesion while also having hygroscopic ability and excellent mechanical properties. The fabricated sensors can stably adhere to various fish skin surfaces in water. Furthermore, the swollen adhesive can also ensure the energy dissipation of the sensor when the skin deforms, thereby preventing the failure of the device [169]. By in situ polymerization of pyrrole on the hydroxyl groups of silk/cellulose composites, strain sensors exhibiting both high electrical conductivity and strong mechanical properties can be obtained. The multiple covalent bonds present contribute to enhanced surface stability and wear resistance, supporting their durability in practical applications [170]. In summary, surface chemical grafting and adsorption are the most common methods for addressing surface issues in mechanically coupled flexible electronics. Different treatment approaches and effects are employed for metals, polymers, and nanomaterials. However, their practical use still requires attention to treatment durability, process reproducibility, and compatibility with soft substrates.

### 5.2 Molecular Self‐Assembly at Surfaces and Interfaces

Self‐assembled monolayers (SAMs) involve the spontaneous, ordered formation of a single molecular or nanoscale layer on flexible substrates. Anchored via interactions with specific substrate functional groups, SAMs enable precise surface modification, imparting hydrophilicity, hydrophobicity, biocompatibility, corrosion resistance, or conductivity, and have been applied in surface engineering of flexible mechanical sensors. Polyethersulfone (PES) was selected as the flexible substrate, and the deposited Al_2_O_3_ layer was modified via SAMs to enhance adsorption of GO nanosheets and their subsequent reduction to rGO. The resulting flexible electronic devices are capable of detecting human physical movements [171]. As shown in Figure 13c, when different SAMs with various chemical functional groups, including 3‐mercaptopropyl trimethoxysilane (MPTMS), octadecanethiol (ODT), 2‐mercaptobenzoxazole (MBO), 2‐mercaptobenzimidazole (MBI), and 1‐phenyl‐5‐mercaptotetrazole (PMTA) are used to modify the PDMS layer, they have different electronic transfer capabilities for silver nanowires (AgNWs). Among them, the S in MPTMS achieves a close bond with Ag, resulting in a higher potential output performance of the fabricated triboelectric nanogenerator (TENG) and improved pressure sensing performance. The improved charge transfer and interfacial bonding can reduce output fluctuation in triboelectric and pressure sensors [172]. The concentration and temperature of MPTMS have certain effects on its degree of hydrolysis. The optimal hydrolysis conditions, combined with the liquid‐phase deposition (LPD) process, enable MPTMS to be deposited on the surface of PDMS, thereby significantly enhancing the adsorption capacity between Au and PDMS [173]. Plasma‐treated PI films were modified with MPTMS SAMs, followed by inkjet printing of Cu complex ion ink. The formed Cu–S bonds enhance electrode‐substrate interface adhesion, and a post‐sintering MPTMS coating provides oxidation resistance and hydrophobic self‐cleaning functionality [174]. After the PET film is soaked in NaOH solution, it is immersed in the hydrolysis solution of (3‐Aminopropyl)triethoxysilane (APTES) and MPTES to form SAMs. Then, Ag ink and copper electrodes are printed by inkjet printing. The mercapto groups and amino groups on the surface of the SAMs react with the metals to form stable interface bonds, reducing the coffee ring effect in inkjet printing and helping produce more uniform electrodes, which is beneficial for reducing device‐to‐device variation and unstable resistance response [175].

Interfacial self‐assembly involves the spontaneous formation of two‐dimensional films at gas‐liquid or liquid‐liquid interfaces. It also relies on non‐covalent interactions between molecules and the interaction between hydrophilic head groups and water to form a highly ordered film. As shown in Figure 13d, the thermoplastic elastomer (TPE)/CNTs composite dispersion is pumped into water, forming a TPE/CNTs sheet at the air/water interface. A hexane spray dissolves the TPE segments, and after curing, a stable TPE/CNTs film is formed. By taking advantage of the rapid evaporation property of hexane, rough surface structure superhydrophobic strain sensors can be fabricated [176]. The self‐assembly at the air/water interface can also form a TPE/MWCNTs/PDMS superhydrophobic film. PDMS can bond with MWCNTs while maintaining the integrity of the micro‐topological structure, and its low surface energy further enhances the superhydrophobic property [177]. The PDMS solution was self‐assembled into a film through the air/water interface. After the water evaporated, the film was subjected to bending treatment to obtain a microstructure with a fish‐scale‐like surface. This film, as a pressure sensor film, has superhydrophobic properties [178]. Overall, these self‐assembled films are effective for constructing hydrophobic and conductive surfaces, but their long‐term stability may be limited by molecular desorption, surface contamination, and mechanical wear during repeated use.

### 5.3 Interfacial Chemical Crosslinking and Grafting

The interfacial chemical‐driven strategies for multi‐layer mechanically coupled flexible electronics mainly rely on chemical methods to increase the interfacial bonding force, enhance the interfacial toughness, and prevent the interfacial failure. Introducing a polymer interfacial layer with dynamically reversible bonds between the functional layer and the substrate can dissipate energy through the breaking and reformation of the bonds. For example, PDMS‐OH is cross‐linked with boronic acid to form a polyborosilane (PBS) network. Incorporating PBS into the PDMS pre‐polymer enables good penetration and curing of PDMS, but provides anchoring of dynamic borate bonds and non‐covalent bonds on the PDMS surface. As shown in Figure 13e, depositing Cu on the surface of this double network (DN) film significantly enhances the interfacial bonding strength through supramolecular interactions [179]. A thermal interfacial material is introduced between the semiconductor film and PDMS. This interfacial layer, a tough self‐healing polymer matrix (TSP), contains dynamic chemical bonds. A surface modifier is incorporated into the TSP, covalently bonding to both the semiconductor film and PDMS. Owing to the presence of strong and weak hydrogen bonds in the TSP, it can effectively dissipate mechanical energy, suppress delamination, and delay crack initiation and propagation (Figure 13f). Meanwhile, this energy‐dissipating interface helps preserve signal stability by preventing progressive interfacial damage during bending, stretching, and thermal cycling [18]. It is also possible to achieve in situ and click chemical bonding at the interface between the functional layer and the substrate by employing the chemical and covalent bond grafting strategy. As shown in Figure 13g, a universal interface click chemistry strategy involves using multifunctional polymers with reactive groups, such as gelatin methacryloyl (GelMA) or PEGDA, as additives in the sensing layer. Elastomeric sensor substrates, such as latex, PDMS, Ecoflex or TPU, are surface‐modified with thiol, vinyl, or epoxy groups. The formation of stable chemical bonds at the interface significantly enhances the adhesion and durability of the resulting crack‐based strain sensors [60]. γ‐methylacryloxypropyl triethoxysilane (γ‐MPS) was copolymerized with PUA to introduce trimethoxysilane (─Si(OCH_3_)_3_) groups, yielding silane‐modified PU (S‐PU). Condensation with hydroxyl‐rich MXene forms robust Ti─O─Si bonds at the interface, providing pressure sensors with strong adhesion, long‐term stability, and enhanced resistance to friction, acids, and environmental stress (Figure 13h) [180]. By initiating polymerization reactions on the substrate surface to graft functional polymer brushes or molecular layers, a strong interfacial bonding and functionalized surface can be constructed, providing a foundation for depositing or anchoring conductive materials. A strain sensor was fabricated on a silicone rubber (SR) substrate using a graphene nanosheet (GNP)/modified poly(sodium 4‐phenylene sulfonate) (MOPSS) sensing layer with a crack structure. The silane terminal of MOPSS condenses with SR, forming covalent bonds that enhance interfacial adhesion and suppress GNP aggregation, ensuring long‐term stability and high reproducibility [181]. This molecular‐level interface is beneficial for reducing hysteresis and improving repeatable sensing, since the conductive layer remains anchored to the elastomer during cyclic deformation. Furthermore, in situ chemical reactions enable the direct growth of functional materials on the substrate surface, achieving molecular‐level integration at the interface. For instance, by depositing LM onto the surface of a Very High Bond (VHB) tape polymer film and using the hydrothermal method to grow CoS_2_ nanomaterials on its surface, in situ hydrothermal growth effectively ensures the uniform growth of the functional layer and the stability of the interface [182]. Interfacial adhesion can be enhanced via multiple mechanisms. Grafting polymer brushes on PDMS by subsurface‐initiated atom transfer radical polymerization of 2‐(2‐bromoisobutyryloxy)ethyl methacrylate, followed by electroless deposition of metal nanoparticles, forms an interface fusion layer [183]. In summary, interfacial chemical crosslinking, grafting, modification and in‐situ growth can effectively enhance the interfacial bonding strength of multi‐layer mechanically coupled flexible electronics, ensuring that no interfacial delamination or crack propagation issues occur under external forces. Nevertheless, these chemical strategies may increase processing complexity and reduce reversibility.

## 6 Physical‐Driven Strategies for Surface and Interfacial Enhancement through Processing and Manufacturing

The fabrication of flexible electronic devices relies on various physical processing techniques, including laser ablation, magnetron sputtering, ion beam deposition (IBD) processing, electron beam deposition (EBD) processing, plasma treatment, UVO treatment, photolithography, 3D printing, inkjet printing, screen printing, spin coating, spray coating, and pad printing. These techniques not only enable the construction of diverse surface architectures for structural design of electronic devices, but also facilitate the multifunctionalization of surfaces. Owing to their distinct physical principles, different physical processing techniques exert varied influences on the formation, adhesion, bonding, and damage of surfaces and interfaces. Consequently, the interfacial bonding strength and mechanisms in multi‐layer devices are strongly dependent on the selected fabrication process. Optimization or modification of physical processing techniques may further induce differential effects on surface or interfacial characteristics. In addition, interfacial bonding in multi‐layer devices or surface functionalization may rely on physical structures, and various physical processing techniques allow the controllable fabrication of structures across multiple scales, thereby achieving different surface and interfacial enhancement effects. In summary, Table 2 summarizes the characteristics of surface and interfacial enhancement strategies driven by different physical processing and manufacturing.

**TABLE 2:** Influence of different physical processing on the surface and interface of mechanically coupled flexible electronics.

Techniques	Characteristics	Surface enhancement	Interfacial advantages	Interfacial disadvantages
Laser Ablation	Local removal by high‐energy laser pulses	Micro/nanostructuring to tune surface roughness and functionality	In situ device fabrication; high‐resolution micro/nanostructures for mechanical interlocking	Thermal damage; high cost
Magnetron Sputtering	Magnetically enhanced ion bombardment for multi‐material deposition onto substrates	Film uniformity; surface functionality	Film uniformity, good interfacial adhesion; low‐temperature processing	Low deposition rate; high cost; limited enhancement of bonding strength
IBD	Ion beam sputtering for atomic evaporation and deposition onto substrates	Dense and uniform films; impurity removal; improved chemical stability	Dense and uniform film deposition; good interfacial adhesion	Low deposition rate; unsuitable for large‐scale manufacturing
EBD	Electron beam evaporation for material deposition onto substrates	Precise control over deposition rate; film uniformity; surface functionality	Film uniformity; good interfacial bonding; suitable for high‐melting‐point materials	High cost; unsuitable for large‐scale manufacturing
Evaporation Deposition	Thermal evaporation for material deposition onto substrates	Low‐temperature deposition of dense films; surface functionality	Low‐temperature processing; strong bonding between metal and substrate	Limited to depositing low‐melting‐point materials
Plasma Treatment	Activation of gas to generate charged particles and free radicals for surface reaction	Enhanced hydrophilicity and surface reactivity	Improvement in interfacial adhesion; low cost; high efficiency	Possible surface damage; temporary chemical modification
UVO Treatment	Surface oxidation via UV irradiation in an ozone‐rich environment	Organic contaminants removal; surface hydrophilicity and adhesion	Formation of oxide layers enhancing conductivity or insulation; interface stability	Long processing time; temporary chemical modification; potential over‐oxidation affecting performance
Photolithography	Pattern formation by exposing a photosensitive material to UV light through a mask	Selective surface treatment; surface functionality	Precise control over pattern dimensions; formation of interfacial structures; enhanced interfacial electrical properties and adhesion	Complex process; high cost
Nanoimprint Lithography	Nanoimprinting to transfer patterns onto material surfaces	High‐resolution surface structures; surface functionality	Increased interfacial contact area; enhanced interfacial adhesion; low‐cost	Requires high‐precision molds; complex structures involve intricate processing
Inkjet Printing	Ejection of liquid ink as droplets from a nozzle onto a substrate	Film uniformity; surface functionality	Tunable material properties; precise control over film thickness; enhanced interfacial electrical properties; low cost; high precision	Constrained by material properties; limited printing precision
Screen Printing	Transfer of ink onto a substrate using a mesh screen	Film uniformity; surface functionality	Suitable for large‐scale fabrication of complex patterns; low cost; high efficiency; wide range of applicable materials; interfacial adhesion	Constrained by material properties; low manufacturing precision
3D Printing	Layer‐by‐layer deposition of liquid or solid materials to form a 3D structure	Design and fabrication of complex surface structures; surface functionality	Fabrication of complex structures enabling interfacial electrical interconnection; high design freedom	Constrained by material properties; slow printing speed
Spin Coating	Liquid material onto a substrate, followed by high‐speed rotation for uniform coating	Rapid, thickness‐controlled surface functionalization	Rapid deposition of interfacial materials; enhanced interfacial adhesion; simple process	Unsuitable for large‐area manufacturing; potential issues with solvent evaporation and coating non‐uniformity
Spray Coating	Spraying of material onto a substrate through a nozzle	Rapid surface functionalization	Suitable for large‐area coating; interfacial adhesion based on material properties	Low precision; difficult thickness control; constrained by material properties

### 6.1 Energy‐Beam‐Based Ablation and Modification

**FIGURE 14:**
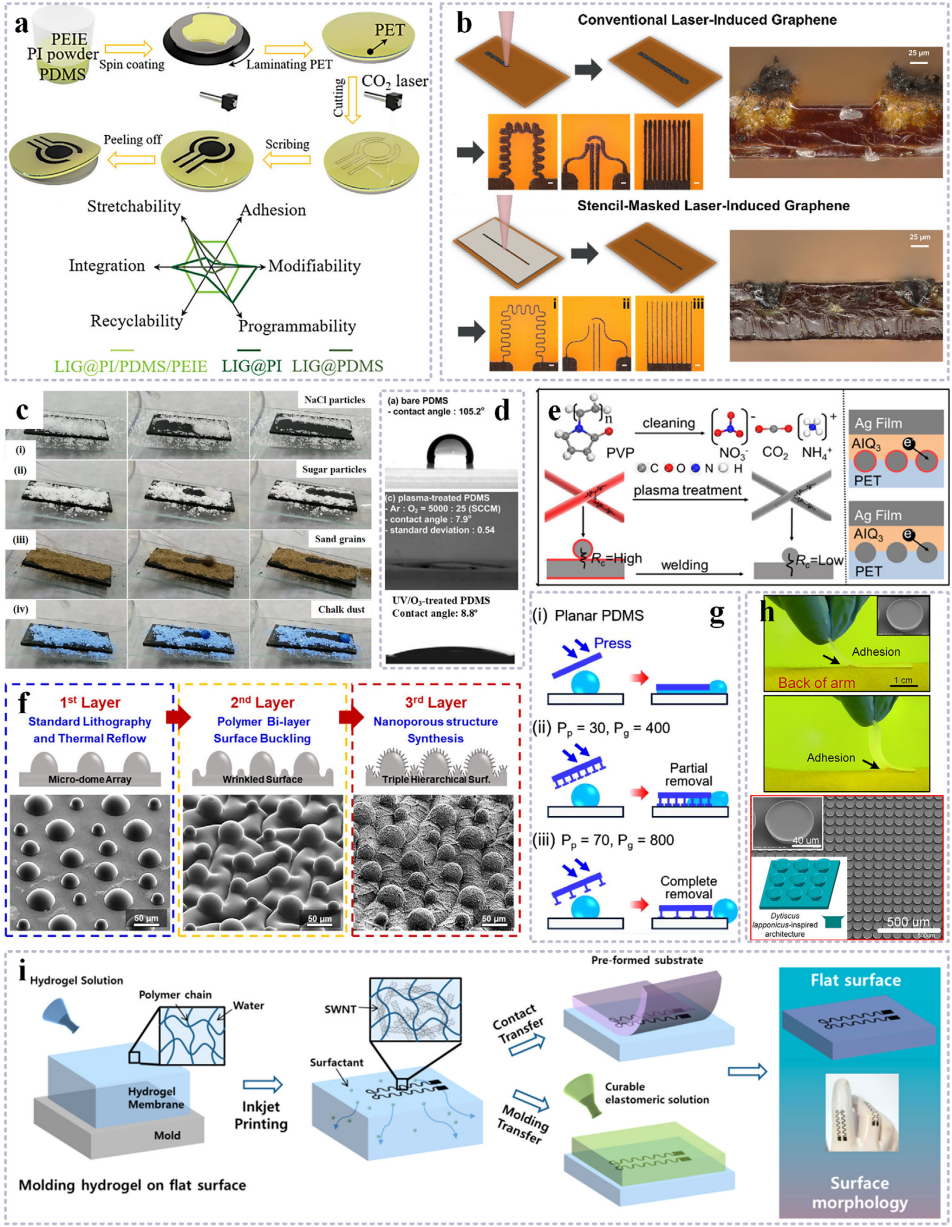
Physically‐driven strategies for surface and interfacial enhancement: (a) Fabrication process of LIG@PI/PDMS/PEIE. Reproduced under the terms of the Creative Commons Attribution 4.0 International License [186]. Copyright 2024, Wiley‐VCH. (b) Comparison of LIG and s‐LIG electrodes. Reproduced with permission [187]. Copyright 2024, Elsevier Ltd. (c) WCA and self‐cleaning capability against various particles on a sputter‐deposited Zn thin film surface. Reproduced with permission [189]. Copyright 2022, American Chemical Society. (d) WCA of PDMS surfaces without treatment, after plasma treatment, and after UVO treatment. Reproduced with permission [193, 194]. Copyright 2020, Indian Academy of Sciences. Copyright 2011, Elsevier Ltd. (e) Mechanism of plasma‐assisted welding of AgNW films and removal of PVP. Reproduced with permission [197]. Copyright 2021, American Chemical Society. (f) Hierarchical microstructures synergistically fabricated by photolithographic micropatterns combined with nano‐ZnO arrays. Reproduced with permission [202]. Copyright 2023, Wiley‐VCH. (g) Schematic illustration of hydrophobicity and underwater adhesion of mushroom‐headed micropillar arrays. Reproduced with permission [206]. Copyright 2023, American Institute of Physics. (h) Mushroom‐headed micropillar arrays with controlled inclination angles achieved by tuning photolithographic parameters. Reproduced with permission [207]. Copyright 2021, American Chemical Society. (i) Process flow for transfer printing of SWCNT electrodes onto PDMS. Reproduced with permission [222]. Copyright 2019, American Chemical Society.

Energy‐beam‐based techniques, such as laser processing, enable the in‐situ fabrication of materials on flexible polymer substrates through photothermal and photochemical effects, fundamentally circumventing the issue of weak adhesion at heterogeneous interfaces. For instance, the laser‐induced graphene (LIG) process directly ablates a PI film to form patterned conductive porous graphene. By using the PI film as the substrate and controlling the laser parameters, electrodes can be fabricated directly on the PI film, yielding an electrode‐substrate interface with robust bonding, which can be directly employed in sensor manufacturing [184]. Furthermore, the LIG surface contains abundant functional groups and can be combined with other processes, such as plasma treatment and surface chemical modification, to impart surface wettability. Some researchers have also transferred LIG onto uncured PDMS, allowing PDMS infiltration to enhance interfacial bonding between LIG and PDMS. By further adjusting the laser parameters, the LIG/PDMS surface is textured to obtain rough surface microstructures, significantly increasing the WCA [185]. However, PI films generally exhibit limited stretchability, and PDMS has limited strength. Therefore, as shown in Figure 14a, PI powder, PDMS, and amine‐based polyethylenimine ethoxylated (PEIE) are mixed and then subjected to laser ablation. The PI formed porous 3D LIG, while the PDMS surface was converted into semiconducting silicon carbide and SiO_2_. Compared to LIG@PI, the LIG@PI/PDMS/PEIE exhibited enhanced stretchability and adhesion, maintaining a more porous structure. This approach also avoids the poor adhesion issue encountered when transferring LIG@PI onto a PDMS film [18, 19, 20, 21, 22, 23, 24, 25, 26, 27, 28, 29, 30, 31, 32, 33, 34, 35, 36, 37, 38, 39, 40, 41, 42, 43, 44, 45, 46, 47, 48, 49, 50, 51, 52, 53, 54, 55, 56, 57, 58, 59, 60, 61, 62, 63, 64, 65, 66]. Protrusions of LIG may still exist at the edges of laser‐fabricated electrode lines. Therefore, combining the LIG process with stencil masking can produce finer electrodes, as shown in Figure 14b. The s‐LIG possesses a hexagonal lattice and exhibits higher electrical conductivity [187]. Although LIG can be used to directly fabricate sensor electrodes on flexible substrates, its application in electronic circuitry remains relatively limited. To address this challenge, selective copper electrodeposition can be combined with LIG serving as a seed layer to form highly conductive electroplated LIG interconnects. These interconnects are compatible with surface mount device assembly, reflow soldering, and PDMS coating. Moreover, laser rewriting technology allows for the on‐demand repair of damaged circuits [23]. Integration with flash Joule heating technology enables atomic‐level structural reconstruction of LIG, resulting in a more complete hexagonal lattice and rapid repair of LIG. This process enhances crystallinity and electrical conductivity, leading to improved sensing performance in strain sensors [188]. Although laser processing can improve conductivity and interfacial bonding, excessive photothermal damage or edge defects may create local stress concentration and accelerate fatigue failure.

### 6.2 Physical Vapor Deposition

PVD techniques, including magnetron sputtering, IBD, EBD, and evaporation deposition, enable the construction of nanoscale functional interfacial layers on substrates. By regulating the film composition, crystallographic orientation, and stress state, these ultra‐thin layers can serve as hydrophobic barriers, adhesion‐promoting layers, or interfacial bonding layers, while also achieving electrical compatibility. A Zn film deposited on a PDMS surface via direct current magnetron sputtering exhibited thickness‐dependent surface hydrophobicity. As shown in Figure 14c, a Zn layer exhibited a WCA greater than 160° and demonstrated self‐cleaning capability against NaCl particles, sugar particles, sand grains, and chalk dust [189]. By optimizing the magnetron sputtering parameters, indium tin oxide electrodes, zinc aluminum oxide piezoelectric layers, and Cu electrodes are deposited onto PDMS. Owing to the nano‐scale thickness of the sputtered films, the resulting surfaces are smooth with distinct layer boundaries and good interfacial adhesion [190]. By optimizing the dimensions of Cu serpentine electrodes and the thickness of the Ti/PI adhesion layer, maximum adhesion is achieved with appropriate interconnect thickness and electrode dimensions [191]. A Si nanolayer was deposited on poly(ethylene 2, 6‐naphthalate) (PEN) films via ion beam sputtering to prevent polymer damage, followed by surface activation and Fe nanolayer sputtering. Interfacial bonding was achieved under pressure, including Fe–Si silicide formation and Si–O–C/Si–C bonds between Si and PEN [192]. The formation of nanoscale bonding layers improves interfacial load transfer and can reduce delamination‐related resistance drift in thin‐film flexible devices. In summary, the process of converting materials into a vapor phase followed by condensation onto a substrate allows for the uniform and dense deposition of functional films with good adhesion to the substrate. Film thickness and surface roughness can be precisely controlled by adjusting process parameters such as power, pressure, and temperature, thereby improving the adhesion and electrical performance of subsequent flexible mechanical electronic materials. However, vapor‐deposited rigid films may still suffer from cracking under large deformation, so film thickness, residual stress, and adhesion‐layer design must be optimized together.

### 6.3 Plasma and Radiation Surface Activation

Plasma and UVO treatments achieve selective chemical modification and physical cleaning of polymer surfaces through highly active particles, such as free radicals and atomic oxygen. These processes introduce polar groups at the molecular level and eliminate weak boundary layers, thereby synergistically enhancing the adhesion and wettability of functional layers. As shown in Figure 14d, the WCAs of PDMS films are compared under three conditions (untreated, plasma‐treated, and UVO‐treated) [193, 194]. Plasma treatment of PDMS microchannels significantly improves surface hydrophilicity, and subsequent coating with MWCNT dispersion enhances the interfacial adhesion between the two surfaces [195]. Surface activation of the PVDF layer in a flexible device by plasma treatment induces defluorination and introduces radical sites on the PVDF surface. This is followed by a low‐temperature atomic layer deposition process to uniformly deposit ZnO as a piezoresistive layer, where the active radicals facilitate ZnO deposition and ensure uniform thickness [196]. Uniform deposition on activated surfaces helps reduce local electrical nonuniformity and improves repeatable pressure or strain sensing. AgNW films were plasma‐treated with different gases to control dispersion and remove PVP. O_2_ plasma‐treated PET substrates allowed uniform deposition of PVP‐coated AgNWs, followed by N_2_ plasma to decompose PVP into volatile fragments, enhancing conductivity and enabling tight AgNW welding (Figure 14e) [197]. PI substrates were plasma‐treated and functionalized with APTES. Ag^+^ and catalysts were plasma‐activated before deposition onto the APTES‐modified surface, where amine coordination and plasma‐assisted reduction/enhanced electroless deposition synergistically strengthen Ag–PI interfacial adhesion [198]. Using a high‐toughness PI film as the substrate, a water vapor plasma‐assisted bonding method is employed to bond the gold electrodes of a silicon strain gauge with gold leads on the substrate. This method achieves a robust, adhesive‐free interface, enabling highly stable signal output from the bending sensor. This adhesive‐free interface is beneficial for reducing interfacial creep and signal drift because no soft adhesive layer is introduced between the electrode and the substrate [199]. Plasma treatment of a flexible printed circuit substrate, followed by adhesion of anisotropic conductive films, shifts the stress concentration location from the flexible electronic interface to the interior of the conductive film, thereby mitigating interfacial stress concentration during bending [200]. After irradiating both PDMS and PET surfaces with O_2_ plasma, immersing them in an APTES solution not only allows the silane coupling agent to bond with the flexible substrate, but also enables the two different film interfaces to be tightly bonded through covalent interactions [201]. However, it is important to note that the surface modifications induced by plasma and UVO treatments may not be permanent. Immediate subsequent processing is often required to significantly enhance the interfacial adhesion in multi‐layer devices.

### 6.4 Microstructure Fabrication via Physical Masks and Templates

Photolithography and nanoimprint lithography utilize physical masks or templates to replicate micro/nanostructures on flexible surfaces through light‐shielding effects or mechanical contact. By designing customizable morphological molds and subsequently performing demolding, functionalities such as surface hydrophobization or interfacial adhesion can be achieved.

The design of surface structures can change the hydrophobic properties of thin films. For instance, hierarchical microstructures fabricated via photolithography, combined with hydrothermal growth of nano‐ZnO and subsequent deposition of 1H, 1H, 2H, 2H‐Perfluorooctyltriethoxysilane, synergistically enhance surface hydrophobicity, as shown in Figure 14f [202]. Mushroom micropillar arrays were fabricated via stereolithography, mimicking gecko‐inspired structures that enable physical adhesion to various surfaces and allow interfacial bonding through structural design [24, 31, 203, 204, 205]. As shown in Figure 14g, such arrays fabricated from PDMS exhibit dual functionality, including surface hydrophobicity and self‐adhesion. By adjusting the spacing and dimensions of the micropillar arrays, underwater interfacial adsorption becomes achievable [206]. Moreover, by controlling parameters such as backside exposure time during photolithography, elastomer modulus and curing shrinkage ratio, inclined adhesive microstructures can be fabricated, as shown in Figure 14h. These structures leverage combined van der Waals forces, suction effects, and capillary interactions to enhance adhesion strength on both dry and submerged surfaces [207]. Additionally, surface deposition of MWCNT or direct molding of MWCNT/PDMS composites extends their applicability to various flexible electronic devices [208]. A photolithographic process has been employed to fabricate a grid pattern incorporating tentacles and tips. AgNWs are deposited into the grooves of this grid, followed by adhesion transfer of the AgNWs using an elastic polyurethane acrylate polymer. This process yields a self‐attachable, flexible, transparent and conductive electrode. The grid, tentacles and tips enable tight adhesion to various substrates, and the high adhesion facilitates low contact resistance, thereby ensuring good electrical contact with flexible electrodes and mitigating the influence of circuit contact resistance during sensor integration [209].

Beyond photolithography, alternative templating methods also enable surface functionalization. For example, a biomimetic fish‐scale structure on SR was fabricated via a mesh‐assisted templating method, combined with spray‐coated GO/CNTs conductive films. The resulting strain sensor exhibited a superhydrophobic surface with a WCA greater than 150° and complete water droplet roll‐off at an 8° tilt [210]. In another approach, chemical etching of an Al template generates hierarchical surface microstructures, which are then replicated via templating into PDMS for microfluidic devices. Under optimal etching conditions, the structured surface achieved a WCA of 154° and a roll‐off angle below 5° [211]. Template‐assisted microstructures are effective for combining self‐cleaning and adhesion regulation, but fabrication precision, demolding damage, and wear resistance remain important limitations for large‐area devices.

### 6.5 Printing and Coating

Printing technology is a common, low‐cost, customizable, and potentially mass‐producible process that transfers liquid or solid materials onto a substrate surface. Common methods include 3D printing, inkjet printing, screen printing, roll‐to‐roll (R2R) printing, water transfer printing, and pad printing [212, 213, 214, 215, 216]. Coating technology involves applying liquid materials to a substrate surface to form a protective or functional layer, improving the surface properties of flexible electronic devices [217, 218]. However, adjusting the viscosity of the ink and the properties of the material plays a decisive role in the surface functionality and interfacial adhesion of flexible devices. Printing and coating processes offer an efficient and controllable manufacturing method for improving surface properties and interfacial adhesion.

CB‐PDMS/Ecoflex is used as an interconnection material, which is selectively deposited via coating onto the interconnect region between the PDMS/AgNW sensitive layer and the screen‐printed Ag electrodes. The dual elastomers facilitate curing of the Ag paste and enhance interfacial adhesion. Furthermore, large‐area screen printing technology is employed to enable high‐throughput fabrication of interconnections for arrayed Ag electrodes [219]. Screen printing is further utilized in the fabrication of multi‐layer bending sensors, where highly conductive Ag paste arrays are printed onto a graphene sensing layer. The interfacial effects amplify the sharp increase in resistance caused by changes in electron pathways during bending, thereby enhancing sensing performance [107]. Dip‐coating and screen‐printing techniques have also been employed to fabricate MXene‐based paper sensors, where conductive networks and printed electrodes can be integrated onto breathable paper substrates, providing a scalable and low‐cost route for flexible wearable electronics [220]. Ag interdigitated electrodes were deposited via inkjet printing on KOH‐treated PI films. Stepwise curing of the printed electrodes induces Ag^+^ reduction, enabling in situ growth of Ag particles on the PI substrate and resulting in strong interfacial adhesion [221]. As shown in Figure 14i, a porous hydrophilic hydrogel PAAm is designed, and SWCNT ink is subsequently deposited onto its surface by inkjet printing. Due to the surface‐only deposition of hydrophobic conductive materials on the PAAm, they are readily transferable onto elastomeric substrates. To achieve more robust adhesion of the SWCNT electrodes to the elastic substrate, a PDMS prepolymer solution is applied for transfer printing, followed by curing [222]. PDMS microstructured films are treated with piranha solution to temporarily render the surface hydrophilic, followed by spin‐coating of a PVA layer to maintain long‐term hydrophilicity. A water‐based conductive screen‐printing ink is then prepared, and pad printing is employed to deposit functional materials onto the hydrophilized PDMS microstructures, enabling rapid fabrication of microstructured sensing layers for pressure sensors [216]. Triply periodic minimal surface topologies are designed, and piezoresistive structures for TPU sensors are fabricated layer‐by‐layer using 3D printing. Graphene ink is subsequently dip‐coated onto the structure, and the interconnected topology is demonstrated to provide additional percolation pathways, enhancing the electrical conductivity of the devices [223]. A MWCNT/acetone suspension is sprayed onto polycarbonate (PC) surfaces using R2R spray coating, followed by hot pressing to embed the MWCNTs into the PC film. The hot‐pressing temperature is adjusted close to the glass transition temperature of PC, which enhances the interfacial adhesion between MWCNTs and PC without significantly affecting the surface smoothness or mechanical properties of the PC [224]. Damage‐free dry transfer printing of ultrathin films with on‐demand interfacial adhesion has been summarized as an important strategy for heterogeneous integration in flexible and curved electronics. By regulating adhesion at the donor/film, film/stamp, and film/receiver interfaces, transfer‐induced cracking, delamination, and in‐plane film damage can be reduced during pickup and release processes [225]. The sensing layer was deposited onto a spin‐coated water‐soluble PVA layer on PET films and subsequently transferred, together with the PVA support, onto target substrates such as leaves or textiles. The water‐soluble PVA layer can achieve strong adhesion of the sensor to the curved substrate by relying on the water‐assisted uniform stress distribution and van der Waals force enhancement [226].

In summary, printing and coating techniques are efficiently employed to impart waterproofing, antifouling, and anticorrosion functionalities to mechanically coupled flexible electronics. These methods are further utilized to tune surface wettability, wear resistance, and electrical conductivity. Moreover, the thickness and architecture of functional layers are precisely controlled, thereby regulating the electrical and mechanical properties of the devices and ensuring their stability under extreme conditions. However, printing and coating performance strongly depends on ink rheology, solvent evaporation, curing conditions, and substrate wettability, which may affect coating uniformity, adhesion, and long‐term output reproducibility.

## 7 Multiscale Architectonics in Surface and Interfacial Engineering

Appropriate material selection establishes the foundational functionality, mechanical performance, and stretchability of flexible electronic devices. Building on this foundation, chemical and physical strategies have been widely employed to tailor surface and interfacial properties, yet single approaches remain limited in simultaneously enhancing multi‐layer adhesion, surface functionality, and device reliability. To address these challenges, current research increasingly focuses on multiscale interfacial architectonics, integrating complementary chemical, physical, and structural strategies to create adaptive, strain‐dissipating interfacial layers that synergistically combine mechanical durability with electrical continuity.

### 7.1 Bioinspired Surface Structures and Mechanical Interlocking

Inspired by nature, the design and fabrication of surface microstructures are developed to achieve multiple stable surface functionalizations, solving issues such as surface self‐cleaning, self‐adhesion, and anti‐friction in flexible devices. The design of interfacial microstructures enables the fabrication of mechanical interlocking structures, which have emerged as a robust physical bonding mechanism that transcends intermolecular forces, providing extremely high interface toughness.

**FIGURE 15:**
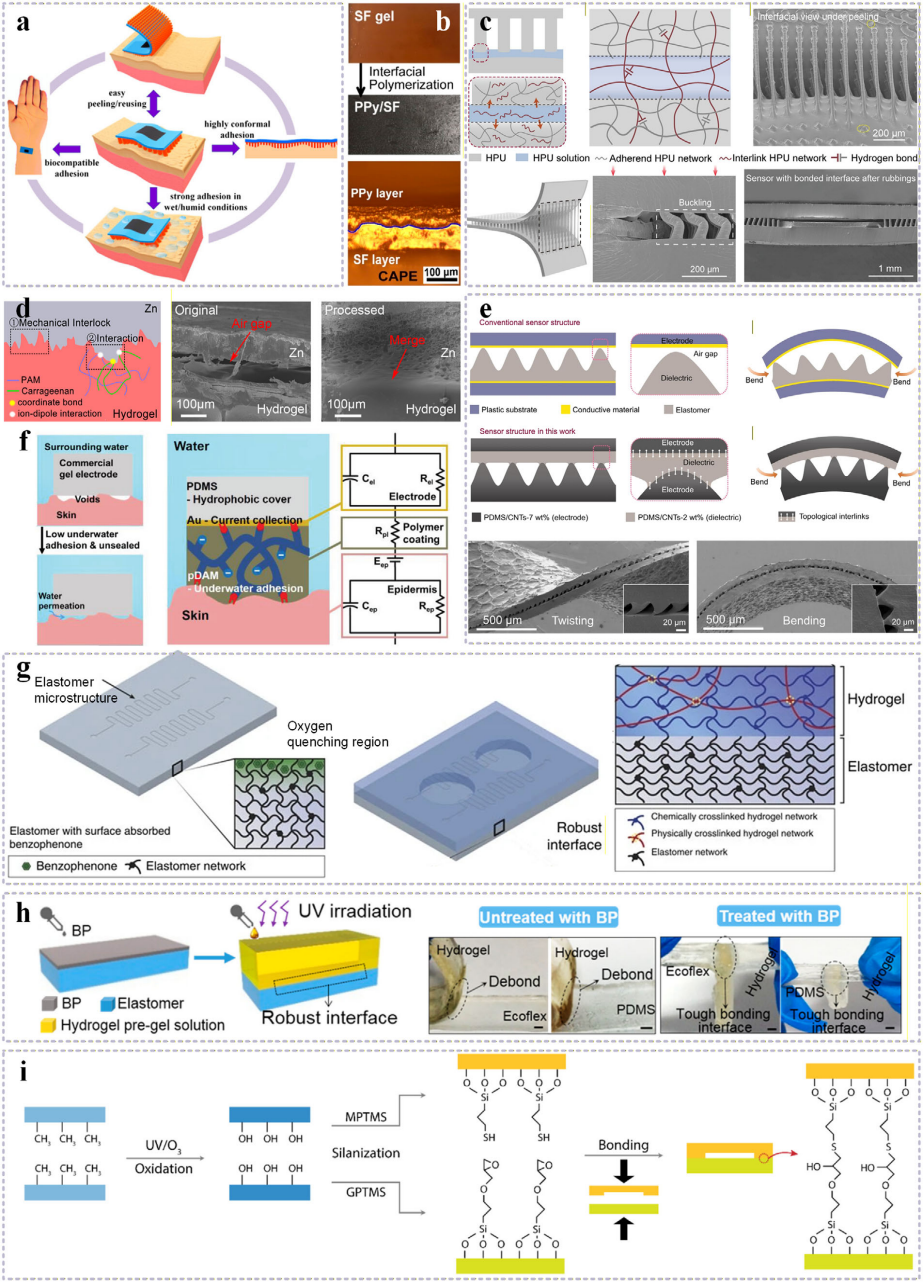
Bioinspired surface structures and mechanical interlocking: (a) Reversible adhesion achieved by SF combined with microstructures. Reproduced under the terms of the Creative Commons Attribution 4.0 International License [226]. Copyright 2020, American Chemical Society. (b) Interfacial mechanical interlocking between SF and PPy. Reproduced with permission [109]. Copyright 2020, American Chemical Society. (c) Design of HPU interface micropillar structure. Reproduced with permission [26]. Copyright 2025, Elsevier Ltd. (d) Mechanical interlocking structure at hydrogel and Zn electrode interfaces. Reproduced with permission [235]. Copyright 2024, American Chemical Society. (e) Mechanical interlocking interface in PDMS‐CNTs multi‐layer capacitive sensor. Reproduced under the terms of the Creative Commons Attribution 4.0 International License [236]. Copyright 2022, Springer Nature. Interfacial adhesive layers: (f) pDAM serving as a waterproof adhesive layer for Au/PDMS to enable underwater adhesion. Reproduced with permission [239]. Copyright 2024, Wiley‐VCH. (g) Mechanism and process of BP‐mediated elastomer bonding. Reproduced under the terms of the Creative Commons Attribution 4.0 International License [95]. Copyright 2016, Springer Nature. (h) Process and adhesion results for BP‐mediated bonding of hydrogels and PDMS. Reproduced with permission [241]. Copyright 2018, American Chemical Society. (i) MPTMS and GPTMS modification of PDMS surface to achieve interfacial bonding. Reproduced with permission [244]. Copyright 2021, American Chemical Society.

Bioinspired surface structures enable diverse surface functionalities for flexible electronic devices. Microstructured PDMS films with micropillar arrays are fabricated using template methods, followed by O_2_ plasma treatment and spin‐coating of silk fibroin (SF) solution to form an adhesive layer. This bioinspired adhesive, combined with the micropillar structures, has the ability to repeatedly adhere to the skin surface, as shown in Figure 15a [227]. A microporous‐crack strain sensor inspired by scorpion sensory structures is composed of a composite material of superhydrophobic TPU, CNTs, and graphene. The Archimedean spiral cracks on the surface, combined with the superhydrophobic properties of TPU, achieve surface superhydrophobicity and signal stability [8]. A shark skin‐inspired LM/PDMS structure is fabricated by DIW, followed by spray coating with an antistatic spray containing PDMS, bisphenol A, (3‐Aminopropyl)triethoxysilane (APTMS), and SiO_2_. This design can achieve surface drag reduction and antifouling properties [128]. Surface structural design also improves conformal contact with target objects such as human skin, thereby reducing MAs. For example, a conformal and adhesive polymer electrode (CAPE) is developed by interfacial polymerization of PPy with SF gel, forming an interlocking interface as shown in Figure 15b. This design increases interfacial contact area and frictional forces, effectively enhancing interfacial adhesion while ensuring high mechanical matching between SF and skin [109].

Interface mechanical interlocking structure design has become increasingly prevalent. Whether it is achieved through microstructure physical manufacturing or by combining material deposition processes and chemical reactions to achieve interface mechanical interlocking, it greatly improves the interfacial toughness and device stability of multi‐layer mechanically coupled flexible electronics. As shown in Figure 15c, high‐branching PU (HPU) is used as the base material, and its strong molecular structure is utilized to design a micropillar structure to connect the device interface. By controlling the diameter and density of the micropillars, the tensile strength and ductility of the interface are improved, and structural defect propagation is avoided, and the toughness, elastic deformation behavior, and strain energy storage and release are enhanced, allowing it to withstand large deformations [26]. MXene and porous LIG are combined to achieve mechanical interlocking inside the LIG electrode, resulting in high adhesion strength. The fabricated TENG can be used for stable energy harvesting and finger bending detection without distortion [228]. After O_2_ plasma treatment of the PET surface to maintain its roughness, chemically plated copper electrodes can form a mechanical interlocking structure with the flexible substrate interface, greatly improving the interfacial adhesion strength [229]. Hydrogels infiltrate into a porous TPU fiber network to form a mechanical interlocking structure, ensuring stable adhesion between the hydrogel and TPU. A gold nanofilm is then thermally evaporated onto the TPU fiber, resulting in a strain sensor exhibiting excellent stability and high adhesion strength [230]. Polyester hotmelt adhesive film (PES HMA) is infiltrated into the pores of a porous flexible PI substrate using a R2R process. After thermal bonding, it forms a mechanical interlocking structure, significantly improving the interfacial adhesion between rigid components on PI and the flexible TPU substrate [231]. A double‐layered cross‐shaped 3D microelectrode structure is designed, where mechanical interlocking is achieved through mesh‐like microchannels. This design avoids mechanical mismatch between the layered electrode and functional material layer, improving the stability of a flexible piezoelectric bending sensor [232].

The design of the mechanical interlocking structure not only improves interfacial adhesion but also helps address the overall mechanical matching challenges of the device. A bioinspired full‐interface engineering strategy, inspired by plant root architectures, designs varied root structures and dimensions to optimize interfacial mechanical interlocking, achieving efficient interface transition and mitigating modulus mismatch between rigid and flexible contact regions [233]. The bottom edges of cylindrical arrays in conductive microstructured films are wetted with highly adhesive polymers and assembled face‐to‐face to realize mechanical interlocking. This configuration enables solderless, high‐strength interconnections in multi‐layer stretchable electronics while preventing stress concentration associated with rigid solder joints [234]. Electrochemical treatment is further employed to construct dense interlocking structures between hydrogels and Zn electrodes as shown in Figure 15d, significantly enhancing interfacial adhesion. Controlling the local modulus of the hydrogel also achieves strain isolation, protecting the electrodes from high strain conditions [235].

While planar interfaces can be stabilized via chemical modifications, mechanically interlocked structures are required to achieve high interfacial toughness when microstructured electrodes, dielectric layers, or piezoresistive layers are integrated with planar surfaces in flexible sensors. For instance, by using multi‐layer quasi‐homogeneous materials, PDMS/CNT composites were tuned to serve simultaneously as electrodes and dielectric layers. The layers were swollen in an organic solvent/PDMS prepolymer system, laminated, and then pressed and cured. As shown in Figure 15e, this forms a new PDMS network, establishing interfacial topological connections. The resulting mechanically interlocked pressure sensors endure up to 10^5^ friction cycles [236]. Moreover, such topological contact reduces interfacial friction and energy dissipation, enabling ultrafast sensor response [237]. Overall, bioinspired structures and mechanical interlocking improve reliability by increasing contact area, distributing stress, and suppressing interfacial displacement. However, their practical implementation is often constrained by fabrication complexity, surface wear, contamination sensitivity, and batch‐to‐batch structural variability.

### 7.2 Interfacial Adhesive Layers

A functionalized adhesive layer is designed to directly connect the functional layer and substrate, serving not only to enhance adhesion but also to provide multifunctionality and structural integration, making it a key element for multi‐layer device integration and interfacial performance. For example, a hydrophobic eutectogel (HEG) is designed as an interfacial adhesive layer to achieve simultaneously surface and interfacial adhesion underwater and in air. Multi‐functional properties, including mechanical robustness, ionic conductivity, and high adhesiveness, are realized through the UV photopolymerization of different acrylates, a photoinitiator, and a hydrophobic composite. Due to multiple non‐covalent interactions, the adhesion strength of HEG facilitates conformal contact with various substrates, enabling the application of sensors in subaquatic scenarios [25]. Furthermore, a solvent‐free poly(ionic liquid) elastomer (PILE) is designed as an adhesion layer. The highly hydrophobic acrylate ILs have strong electrostatic interactions, which can achieve high surface and interfacial adhesion underwater [238]. An iontronic polymer can enable Au/PDMS sensors to adhere tightly to human skin underwater. As shown in Figure 15f, polymer p(DMA‐*co*‐AA‐*co*‐MEA) (pDAM), copolymerized from dopamine methacrylamide (DMA), acrylic acid, and methoxyethyl acrylate (MEA), was drop‐cast onto Au/PDMS surfaces to form a waterproof layer, with dopamine providing strong interfacial adhesion [239].

A dual mechanism involving radical or cation generation by photoinitiators, combined with their permeation into polymer networks, enables adhesion between different elastomer interfaces. Benzophenone (BP), as a photoinitiator, is dissolved in ethanol or acetone and diffuses to various elastomer surfaces under oxygen‐free conditions. After UV irradiation, BP generates methyl radicals on the elastomer surface, which can rapidly graft various polymers. Benzopinacol is the product of interfacial adhesion, enabling interfacial adhesion of various elastomers and hydrogels [95, 240, 241, 242]. As shown in Figure 15g, BP has successfully penetrated into the elastomer network, effectively bonding PDMS, PU, latex, VHB, Ecoflex, and various hydrogel interfaces [95]. As shown in Figure 15h, BP‐mediated interfacial modification enables effective bonding between PDMS and PAAm‐SA hydrogels. Acting as an initiator, BP promotes hydrogel polymerization at reactive sites on the elastomer surface, and the resulting structure is employed as a TENG electrode for human motion monitoring [241]. PEGDA is inkjet‐printed onto the PDMS surface with an array microstructure, and BP is added followed by UV irradiation. Through the combined action of the inkjet printing and BP, PEGDA is covalently bonded into the PDMS elastomer network [240]. In addition to BP, poly{2‐ [2‐(methacryloyloxy) ethyldimethylanmmonium] ethyl benzophenoxy phosphate} (PMBPP) can also serve as a photoinitiator to impart long‐lasting hydrophilicity to PDMS surfaces [243].

Designing adhesive coupling agents on the surface of a flexible substrate can achieve effective adhesion between the substrate and the surface functional layer. After UVO treatment of PDMS, two PDMS films are respectively modified with MPTMS and (3‐glycidyloxypropyl)trimethoxysilane (GPTMS). The two surface‐modified PDMS films are then bonded under 40 kPa pressure. As shown in Figure 15i, the thiol‐epoxy click reaction achieves covalent bonding between the PDMS films [244]. Au surfaces treated with MPTMS can be bonded with partially cured PDMS, followed by complete curing to achieve stronger interfacial adhesion [245].

The design of the interfacial adhesive layer improves interfacial toughness and reduces interfacial delamination. For example, a poly(2‐ethylhexyl acrylate) interfacial adhesive layer is introduced to chemically bond a humidity‐responsive layer (PAM hydrogel) with an inert PET film. The resulting humidity‐driven multi‐layer flexible actuator maintains stable interfacial integrity under humidity variations, and differential swelling does not induce delamination [246]. A TPU interfacial adhesive layer is introduced between a flexible conductive fabric and a porous thermosetting PU‐based ionogel layer, forming a stable interfacial bond. This configuration eliminates the need for encapsulation, improves electromechanical coupling, and reduces delamination between layers with different moduli [30]. Adhesive interlayers are therefore effective stress‐transfer bridges between mechanically mismatched layers, but their modulus and thickness must be optimized to avoid reduced sensitivity, slower response, or additional interfacial failure.

### 7.3 Interfacial Mechanical Buffer Layers and Gradient Transition Architectures

**FIGURE 16:**
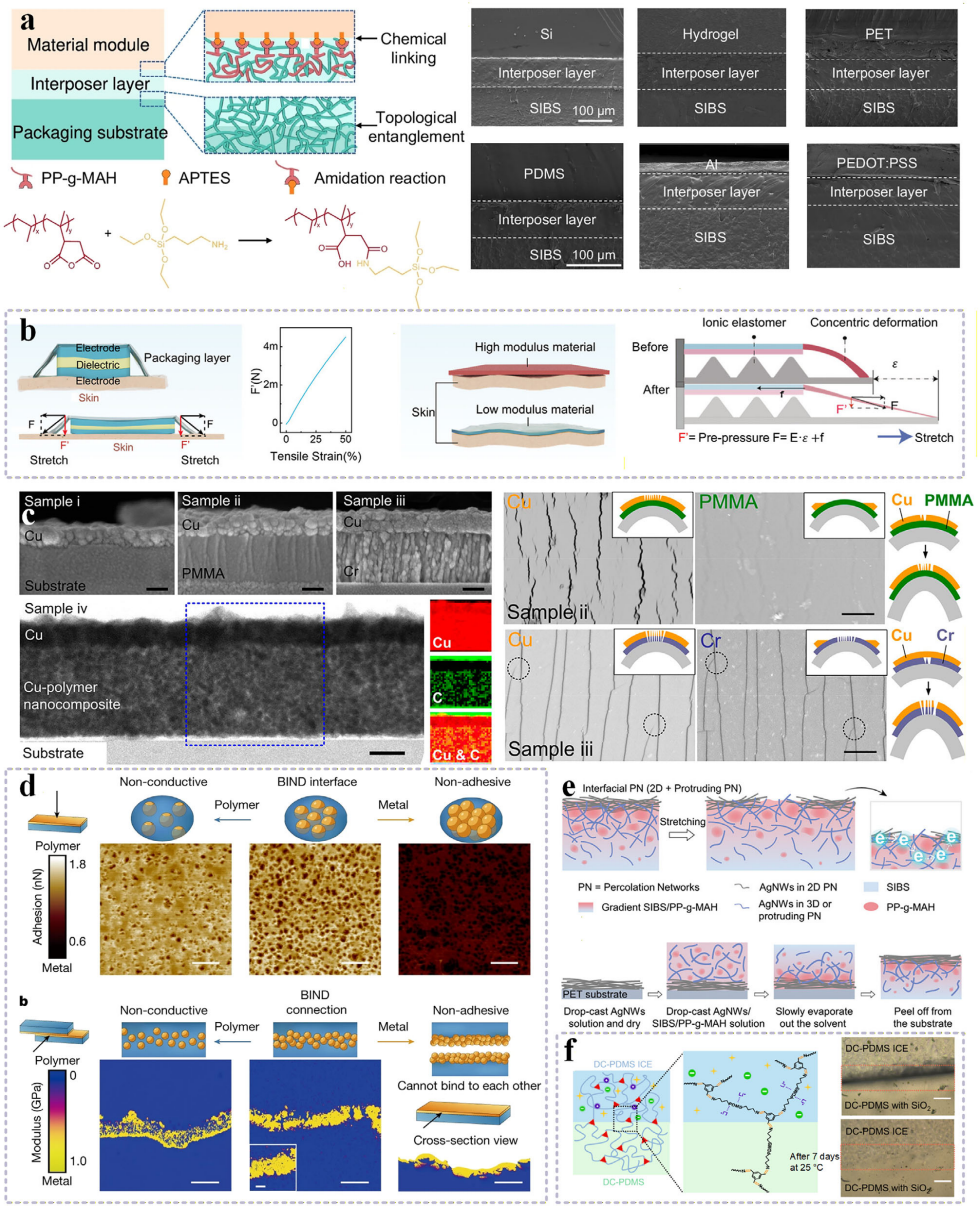
Interfacial mechanical buffer layers and gradient transition architectures: (a) The interposer layer and the SEM cross‐section of its adhesion between SIBS and other flexible substrates. Reproduced under the terms of the Creative Commons Attribution 4.0 International License [101]. Copyright 2024, Springer Nature. (b) Structural design for reducing MAs. Reproduced under the terms of the Creative Commons Attribution 4.0 International License [16]. Copyright 2025, Wiley‐VCH. (c) Mechanism of elasto‐plastic interlayers for alleviating mechanical mismatch. Reproduced with permission [100]. Copyright 2023, American Chemical Society. Interfacial percolation and interpenetrating networks designs: (d) Design of the bind interface. Reproduced with permission [247]. Copyright 2023, Springer Nature. (e) Interfacial percolation network strategy. Reproduced with permission [251]. Copyright 2024, Wiley‐VCH. Interfacial homogeneity strategies: (f) Homogeneous interfacial bonding between the DC PDMS‐ICE electrode and the DC PDMS/SiO_2_ dielectric layer. Reproduced with permission [253]. Copyright 2023, Tsinghua University Press.

Introducing a mechanical buffer layer can actively absorb and dissipate strain energy during device operation, while its own deformation alleviates interfacial stress concentration caused by modulus mismatch between soft and rigid layers. A novel universal interfacial interposer layer based on poly(styrene‐isobutylene‐styrene) (SIBS) and maleic anhydride‐grafted polypropylene (PP‐*g*‐MAH) elastomer is designed, simultaneously achieving topological entanglement with the SIBS substrate and chemical bonding with other surfaces. As shown in Figure 16a, this interposer layer, after APTES surface treatment, can also chemically bond SIBS with Si, PDMS, hydrogels, Al, PET, and PEDOT [101]. To suppress MAs during device operation, a strain‐insensitive sensor with multi‐modulus composition is designed, as shown in Figure 16b. A composite of poly(vinylidene fluoride‐co‐hexafluoropropylene) (P(VDF‐TrFE)) and the ILs (1‐ethyl‐3‐methylimidazolium bis(trifluoromethylsulfonyl)imide) is employed as a microstructured iontronic layer. Low‐modulus PDMS, primarily subjected to tensile deformation, is gel‐bonded to PU to form a soft/hard encapsulation layer, reducing MAs caused by normal stress. In addition, a conductive lubricant reduced the friction between the ionization layer and the rigid electrodes [16]. In multi‐layer devices, an elasto‐plastic interlayer material composed of PMMA and Cr is designed. Despite being less than 100 nm in thickness, this composite exhibited high Young's modulus and high yield strain. Under mechanical loading, cracks preferentially initiate within the Cr layer rather than the Cu electrode layer, as shown in Figure 16c. Furthermore, this method is applicable to other multi‐layer interface devices with elasto‐plastic characteristics [100]. An island‐bridge architecture was introduced into the iontronic sensor, with non‐iontronic regions embedded in PDMS as isolation chambers for the ion gel, thereby elongating the interpore walls to dissipate energy and enhance interfacial toughness. In addition, mechanical interlocking between Au electrodes and PDMS/CNT formed a gradient stress structure, avoiding stress concentration and structural stiffening during device loading and providing enhanced stretchability [15]. Gradient and buffer architectures improve long‐term reliability by redistributing strain away from brittle or conductive regions, but they may introduce trade‐offs among thickness, sensitivity, response speed, and fabrication complexity.

### 7.4 Interfacial Percolation and Interpenetrating Networks Designs

Unlike structural design or added interfacial layers, interpenetrating networks arise from mutual infiltration and entanglement of two phases into an integrated interface. Their nano/micro scale interpenetration greatly increases contact area and debonding energy, enabling robust electromechanical coupling. A universal bind interface, composed of interpenetrating polymers and metallic nanomaterials, forms a strongly adhesive, versatile soft electronic interface through simple pressure application without the need for pastes. As shown in Figure 16d, this biphasic network growth model relies on the thermal evaporation of Au or Ag nanoparticles to form an interpenetrating network with self‐adhesive styrene–ethylene–butylene–styrene (SEBS). By regulating the component ratio and degree of penetration, a conductive layer is maintained on the surface while partially conductive particles become fully infiltrated within the SEBS, forming a biphasic nanolayer that improves signal fidelity by maintaining conductive continuity even when the device is stretched, compressed, or rubbed [247]. A diffusion‐induced cohesion strategy is developed without chemical modification, where interfacial bonding between metals and elastomers is enhanced through molecular diffusion, a hydrogen bond network, and cohesive effects. For instance, the hydrophilicity of waterborne PU (WPU) is utilized to facilitate the formation of a stable hydrogen bond network between deposited Au particles and WPU during the deposition process via the diffusion of water molecules and oxygen‐containing groups. Concurrently, the rough microporous structure on the WPU increases the contact area with Au, further enhancing the interfacial cohesion and significantly improving the friction resistance and electromechanical stability of Au‐WPU devices [248]. Flexible elastomer surfaces are treated with N, N‐Dimethylformamide to expose–NH and–OH groups. Subsequent inkjet deposition of LM allows its gallium oxide shell to form hydrogen bonds, with Ga, In, C, and O elements from the LM penetrating into the soft substrate layer. The boundary between the bilayer films exhibits a diffusion trend, thereby forming a robust interface [249]. The surface of parylene was treated with water‐vapor plasma to render it hydrophilic, followed by droplet injection for plasticization. Atomic‐level bonding between the Au electrode and the parylene substrate is achieved directly at low temperature, where metal atom diffusion and molecular chain entanglement contribute to high‐strength interfacial connections [250]. Introducing an ionic monomer solution onto the surface of silicone‐modified PU (SiPU)/CNT composites, followed by UV irradiation, forms an elastomer. The polymer chains interweave and interpenetrate with the SiPU molecular chains, thereby enhancing interfacial bonding strength [29]. By regulating the deposition and distribution of AgNWs and incorporating PP‐*g*‐MAH, 3D protrusions are formed on the AgNW surface. As shown in Figure 16e, an interfacial percolation network (PN) design integrates a 2D AgNWs network with protruding 3D AgNWs structures, resulting in highly conductive stretchable electrodes [251]. Dual‐network stretchable adhesives combine a soft siloxane network and a rigid polycyanoacrylate network to form molecular‐scale hooking‐interlocking structures at heterogeneous soft material interfaces. This interpenetrating architecture enables efficient, slip‐free strain transfer for flexible sensors [252]. Interpenetrating networks are powerful for suppressing interface slipping and resistance drift, but excessive network density may increase stiffness and reduce mechanical compliance.

### 7.5 Interfacial Homogeneity Strategies

Device interfaces are often composed of heterogeneous materials, leading to interfacial issues due to inherent mechanical mismatch. Homogeneity strategies, by closely matching the moduli of interfacial materials, enhance the robustness and fatigue resistance of sensors under repeated mechanical stress. By tuning the ratio of PDMS to CNTs, a composite material can be fabricated to simultaneously serve as both the electrode and the sensing layer of a capacitive pressure sensor. The use of homogeneous materials and their closely matched mechanical moduli effectively avoids interfacial mechanical mismatch [235]. By modulating the uniformity of CNTs within TPU, CNT‐TPU composites (CTCs) with different levels of fineness are fabricated. Although these elastomers exhibit different electrical and mechanical properties due to variations in dispersion and mass fraction, excellent interfacial bonding is achieved between the sensing and interconnecting components of the strain sensor owing to material homogeneity. Design of non‐uniform CTCs also exhibits a better signal‐to‐noise ratio [106]. Self‐healing dynamically cross‐linked PDMS (DC‐PDMS) is designed and applied to both the ionic conductive electrode layer (DC PDMS‐ICE) and the dielectric layer (DC PDMS/SiO_2_). Owing to the self‐healing properties and material homogeneity of the matrix, dynamic imine bonds form spontaneously at the interface without any external stimulus, as shown in Figure 16f. This results in strong interfacial bonding and effectively prevents interfacial mechanical mismatch [253]. Similarly, self‐healing PDMS is designed as the substrate material for all components of a flexible pressure sensor, including the substrate, the AgNWs/self‐healing PDMS electrode, and the MWCNT/self‐healing PDMS pressure‐sensing layer. The self‐healing property, dynamic interfacial crosslinking capability, and material homogeneity collectively reduce interfacial mismatch and adhesion challenges in multi‐layer devices [254]. Overall, homogeneity strategies reduce failure from mechanical mismatch and improve fatigue resistance, but they may limit material selection and multifunctional integration when different layers require distinct mechanical, electrical, or environmental properties.

In summary, surface and interfacial adhesion can be effectively reinforced through chemical modification, optimization of physical processing parameters, and advanced architectonics design. These strategies improve conformal coupling with target objects and strengthen the interfacial adhesion of multi‐layered devices, thereby promoting efficient stress transfer, suppressing interfacial delamination, delaying crack propagation, and maintaining electrical transport stability under complex operating conditions. As shown in Table 3, representative surface and interfacial engineering strategies are compared in terms of target failure modes, testing methods, specimen geometries, reported adhesion‐related values, normalized values when available, and device‐level reliability metrics. The listed parameters include adhesion force, peel strength, interfacial toughness, shear stress, cycling durability, performance retention, and stability under wet, sweat, underwater, thermal, or mechanically dynamic environments. It should be noted that values obtained from different testing configurations, such as T‐peel, 90° peel, 180° peel, lap‐shear, tensile detachment, cyclic bending, and cyclic stretching tests, reflect different stress states and energy‐dissipation processes. Therefore, these data should be interpreted together with the corresponding specimen geometry, loading mode, contact area, normalization method, and environmental conditions, rather than being treated as directly equivalent quantities. Overall, Table 3 is intended not only to summarize adhesion‐related performance, but also to establish a clearer connection among engineering strategies, quantitative evaluation, and application‐oriented reliability. By presenting the reported quantitative metrics together with testing methods and specimen configurations, this comparison provides a more transparent basis for interpreting interfacial performance across different studies. It also highlights the importance of evaluating adhesion‐related metrics within their specific experimental contexts, particularly when different loading modes, interface structures, and device configurations are involved.

**TABLE 3:** Interfacial adhesion of mechanically coupled flexible electronics and adhesion to other substrates under different strategies.

Strategy	Bonded materials	Interfacial performance	Test method	Specimen configuration	Device‐level reliability outcome	Reference
Interfacial micropillar arrays	Between HPU layers	Interfacial toughness: 5095 J m^−2^	180° peel test, 50 mm min^−1^	Rectangular samples, 10 mm × 20 mm	Not reported	[26]
Subsurface‐initiated atom transfer radical polymerization with interfacial interlocking and polymer bridging	Cu and PDMS	Adhesion strength: 2.52 MPa	90° peel test	Metal film/PDMS bilayer	98.9% sensitivity retention after 3000 loading/unloading cycles	[183]
Magnetron sputtering with optimized electrode dimensions and adhesion layer thickness	Cu, Ti/PI adhesive and PDMS	Interfacial toughness: 10.09 J m^−2^	In situ uniaxial tensile test with SEM observation	Rectangular composite‐film sample, 20 mm × 10 mm	Suppressed interfacial delamination, optimum adhesion obtained at 75 nm Cu thickness	[191]
O_2_ plasma treatment	PDMS and PET	Interfacial stress: 0.15 MPa	Tensile lap‐shear test, 5 mm min^−1^	Overlap‐bonded specimens, 20 mm × 13 mm bonded area	Survived >5000 strain cycles without delamination	[201]
Physical adsorption via photolithography‐patterned mushroom‐headed microstructures	Between PDMS layers	Peel strength: 0.1 N cm^−1^	Lateral friction (adhesion) test	1 cm^2^ dry‐adhesive sample	Not reported	[31]
Interfacial mechanical interlocking via rough surface deposition coating	Cu and PET	Peel strength: 1300 N cm^−1^	90° T‐peel test, 4 mm min^−1^	PET film: 100 mm × 20 mm × 100 µm Cu layer: ∼100 µm after electroplating	Virtually no increase in electrical resistivity after 300000 bending cycles	[228]
Interfacial mechanical interlocking via hydrogel locking within porous elastomer substrate	Hydrogel and TPU	Interfacial toughness: 50.9 J m^−2^	90° peeling test	Hydrogel thickness: 2 mm; width: 10 mm TPU thickness ≈ 83–85 µm PI thickness: 90 µm	SNR maintained (∼43 dB) up to 150 cycles	[229]
Interfacial mechanical interlocking via PES HMA layer	PI, PES, and TPU	Adhesion force: 8.34 N cm^−1^	180° peel test	10 mm × 10 mm × 25–225 µm	Soft–rigid connection survives ∼700% strain, stable over 2000 cycles at 100–200% strain	[230]
Interfacial mechanical interlocking via mesh‐like microchannels	AgNWs and P(VDF‐TrFE)	Interfacial toughness: 300 J m^−2^	180° peel test	Not reported	Stable piezoelectric output after 1.6 × 10^8^ bending cycles, output attenuation < 7.5% after 50000 cycles	[231]
Interfacial mechanical interlocking via microstructure arrays combined with chemical adhesive	Au and PDMS	Shear adhesion strength: 360 kPa	Shear adhesion test	Not reported	R/R_0_ ≤ 5 at ≈35% strain, electrically stable for >500 cycles at 20% strain	[233]
Interfacial mechanical interlocking via electrochemically fabricated in‐situ dense structure	Zn and hydrogel	Interfacial toughness: 87 J m^−2^	180° peel test	Not reported	8.7× increase in adhesion energy, <10% resistance variation at 270% strain, stable after 3000 cycles at 20% strain	[234]
TPU interfacial adhesion layer	Conductive fabric and porous TPU‐based ionogel	Peel strength: 144.2 N m^−1^; Interfacial toughness: 243 J m^−2^	180° peel test, 50 mm min^−1^	10 mm × 50 mm	Stable over 150000 compression cycles at 100 kPa, signal fluctuation within 7.1%, stable over 10000 compression–shear cycles	[30]
Interpenetrating polymer and metal nanostructures at interface	Au and SEBS	Interfacial toughness: 0.24 N mm^−1^	180° peel test	Not reported	>600% mechanical stretchability and >180% electrical stretchability, stable over 600 tensile, bending, and twisting cycles	[247]
Diffusion‐induced cohesion	Au and WPU	Peel strength: 1243.4 N m^−1^	T‐peel test, 5 mm min^−1^	15 mm × 10 mm	Maintained conductivity after 1022 ± 76 frictions at 130 kPa, stable over 1000 tensile cycles at 100% strain	[248]
Interpenetrating architecture	Siloxane and polycyanoacrylate	Peel strengths 1000 N m^−1^	90° peel test	10 mm × 2 mm × 100 mm	±2% signal variation after 10000 cycles at 50% strain	[252]
Interpenetrating interface between conductive material and elastomer via in‐situ polymerization	SiPU/CNT and ionic elastomer	Interfacial toughness: 5000 J m^−2^	T‐peel test, 100 mm min^−1^	Not reported	Stable reversible adhesion over 60 cycles	[29]
Click chemistry and covalent grafting strategy	Polymers (GelMA, PEGDA) and substrates (latex, PDMS, Ecoflex, TPU)	Shear strength: >130 kPa	Lap‐shear test, 50 mm min^−1^	Two rectangular substrates crossed with 2 cm × 2 cm overlapping area	Stable output for 20000 cycles at 5% strain, 5000 cycles at 10% strain, and 1000 cycles at 50% strain	[60]
C‐CNTs/PA in PAM network	MCTP and wood	Adhesion strength: 18.6 kPa	Lap‐shear test, 5 mm min^−1^	20 mm × 15 mm × 2 mm	Adhesion retained after 10 repeated cycles, >70% adhesion retained after 30 days, stable strain sensing over 1000 cycles at 200% strain	[144]
BIMS via covalent bonds and multiple non‐covalent interactions	PVA/PAA‐NHS ester and marine organism epidermis	Interfacial toughness: 160 J m^−2^	90° peel test, 50 mm min^−1^	15 mm wide sample	Rapid attachment within 22 s, stable adhesion during 40 simulated respiration cycles	[169]
PEIE regulates composite viscosity and crosslink density	PI/PDMS/PEIE and glass slide	Adhesion force: 63.377 N m^−1^	Peel‐off adhesion test, 200 mm min^−1^	Not reported		[186]

## 8 Applications Scenarios Enabled by Surface and Interface Engineering

Although the preceding sections have summarized surface and interfacial engineering strategies from the perspectives of materials design, surface chemistry, interfacial bonding, physical processing, and multiscale architectures, their practical significance is ultimately reflected in application‐specific reliability. Different service environments impose distinct dominant failure modes, such as unstable surface contact in wearable biosensing, interfacial delamination and stress concentration in motion or industrial monitoring, and electrical‐pathway fluctuation under acoustic or vibration stimuli. Therefore, before discussing representative applications, it is necessary to further connect each failure mode with its corresponding physical mechanism, engineering strategy, reliability metric, and application scenario. As summarized in Table 4, surface contact regulation, interfacial adhesion enhancement, stress redistribution, crack suppression, environmental protection, and electrical‐pathway stabilization jointly address the compatibility challenge between mechanical robustness and electrical functionality. These strategies remove key barriers to the practical deployment of mechanically coupled flexible electronics and enable their use in three major application areas: vital sign and environmental biosensing, intelligent sensing for complex industrial and motion systems, and high‐fidelity acoustic and vibration perception. This application‐oriented framework highlights how surface/interface design forms a complete innovation loop from failure analysis and structural regulation to device‐level reliability and practical implementation.

**TABLE 4:** Application‐oriented mapping of failure modes, surface/interfacial engineering strategies, key reliability metrics, and representative scenarios in mechanically coupled flexible electronics.

Failure mode and mechanism	Representative strategy	Key evaluation metrics	Device‐level reliability outcome	Application scenario	Remaining limitations
Surface contact instability caused by weak conformal adhesion, air gaps, or motion‐induced slipping	Self‐adhesive hydrogels, bioadhesive surfaces, microstructured adhesives	Adhesion strength, peel strength, contact stability, signal drift, motion artifact level	Reduced contact loss, lower signal drift, improved ECG/EMG fidelity	Skin ECG/EMG, underwater physiological monitoring, marine organism monitoring	Balance between strong adhesion, painless removal, residue‐free detachment, and skin compatibility
Surface contamination and liquid interference from sweat, water, oil, dust, and microorganisms	Superhydrophobic/oleophobic coatings, self‐cleaning surfaces, antibacterial/anti‐fouling layers	Water/oil contact angle, rolling angle, signal retention after liquid exposure, antibacterial performance	Improved stability under sweat, underwater, rain, oil, and contaminated environments	Diver monitoring, pipeline leakage detection, rain/wind sensing	Coating abrasion, long‐term durability, and breathability remain concerns
Environmental aging caused by dehydration, freezing, UV, heat, humidity, or oxidation	Organohydrogels, ionic liquids, anti‐freezing/moisture‐retention systems, UV‐blocking and encapsulation layers	Conductivity retention, adhesion retention, mechanical stability after aging, environmental cycling lifetime	Reduced long‐term output drift and improved service stability in harsh environments	Wearable monitoring, outdoor sensing, complex industrial environments	Long‐term coupled aging tests are still insufficient
Interfacial delamination or sliding in multilayer devices under bending, stretching, shear, or friction	Covalent grafting, diffusion‐induced cohesion, chemical crosslinking, adhesive interlayers, mechanical interlocking	Interfacial toughness, peel energy, peel strength, shear strength, cycling lifetime	Suppressed delamination, reduced electrode displacement, improved signal stability	ECG/EEG electrodes, EMG sensing, robotic gripping	Processing complexity and batch‐to‐batch reproducibility need improvement
Mechanical mismatch and stress concentration between rigid conductive layers and soft substrates	Buffer layers, gradient modulus structures, liquid‐metal conductors, elastomeric conductive networks	FEA stress distribution, failure strain, resistance drift under deformation, performance retention	Reduced stress concentration, delayed fracture, improved bending/stretching reliability	Motion monitoring, smart vehicle control, aircraft‐skin pressure sensing	Modulus optimization may affect sensitivity and response speed
Crack propagation and fatigue damage under repeated deformation	Crack‐arresting architectures, interpenetrating networks, self‐healing interfaces, percolating conductive networks	Crack length evolution, resistance variation, self‐healing efficiency, cyclic durability	Maintained conductive pathways and reduced fatigue‐induced failure	Wearable strain sensing, robotic climbing/lifting, joint monitoring	Trade‐off among self‐healing, mechanical strength, and conductivity
Electrical transport instability caused by interfacial separation, conductive‐pathway discontinuity, or capacitance fluctuation	Topological entanglement, interfacial percolation, conductive adhesive layers, stable electrode/dielectric bonding	Resistance/capacitance drift, hysteresis, response/recovery time, frequency‐domain consistency	Reduced hysteresis and contact‐resistance fluctuation, improved high‐frequency fidelity	Tire pressure monitoring, acoustic/vibration sensing, sound recognition	High‐frequency and long‐term dynamic testing protocols remain limited
Transfer‐ or assembly‐induced interfacial damage during fabrication or integration	On‐demand interfacial adhesion, damage‐free transfer printing, reversible bonding/debonding interfaces	Transfer yield, adhesion switching ratio, residual damage, post‐transfer electrical/mechanical retention	Improved integration reliability and reduced damage during device transfer	Dry transfer printing, detachable wearable devices, soft‐interface integration	Needs broader validation across materials, sizes, and device architectures

### 8.1 Life‐System Monitoring

**FIGURE 17:**
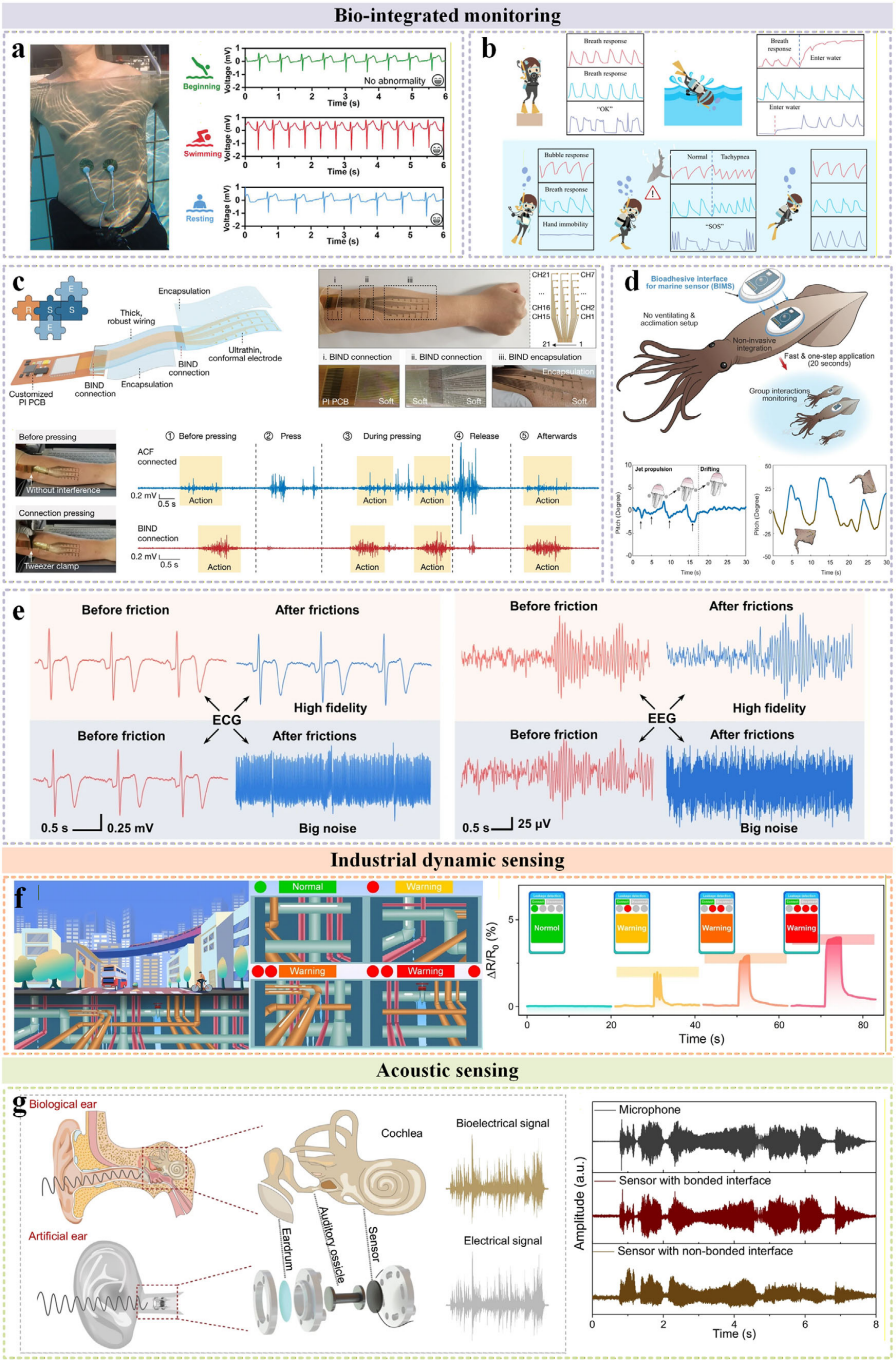
Bio‐integrated monitoring: (a) pDAM/Au/PDMS wearable ECG for underwater heart‐rate monitoring during swimming. Reproduced with permission [239]. Copyright 2020, Wiley‐VCH. (b) CNT@SiO_2_ sensor for underwater physiological monitoring of divers. Reproduced with permission [134]. Copyright 2024, Elsevier Ltd. (c) Bind interface‐assembled EMG with high signal fidelity. Reproduced with permission [247]. Copyright 2023, Springer Nature. (d) BIMS for epidermal adhesion on marine organisms and marine animal behavior monitoring. Reproduced under the terms of the Creative Commons Attribution 4.0 International License [169]. Copyright 2024, Springer Nature. (e) High‐fidelity ECG and EEG signals from diffusion‐induced cohesion Au‐WPU electrodes. Reproduced under the terms of the Creative Commons Attribution 4.0 International License [248]. Copyright 2024, Springer Nature. Industrial dynamic sensing: (f) TCGS sensor for evaluating leakage signals at different liquid flow rates. Reproduced under the terms of the Creative Commons Attribution 4.0 International License [8]. Copyright 2025, Springer Nature. Acoustic sensing: (g) The signal consistency of the interfacial adhesive sensor for sound detection. Reproduced under the terms of the Creative Commons Attribution 4.0 International License [237]. Copyright 2024, Springer Nature.

In life‐system monitoring, surface adhesion, anti‐fouling ability, and stable internal interfaces are directly related to signal fidelity, motion‐artifact suppression, and long‐term physiological recording. For mechanically coupled flexible electronics to conformally integrate with biological skin and continuously monitor physiological signals in a stable and interference‐free manner, their surfaces must exhibit strong adhesion and self‐cleaning capability, while maintaining conformal contact even under extreme conditions such as underwater or in sweat. Meanwhile, stable interfacial adhesion within multi‐layer devices is also required to prevent signal distortion, interfacial slippage, and structural damage during repeated physiological signal monitoring. A pDAM/Au/PDMS wearable electrocardiography (ECG) device enables continuous underwater heart‐rate monitoring, while maintaining good skin/ECG interfacial conformality and adhesion even underwater. As shown in Figure 17a, ECG can achieve T‐wave and R‐wave monitoring at different stages of swimming [239]. The sensor with o‐SiO_2_ coating on the surface can maintain long‐term underwater superhydrophobicity and monitor real‐time physiological signals of divers in air and underwater, including respiration, movement, and the process of divers diving and surfacing (Figure 17b) [134]. Compared to anisotropic conductive films, electromyography (EMG) assembled using a bind interface reduces the concentrated stress at the interface and has smaller MAs than the control electrode, as shown in Figure 17c. It has high signal fidelity and spatial resolution, and can stably test various EMG signals of the hand both on and underwater [247]. The Janus hydrophobic structure gel with asymmetric adhesion function realizes hydrophobicity after immersion and adhesion with PMMA through a double‐layer structure. The fabricated iontronic sensor can not only rely on the long‐chain alkyl to remove the interfacial water film and achieve underwater health monitoring, but also can build an underwater wireless communication system to achieve underwater rescue by relying on the sensor with strong adhesion and high mechanical strength [255]. A chitosan‐encapsulated MXene organohydrogel further illustrates the interface‐oriented design principle, as its conformal skin adhesion, repeatable attachment, and low skin‐electrode impedance supported stable EMG signal acquisition during repeated body movements [256]. Beyond human health monitoring, underwater sensors can also be applied to the physiological monitoring of marine organisms. Flexible electronic devices enable long‐term tracking of deep‐sea environments, animal behavior, and physiological ecology in a minimally invasive manner without disturbing natural behaviors. As shown in Figure 17d, BIMS can be quickly and non‐invasively integrated onto the skin surface of soft marine organisms. It can absorb surface seawater and firmly adhere to the surfaces of various marine organisms, simulating their respiration and movement. When adhered to fish fins, it can also analyze the kinematics of fish and identify specific animal movement patterns. They can conformally attach to the epidermis of diverse marine organisms and also enable tracking of group and community behaviors [169]. Moreover, flexible electronic devices with high interfacial toughness have broad applications in life‐system monitoring, including the detection of physiological activities, electrocardiograms, electroencephalograms, and joint disorders. An in situ grown CoS_2_/LM/VHB flexible sensor exhibits stable responses to pressure and bending, enabling the monitoring of motion, writing, and respiration [182]. Compared with conventional Au‐PDMS electrodes, stretchable Au‐WPU electrodes with strong interfacial adhesion enabled by interface diffusion‐induced cohesion can still stably record high‐fidelity ECG and EEG signals after repeated friction, as shown in Figure 17e [248]. The strain sensor fabricated by the synergistic strategy of mechanical interlocking via microstructure arrays and adhesive polymer is attached to the wrist. Due to its interfacial mechanism avoiding distortion of the Au electrode, it can accurately collect wrist motion data and quantify the pain level of tenosynovitis treatment [233]. Overall, these examples show that reliable life‐system monitoring depends not only on sensitivity, but also on stable skin/device contact, resistance to sweat or underwater interference, and robust interlayer adhesion. Surface and interfacial engineering therefore converts material‐level adhesion and wettability into device‐level improvements in ECG, EMG, respiration, motion, and biological monitoring.

### 8.2 Intelligent Sensing and Monitoring for Complex Industrial Equipment

In industrial and robotic scenarios, flexible devices are exposed to stronger shear, pressure, vibration, liquid leakage, rain, and large‐area deformation than in typical wearable applications. Mechanically coupled flexible electronics for extreme environments and dynamic loading require high environmental tolerance and structural reliability. These requirements underpin their applications in diverse industrial scenarios, including health monitoring of critical mechanical components, surface pressure mapping of large‐scale equipment such as aerospace vehicles, internal pressure sensing in industrial pipelines, tire pressure monitoring, and robotic motion sensing. TPU/CNTs/graphene sheets strain sensors (TCGS) designed with microporous and scorpion sensory structures can be applied in the field of industrial pipeline leakage due to surface dynamic superhydrophobic characteristics. As shown in Figure 17f, the sensor can evaluate the size of liquid leakage flow [8]. The superhydrophobic flexible sensor made of laser‐textured LIG/PDMS is attached to the surface of the umbrella, which can change the resistance of sliding raindrops with the refinement of the texture. The echo state network is built to enable the sensor to accurately predict water volume and wind speed [185]. When the pressure sensor with an interfacial topology is used for tire pressure monitoring, the high signal stability allows it to travel 2.6 kilometers in a car. When used for soft robot grasping objects, the signal amplitude does not change significantly, while the sensor without interfacial adhesion treatment shows a negative capacitance signal due to the air gap between the electrodes and the dielectric layer when bent [235]. A piezoelectric multidirectional bending sensor enabled by interfacial interlocking and penetration was conformally attached to the wrist for remote control of a smart vehicle. Under different bending states, the signals were wirelessly transmitted to direct multidirectional motion of the vehicle. The robust interfacial bonding within the multilayer piezoelectric device ensures reliable transmission of multidirectional signals [231]. An iontronic sensor based on a porous interfacial mechanical interlocking strategy, together with a TPU interfacial adhesion layer, prevents relative sliding between the electrode and dielectric layer. As a result, shear stress generated during robotic bottle grasping does not compromise long‐term stability [30]. The flexible pressure sensor that enhances the interfacial adhesive force through the design of the interfacial micropillar arrays can be integrated into the robot gripper to realize the grasping of heavy objects. Because the stable interface allows it to work stably under high shear force and pressure without causing interface delamination [26]. The stretchable device prepared by molecular chain entanglement and in‐situ polymerization network interpenetration has super interfacial force under electrical adhesion, and the stretchable device can lift weights, move objects, and realize robot climbing [29]. Intentionally designed interfacial mismatch can enable flexible mechanical devices with distinct functionalities, such as regulating the microstructural dimensions of electrodes and ion gels, while achieving controlled interfacial adhesion. By providing spatial allowance at the interface, simultaneous detection of positive and negative pressure can be realized. These devices are suitable for deployment on aircraft skin surfaces, meeting the requirements for wing pressure distribution testing in aviation wind tunnels, thereby satisfying aviation‐grade testing demands [10]. These cases indicate that industrial sensing requires interfaces that can withstand shear force, cyclic loading, liquid impact, and mechanical mismatch without delamination or signal distortion. Therefore, interfacial interlocking, adhesive layers, and topology‐regulated interfaces are especially important for maintaining stable pressure, leakage, robotic grasping, and aerospace monitoring signals.

### 8.3 Acoustic and Vibration Sensing

Sound vibration is an important carrier for mechanically coupled flexible electronics to demonstrate their high sensitivity and capture high‐frequency signals. Acoustic and vibration sensing places particularly high demands on interfacial stability because weak bonding or interfacial slipping can rapidly attenuate high‐frequency mechanical waves. Traditional microphones or accelerometers cannot conformally adhere to the surface of the measured object to detect sound signals. A strain sensor composed of Janus nanoparticle‐functionalized graphene sheets and styrene butadiene rubber can be conformally attached to the vocal tract region, enabling vowel classification and recognition through deep learning [257]. Pressure sensors with microcone‐planar topological entanglement at the interface can be applied in high‐frequency vibration sensing owing to their ultrafast response, such as sound detection. The sensor with interface adhesion can display stable vibration signals from 200 Hz to 12 kHz. As shown in Figure 17g, compared with non‐bonded sensors, interfacially topological‐bonded sensors exhibit superior frequency‐domain consistency in response to song‐generated sound waves [236]. Thus, for acoustic and vibration sensing, the key role of interfacial engineering is to preserve high‐frequency stress transfer and reduce phase delay, signal damping, and frequency‐domain distortion. This requirement distinguishes acoustic/vibration devices from low‐frequency strain or pressure sensors and highlights the need for strongly coupled, low‐loss interfaces.

## 9 Conclusions and Prospects

This review highlights the necessity of addressing surface and interfacial issues in mechanically coupled flexible electronics. From a fundamental physical perspective, it discusses surface contact mechanics and adhesion theory, the concepts of surface energy and wettability, and the thermomechanical coupling of flexible films. It also covers the adhesion theory of heterogeneous interfaces in multi‐layer flexible electronics, interfacial stress‐transfer mechanisms, failure modes, and the simulation of interfacial behaviors. The physical theory of surfaces and interfaces is the theoretical basis and prerequisite for studying the surface conformal adhesion mechanism, surface functionalization, and interfacial adhesion mechanism of mechanical electronic devices. It then outlines the surface and interfacial challenges in mechanically coupled flexible electronics, including self‐adhesion to target objects, surface self‐cleaning in complex environments, and long‐term anti‐aging performance. It further addresses key interfacial issues in multi‐layer devices, such as adhesion, mechanical matching, mechanical damage, and electrical transport. To address these issues, this review further discusses material design strategies, guided by surface and interfacial theories, in which intrinsic material design and innovation in flexible devices can directly resolve surface and interfacial problems. From the perspectives of chemical engineering and physical processing, this review further summarizes current strategies for improving the surface and interfacial performance of mechanically coupled flexible electronics. It compares the effects of different chemical surface modification, grafting, and physical adsorption approaches, as well as the mechanisms, advantages, and limitations of various physical processing techniques in enhancing surface and interfacial properties. From a cross‐scale perspective, this review summarizes and categorizes recent efforts that integrate material selection, chemical modification, physical processing, and emerging surface/interfacial structural designs to improve the surface and interfacial performance of mechanically coupled flexible electronics. These multiscale surface and interfacial architectonics include bioinspired structures, mechanical interlocking, adhesive interlayers, mechanical buffer layers, interfacial percolation and interpenetrating network designs, and homogeneity strategies, together with a summary of the surface and interfacial adhesion achieved by different strategies. Finally, this review surveys the diverse applications of mechanically coupled flexible electronics enabled by surface and interfacial strategies, including life‐system monitoring, intelligent sensing of industrial equipment, and acoustic/vibration detection.

As highlighted throughout this review, an increasing number of researchers have recognized that surface and interfacial issues are decisive for the reliability and environmental durability of mechanically coupled flexible electronics. Incorporating surface and interfacial engineering into device design and fabrication is therefore more critical than merely developing new functional materials and microstructures or pursuing higher sensitivity and linearity alone. Although multiscale surface and interfacial architectonics has demonstrated enormous potential, a series of fundamental challenges still hinder its transition from proof‐of‐concept studies to practical applications and future industrialization. Future research should therefore focus on the following key directions.

### 9.1 Standardized Characterization and Quantitative Metrics

Many researchers have not quantitatively evaluated the improvement in surface and interfacial bonding of mechanically coupled flexible electronics after optimization, and their analyses often remain limited to morphological characterization or macroscopic demonstrations. Although several reports have attempted to measure surface adhesion and interfacial bonding in cross‐scale surface/interface engineering, the corresponding testing and reporting protocols are still far from standardized. Reported m values are expressed in different forms, including stress, energy per unit area, and force per unit width, such as kPa, J m^−2^, and N m^−1^. These parameters are governed by distinct physical meanings and are strongly affected by specimen geometry, film width, film thickness, bending stiffness, peeling angle, loading rate, substrate modulus, and environmental conditions. As a result, direct comparison across different materials, interface designs, and device configurations remains difficult. For adhesion between two films, 180° peel tests are frequently used to evaluate peel strength or to estimate interfacial toughness when sufficient geometric information is available. Other studies adopt 90° peel, T‐peel, tensile detachment, lap‐shear, cyclic bending, or cyclic stretching tests, which impose different stress states and energy‐dissipation pathways at the interface. These differences further complicate quantitative comparison when the specimen shape, effective contact area, loading history, and normalization method are not clearly reported. In addition, adhesion theory, contact mechanics, wetting models, and specific surface/interfacial engineering strategies are often developed as separate topics. A more rigorous evaluation framework should first identify the relevant contact or fracture model for a given interface and then select appropriate experimental metrics accordingly. Therefore, future studies should move beyond qualitative demonstrations by combining standardized adhesion and interfacial toughness tests with explicit reporting of specimen geometry, loading configuration, peeling angle, loading rate, contact area, film thickness, substrate modulus, environmental conditions, cycle number, and normalization method. A clearer correspondence among testing configuration, physical model, and reported metric would make adhesion‐related data more traceable and would allow different surface and interfacial strategies to be compared on a more rigorous basis.

### 9.2 Scalable Manufacturing and Process Robustness

Surface functions such as self‐adhesion, self‐cleaning, and anti‐aging are generally governed by material innovation and structural design. The stability and reproducibility of material synthesis directly determine the effectiveness of surface functionalization in flexible devices, while the consistency of structural design and fabrication depends not only on process control but also on the batch‐to‐batch uniformity of materials. The synthesis of some materials, particularly elastomers and hydrogels, is highly sensitive to environmental humidity and temperature and often requires stringent processing conditions. Whether the resulting devices can maintain functional durability and performance consistency is therefore critical for their transition from laboratory prototypes to practical applications. In addition, the homogeneity and storage stability of composites formed by combining functional or conductive materials with flexible substrates are also key factors governing manufacturing consistency. The long‐term persistence of surface functionalization must likewise be considered, especially for chemically modified surfaces, where the durability of adhesion and resistance to aging remain essential concerns. Interfacial properties, including enhanced interfacial toughness, mechanical matching, and stable electrical transport, further depend on the controllability of interfacial engineering processes and techniques. Many emerging strategies are highly sensitive to factors such as prepolymer curing timing, the degree of percolating network formation, and the dimensions of mechanically interlocked structures. Thus, the reproducibility of hybrid chemical‐physical approaches and the robustness of processes are crucial in determining whether these devices can ultimately achieve practical application and industrialization. For practical manufacturing, interfacial reinforcement strategies should be reproducible across different batches and device sizes. Key parameters such as surface activation time, grafting density, curing degree, interlayer thickness, microstructure dimensions, and network formation should be quantitatively controlled. Meanwhile, scalable surface/interface engineering should also consider material sustainability, solvent selection, processing energy consumption, and device recyclability, especially for wearable and disposable sensing systems.

### 9.3 Robust Surface and Interfacial Design for Extreme Service Environments

Surface functionalization of mechanically coupled flexible electronics, particularly self‐adhesion, is primarily intended to achieve highly conformal contact with target surfaces, especially strong integration with biological skin, which directly determines device robustness under extreme service conditions. Simple adhesion tests on a few substrates with different properties are insufficient. They only reveal the bonding behavior of surface modification or material engineering with different substrates, but cannot truly reflect whether device adhesion to the target object changes under loading conditions such as extreme strain or high‐frequency vibration. Meanwhile, when applied across different fields, mechanically coupled flexible electronics must withstand various harsh environmental conditions, including humidity fluctuations, sweat during human health monitoring, saline environments during underwater operation, high temperatures generated by industrial equipment, UV radiation in outdoor applications, and exposure to varying pH levels or contaminants during pipeline pressure monitoring. More importantly, these environmental factors rarely act independently in real applications. Coupled aging tests that combine mechanical loading with sweat, water, humidity, UV irradiation, high temperature, low temperature, salt, oil, or contaminant exposure are needed to evaluate whether adhesion, interfacial toughness, conductivity, and signal stability can be maintained under realistic service conditions. This therefore requires a systematic design approach, rather than relying solely on the intrinsic environmental resistance of the device materials.

### 9.4 Quantitative Mapping from Surface and Interfacial Parameters to Application‐Level Performance

Enhancing surface and interfacial properties ultimately aims to ensure the long‐term stable operation and practical application of mechanically coupled flexible electronics. Currently, many studies are limited to demonstrating the novelty and uniqueness of design strategies or material‐level characterizations. Some researchers have begun to investigate how multiscale interfacial engineering affects device durability through long‐cycle comparative tests with devices lacking such strategies, which provides valuable insight into the relationship between interfacial parameters and device performance. Nevertheless, for mechanical sensors, these stability tests are often limited to a single frequency or pressure condition, whereas real applications involve far more complex operating environments. Whether the advantages of interfacial engineering persist under such conditions remains insufficiently explored yet critically important. In this context, in situ characterization will be particularly important for establishing the correlation between interfacial evolution and device performance. Techniques capable of tracking interfacial sliding, crack initiation, crack propagation, delamination, local strain redistribution, and conductive‐pathway degradation during operation can reveal how microscopic interfacial failure evolves into signal drift, hysteresis, MAs, or complete device failure. Therefore, it is necessary to establish a correlation mapping from interfacial parameters to device performance and ultimately to application‐level efficacy. Developing a comprehensive evaluation framework to quantitatively assess how improvements in surface and interfacial properties translate into practical performance gains will be a key challenge for future research.

## Author Contributions

**Wei Wu**: Writing – review and editing, supervision, project administration. **Qian Wang**: writing – original draft, writing – review and editing, investigation. **Fangfang Dai**: funding acquisition, resources.

## Funding

National Natural Science Foundation of China (NSFC, No. 52373252, 82502029), National High‐Level Talents Special Support Program, Sports Innovation Joint Funds of Natural Science Foundation of Hubei (2026AFC0994), the National Key R&D Program of China (No.2024YFC2417900, 2024YFC2706900).

## Conflicts of Interest

The authors declare no conflicts of interest.

## Data Availability

The authors have nothing to report.

## References

[1] C. Xu , J. Chen , Z. Zhu , et al., “Flexible Pressure Sensors in Human–Machine Interface Applications, ” Small 20, no. 15 (2023): 2306655, 10.1002/smll.202306655. 38009791

[2] K. Meng , X. Xiao , W. Wei , et al., “Wearable Pressure Sensors for Pulse Wave Monitoring, ” Advanced Materials 34, no. 21 (2022): 2109357, 10.1002/adma.202109357. 35044014

[3] K. Kang , J. Park , K. Kim , and K. J. Yu , “Recent Developments of Emerging Inorganic, Metal and Carbon‐Based Nanomaterials for Pressure Sensors and Their Healthcare Monitoring Applications, ” Nano Research 14, no. 9 (2021): 3096–3111, 10.1007/s12274-021-3490-0.

[4] C. Chen , J. L. Xu , Q. Wang , et al., “Biomimetic Multimodal Receptors for Comprehensive Artificial Human Somatosensory System, ” Advanced Materials 36, no. 21 (2024): 2313228, 10.1002/adma.202313228. 38330391

[5] S. Chun , W. Son , H. Kim , S. K. Lim , C. Pang , and C. Choi , “Self‐Powered Pressure‐ And Vibration‐Sensitive Tactile Sensors for Learning Technique‐Based Neural Finger Skin, ” Nano Letters 19, no. 5 (2019): 3305–3312, 10.1021/acs.nanolett.9b00922. 31021638

[6] H. Wang , Z. Liu , J. Ding , et al., “Downsized Sheath–Core Conducting Fibers for Weavable Superelastic Wires, Biosensors, Supercapacitors, and Strain Sensors, Supercapacitors, and Strain Sensors, ” Advanced Materials 28, no. 25 (2016): 4998–5007, 10.1002/adma.201600405. 27135200

[7] L. Yu and D. Liu , “Recent Progress in Tactile Sensing and Machine Learning for Texture Perception in Humanoid Robotics, ” Interdisciplinary Materials 4, no. 2 (2025): 235–248, 10.1002/idm2.12233.

[8] W. Zhou , Y. Du , Y. Chen , et al., “Bioinspired Ultrasensitive Flexible Strain Sensors for Real‐Time Wireless Detection of Liquid Leakage, ” Nano‐Micro Letters 17, no. 1 (2025): 68, 10.1007/s40820-024-01575-2. PMC11582251 39572445

[9] J. Li , Q. Dai , Z. Wang , et al., “Highly Robust and Self‐Adhesive Soft Strain Gauge via Interface Design Engineering, ” Advanced Materials 36, no. 38 (2024): 2406432, 10.1002/adma.202406432. 39081104

[10] J. Wang , X. Wei , J. Shi , et al., “High‐Resolution Flexible Iontronic Skins for both Negative and Positive Pressure Measurement in Room Temperature Wind Tunnel Applications, ” Nature Communications 15, no. 1 (2024): 7094, 10.1038/s41467-024-51415-5. PMC11330526 39153996

[11] C. Yang , D. Zhang , W. Wang , H. Zhang , and L. Zhou , “Multi‐Functional MXene/Helical Multi‐Walled Carbon Nanotubes Flexible Sensor for Tire Pressure Detection and Speech Recognition Enabled by Machine Learning, ” Chemical Engineering Journal 505 (2025): 159157, 10.1016/j.cej.2024.159157.

[12] J. H. Lee , K. Cho , and J. K. Kim , “Age of Flexible Electronics: Emerging Trends in Soft Multifunctional Sensors, ” Advanced Materials 36, no. 16 (2024): 2310505, 10.1002/adma.202310505. 38258951

[13] A. Maji , C. Kuila , N. C. Murmu , and T. Kuila , “Stretch, Sense, and Innovate: Advances in Next‐Generation Strain Sensors, ” Composites Part B: Engineering 306 (2025): 112749, 10.1016/j.compositesb.2025.112749.

[14] J. Chen , Y. Zhu , X. Chang , et al., “Recent Progress in Essential Functions of Soft Electronic Skin, ” Advanced Functional Materials 31, no. 42 (2021): 2104686, 10.1002/adfm.202104686.

[15] J. Shi , Y. Dai , Y. Cheng , et al., “Embedment of Sensing Elements for Robust, Highly Sensitive, and Cross‐talk–free Iontronic Skins for Robotics Applications, ” Science Advances 9, no. 9 (2023): adf8831, 10.1126/sciadv.adf8831. PMC9984179 36867698

[16] J. You , M. Lu , L. Dazhen , et al., “Anti‐Motion Artifacts Iontronic Sensor for Long‐Term Accurate Fingertip Pulse Monitoring, ” Advanced Science 12, no. 15 (2025): 2414425, 10.1002/advs.202414425. 39985252 PMC12005763

[17] B. Park , J. Kim , D. Kang , et al., “Dramatically Enhanced Mechanosensitivity and Signal‐to‐Noise Ratio of Nanoscale Crack‐Based Sensors: Effect of Crack Depth, ” Advanced Materials 28, no. 37 (2016): 8130–8137, 10.1002/adma.201602425. 27396592

[18] J. Kang , J. Mun , Y. Zheng , et al., “Tough‐Interface‐Enabled Stretchable Electronics Using Non‐Stretchable Polymer Semiconductors and Conductors, ” Nature Nanotechnology 17, no. 12 (2022): 1265–1271, 10.1038/s41565-022-01246-6. 36357793

[19] J. Ma , Q. Liu , L. Yang , et al., “A Flexible, Highly Accurate, and Stable Pressure Sensor with Anti‐Interference from Temperature, Sweat, and Humidity, ” Advanced Materials Interfaces 10, no. 6 (2023): 2201528, 10.1002/admi.202201528.

[20] H. Kou , Q. Tan , Y. Wang , G. Zhang , S. Su , and J. Xiong , “A Wireless Slot‐Antenna Integrated Temperature‐Pressure‐Humidity Sensor Loaded with CSRR for Harsh‐Environment Applications, ” Sensors and Actuators B: Chemical 311 (2020): 127907, 10.1016/j.snb.2020.127907.

[21] S. Huang , Y. Liu , Y. Zhao , Z. Ren , and C. F. Guo , “Flexible Electronics: Stretchable Electrodes and Their Future, ” Advanced Functional Materials 29, no. 6 (2018): 1805924, 10.1002/adfm.201805924.

[22] Y. Khan , A. Thielens , S. Muin , J. Ting , C. Baumbauer , and A. C. Arias , “A New Frontier of Printed Electronics: Flexible Hybrid Electronics: Advanced Materials, Flexible Hybrid Electronics, ” Advanced Materials 32, no. 15 (2019): 1905279, 10.1002/adma.201905279. 31742812

[23] W. Babatain , C. Park , H. Ishii , and N. Gershenfeld , “Laser‐Enabled Fabrication of Flexible Printed Electronics with Integrated Functional Devices, ” Advanced Science 12, no. 20 (2025): 2415272, 10.1002/advs.202415272. 40040310 PMC12120706

[24] H. Hu , D. Wang , H. Tian , et al., “Bioinspired Hierarchical Structures for Contact‐Sensible Adhesives, ” Advanced Functional Materials 32, no. 8 (2022): 2109076, 10.1002/adfm.202109076.

[25] M. Li , Z. Liu , Y. Hu , R. Li , and Y. Cao , “A Hydrophobic Eutectogel with Excellent Underwater Self‐Adhesion, Self‐Healing, Transparency, Stretchability, Ionic Conductivity, and Fully Recyclability, ” Chemical Engineering Journal 472 (2023): 145177, 10.1016/j.cej.2023.145177.

[26] X. Xia , X. Chen , J. Shi , et al., “Micropillar‐Enabled Tough Adhesion and Enhanced Sensing, Matter, ” Matter 8, no. 10 (2025): 102221, 10.1016/j.matt.2025.102221.

[27] J. Zhang , Z. Ma , M. Li , M. Lou , H. Wang , and L. Jia , “Development of Ultrathin, Breathable, Waterproof, and Durable Nanonet‐Supported Ionogel Sensors for Electrophysiological Monitoring, ” Advanced Functional Materials 35, no. 8 (2025): 2415694, 10.1002/adfm.202415694.

[28] K. Zhao , Y. Zhao , J. Wang , and C. Ye , “Liquid Metal Nanoparticle‐Based Multifunctional Printable Electronics Enabled by Polymerizable Deep Eutectic Solvents, ” Advanced Functional Materials 35, no. 24 (2025): 2424490, 10.1002/adfm.202424490.

[29] Y. Zheng , H. Ning , B. Zhao , et al., “Molecular Chain Interpenetration–Enabled High Interfacial Compatibility of Ionic and Electronic Conductors for Stretchable Ionic Devices, ” Advanced Materials 37, no. 16 (2025): 2417175, 10.1002/adma.202417175. 40072337

[30] B. Zhu , J. Guo , W. Li , et al., “Integrated Electromechanical Structure for Iontronic Pressure Sensors with Linear High‐Sensitivity Response and Robust Sensing Stability, ” Advanced Functional Materials 34, no. 42 (2024): 2406762, 10.1002/adfm.202406762.

[31] Y. Wang , H. Hu , J. Shao , and Y. Ding , “Fabrication of Well‐Defined Mushroom‐Shaped Structures for Biomimetic Dry Adhesive by Conventional Photolithography and Molding, ” ACS Applied Materials & Interfaces 6, no. 4 (2014): 2213–2218, 10.1021/am4052393. 24527733

[32] T. Zhou , B. Ruan , J. Che , H. Li , X. Chen , and Z. Jiang , “Gecko‐Inspired Biomimetic Surfaces with Annular Wedge Structures Fabricated by Ultraprecision Machining and Replica Molding, ” ACS Omega 6, no. 10 (2021): 6757–6765, 10.1021/acsomega.0c05804. 33748589 PMC7970495

[33] F. Rusu , M. Pustan , C. Bîrleanu , R. Müller , R. Voicu , and A. Baracu , “Analysis of the Surface Effects on Adhesion in MEMS Structures, ” Applied Surface Science 358 (2015): 634–640, 10.1016/j.apsusc.2015.09.052.

[34] W. Yang , W. Ding , M. Liu , J. Yang , and M. Li , “A Theoretical Model of a Flexible Capacitive Pressure Sensor with Microstructured Electrodes for Highly Sensitive Electronic Skin, ” Journal of Physics D: Applied Physics 55, no. 9 (2022): 094001, 10.1088/1361-6463/ac34a9.

[35] Y. P. Zhao , L. S. Wang , and T. X. Yu , “Mechanics of Adhesion in MEMS—A Review, ” Journal of Adhesion Science and Technology 17, no. 4 (2003): 519–546, 10.1163/15685610360554393.

[36] A. Hariri , J. Zu , and R. Ben Mrad , “Modeling of Wet Stiction in Microelectromechanical Systems (MEMS), ” Journal of Microelectromechanical Systems 16, no. 5 (2007): 1276–1285, 10.1109/JMEMS.2007.904349.

[37] Y. Cao , D. Yang , and W. Soboyejoy , “Nanoindentation Method for Determining the Initial Contact and Adhesion Characteristics of Soft Polydimethylsiloxane, ” Journal of Materials Research 20, no. 8 (2005): 2004–2011, 10.1557/JMR.2005.0256.

[38] M. Joulaei , M. Kolahdoozan , M. Salehi , M. Zadsar , and M. Vahabi , “Comparison of the Adhesion Forces in Single and Double‐Layer Coatings on the MEMS Surfaces by JKR and DMT Models, ” Surface and Interface Analysis 52, no. 1‐2 (2020): 34–41, 10.1002/sia.6719.

[39] W. Zheng and Z. Ya‐Pu , “Adhesion Elastic Contact and Hysteresis Effect, ” Chinese Physics 13, no. 8 (2004): 1320–1325, 10.1088/1009-1963/13/8/024.

[40] X. Xue and A. A. Polycarpou , “An Improved Meniscus Surface Model for Contacting Rough Surfaces, ” Journal of Colloid and Interface Science 311, no. 1 (2007): 203–211, 10.1016/j.jcis.2007.02.038. 17379237

[41] S. Abetkovskaia , S. Chizhik , P. Nazaruk , J. M. Stelmachowski , and Z. Rymuza , “Complex Approach to Investigate Mechanical and Tribological Characteristics of Microcontacting Objects for MEMS, ” Tribology‐Materials Surfaces & Interfaces 7, no. 2 (2013): 83–86, 10.1179/1751584x13Y.0000000035.

[42] J. He , R. Zhou , Y. Zhang , et al., “Strain‐Insensitive Self‐Powered Tactile Sensor Arrays Based on Intrinsically Stretchable and Patternable Ultrathin Conformal Wrinkled Graphene‐Elastomer Composite, ” Advanced Functional Materials 32, no. 10 (2022): 2107281, 10.1002/adfm.202107281.

[43] M. Li and B. Sun , “Post‐Buckling Behaviors of Thin‐Film Soft‐Substrate Bilayers with Finite‐Thickness Substrate, ” Scientific Reports 12, no. 1 (2022): 4074, 10.1038/s41598-022-08136-w. 35260786 PMC8904586

[44] H. Wang , J. Wang , D. Chen , et al., “Robust Tattoo Electrode Prepared by Paper‐Assisted Water Transfer Printing for Wearable Health Monitoring, ” IEEE Sensors Journal 22, no. 5 (2022): 3817–3827, 10.1109/JSEN.2022.3141457.

[45] S. C. L. Fischer , S. Boyadzhieva , R. Hensel , K. Kruttwig , and E. Arzt , “Adhesion and Relaxation of a Soft Elastomer on Surfaces with Skin like Roughness, ” Journal of the Mechanical Behavior of Biomedical Materials 80 (2018): 303–310, 10.1016/j.jmbbm.2018.01.032. 29459289 PMC7617216

[46] X. Wang , Y. Liu , H. Cheng , and X. Ouyang , “Surface Wettability for Skin‐Interfaced Sensors and Devices, ” Advanced Functional Materials 32, no. 27 (2022): 2200260, 10.1002/adfm.202200260. 36176721 PMC9514151

[47] A. Kim , S. Mitra , S. Shyam , B. Zhao , and S. K. Mitra , “Flexible Hydrogels Connecting Adhesion and Wetting, ” Soft Matter 20, no. 28 (2024): 5516–5526, 10.1039/d4sm00022f. 38651874

[48] A. Malik and B. Kandasubramanian , “Flexible Polymeric Substrates for Electronic Applications, ” Polymer Reviews 58, no. 4 (2018): 630–667, 10.1080/15583724.2018.1473424.

[49] M. Lu , J. You , M. Gao , et al., “Interfacial Adhesion in Flexible Electronics: Materials, Structures and Applications, Coordination Chemistry Reviews, ” Coordination Chemistry Reviews 523 (2025): 216278, 10.1016/j.ccr.2024.216278.

[50] S. Ji and X. Chen , “Enhancing the Interfacial Binding Strength between Modular Stretchable Electronic Components, ” National Science Review 10, no. 1 (2023): nwac172, 10.1093/nsr/nwac172. 36684519 PMC9843131

[51] P. G. de Gennes , “Wetting: Statics and Dynamics, ” Reviews of Modern Physics 57, no. 3 (1985): 827–863, 10.1103/revmodphys.57.827.

[52] J. Lin , X. Cai , Z. Liu , et al., “Anti‐Liquid‐Interfering and Bacterially Antiadhesive Strategy for Highly Stretchable and Ultrasensitive Strain Sensors Based on Cassie‐Baxter Wetting State, ” Advanced Functional Materials 30, no. 23 (2020): 2000398, 10.1002/adfm.202000398.

[53] D. K. Owens , “Some Thermodynamic Aspects of Polymer Adhesion, ” Journal of Applied Polymer Science 14, no. 7 (1970): 1725–1730, 10.1002/app.1970.070140706.

[54] A. Amirfazli , “On Thermodynamics of Thin Films: the Mechanical Equilibrium Condition and Contact Angles, ” The Journal of Adhesion 80, no. 10‐11 (2004): 1003–1016, 10.1080/00218460490509345.

[55] S. H. Ko , “Low Temperature Thermal Engineering of Nanoparticle Ink for Flexible Electronics Applications, ” Semiconductor Science and Technology 31, no. 7 (2016): 073003, 10.1088/0268-1242/31/7/073003.

[56] A. I. Rusanov , “On the Thermodynamics of Thin Films. The Frumkin Equation, ” Colloid Journal 81, no. 6 (2019): 741–746, 10.1134/S1061933x19060152.

[57] K. L. Johnson , K. Kendall , and A. D. Roberts , “Surface Energy and the Contact of Elastic Solids, ” Proceedings of the Royal Society of London A Mathematical and Physical Sciences 324, no. 1558 (1971): 301–313, 10.1098/rspa.1971.0141.

[58] B. V. Derjaguin , V. M. Muller , and Y. P. Toporov , “Effect of Contact Deformations on the Adhesion of Particles, ” Journal of Colloid and Interface Science 53, no. 2 (1975): 314–326, 10.1016/0021-9797(75)90018-1.

[59] G. Xie , J. Ding , S. Liu , W. Xue , and J. Luo , “Interfacial Properties for Real Rough MEMS/NEMS Surfaces by Incorporating the Electrostatic and Casimir Effects–a Theoretical Study, ” Surface and Interface Analysis 41, no. 4 (2009): 338–346, 10.1002/sia.3029.

[60] X. Shi , L. Zhu , H. Yu , et al., “Interfacial Click Chemistry Enabled Strong Adhesion toward Ultra‐Durable Crack‐Based Flexible Strain Sensors, ” Advanced Functional Materials 33, no. 27 (2023): 2301036, 10.1002/adfm.202301036.

[61] K. Tripathy and M. Bhattacharjee , “Stretching Mode Deformation Analysis for an Elastomeric Encapsulation‐Assisted Stable Flexible Electronic Substrate, ” Flexible and Printed Electronics 8, no. 2 (2023): 025002, 10.1088/2058-8585/acca30.

[62] M. Sawan , H. Reda , N. Saad , S. Bin , and G. Nassar , “Formalism of Biological Tissues/Nanowire Sensor Interface Behavior, ” Wseas Transactions On Biology and Biomedicine 17 (2020): 27–31, 10.37394/23208.2020.17.4.

[63] N. Hou , Y. Zhao , R. Jiang , et al., “Flexible Piezoresistive Sensor Based on Surface Modified Dishcloth Fibers for Wearable Electronics Device, ” Colloids and Surfaces a: Physicochemical and Engineering Aspects 650 (2022): 129638, 10.1016/j.colsurfa.2022.129638.

[64] Q. Liu , Y. Zhang , X. Sun , et al., “All Textile‐Based Robust Pressure Sensors for Smart Garments, ” Chemical Engineering Journal 454 (2023): 140302, 10.1016/j.cej.2022.140302.

[65] Y. Wang , W. Qin , M. Yang , et al., “High Linearity, Low Hysteresis Ti_3_C_2_Tx MXene/AgNW/Liquid Metal Self‐Healing Strain Sensor Modulated by Dynamic Disulfide and Hydrogen Bonds, ” Advanced Functional Materials 33, no. 37 (2023): 2301587, 10.1002/adfm.202301587.

[66] H. Chen , X. Feng , Y. Huang , Y. Huang , and J. A. Rogers , “Experiments and Viscoelastic Analysis of Peel Test with Patterned Strips for Applications to Transfer Printing, ” Journal of the Mechanics and Physics of Solids 61, no. 8 (2013): 1737–1752, 10.1016/j.jmps.2013.04.001.

[67] L. Dai , Y. Huang , H. Chen , X. Feng , and D. Fang , “Transition among Failure Modes of the Bending System with a Stiff Film on a Soft Substrate, ” Applied Physics Letters 106, no. 2 (2015): 21905, 10.1063/1.4905697.

[68] H. Chen , X. Feng , and Y. Chen , “Slip Zone Model for Interfacial Failures of Stiff Film/Soft Substrate Composite System in Flexible Electronics, ” Mechanics of Materials 79 (2014): 35–44, 10.1016/j.mechmat.2014.08.007.

[69] Y. Huang , X. Feng , and B. Qu , “Slippage Toughness Measurement of Soft Interface between Stiff Thin Films and Elastomeric Substrate, ” Review of Scientific Instruments 82, no. 10 (2011): 104704, 10.1063/1.3646461. 22047314

[70] J. W. Hutchinson and Z. Suo , “Mixed‐Mode Cracking in Layered Materials, ” Advances in Applied Mechanics 29 (1992): 63–191, 10.1016/s0065-2156(08)70164-9.

[71] Y. Huang , J. Yuan , Y. Zhang , and X. Feng , “Interfacial Delamination of Inorganic Films on Viscoelastic Substrates, ” Journal of Applied Mechanics 83, no. 10 (2016): 101005, 10.1115/1.4034116.

[72] H. Yin , Z. Peng , and S. Chen , “The Peeling Behavior of a Heterogeneous Elastic Film on a Rigid Substrate, ” International Journal of Solids and Structures 285 (2023): 112529, 10.1016/j.ijsolstr.2023.112529.

[73] D. S. Dugdale , “Yielding of Steel Sheets Containing Slits, ” Journal of the Mechanics and Physics of Solids 8, no. 2 (1960): 100–104, 10.1016/0022-5096(60)90013-2.

[74] W. Jian , H. Yin , Y. Chen , and X. Feng , “Competing Behavior of Interface Delamination and Wafer Cracking During Peeling Film from Ultra‐Thin Wafer, ” International Journal of Solids and Structures 305 (2024): 113058, 10.1016/j.ijsolstr.2024.113058.

[75] H. Yin , Y. Yao , Y. Yang , Z. Peng , and S. Chen , “Interfacial Competitive Debonding of a Bilayer Elastic Film on a Rigid Substrate, ” Journal of Applied Mechanics 89, no. 1 (2022): 11003, 10.1115/1.4052151.

[76] Y. Kubo , H. Tanaka , Y. Saito , and A. Mizoguchi , “Fabrication of a Bilayer Structure of Cu and Polyimide to Realize Circuit Microminiaturization and High Interfacial Adhesion in Flexible Electronic Devices, ” ACS Applied Materials & Interfaces 10, no. 51 (2018): 44589–44602, 10.1021/acsami.8b15835. 30507162

[77] Y. Zhou , R. Cui , Y. Song , X. Fan , and J. Zhu , “Interfacial Stress Analysis of PVD Thin Film Sensor Based on Finite Element, ” Journal of Physics: Conference Series 2350, no. 1 (2022): 012005, 10.1088/1742-6596/2350/1/012005.

[78] J. Kim and L. P. Chamorro , “Coupled Mechanics in Skin‐Interfaced Electronics via Computer Vision Methods, ” Soft Science 4, no. 1 (2024): 12, 10.20517/ss.2023.50.

[79] A. Kleinbichler , M. Bartosik , B. Völker , and M. J. Cordill , “Thin Film Adhesion of Flexible Electronics Influenced by Interlayers, ” Advanced Engineering Materials 19, no. 4 (2017): 1600665, 10.1002/adem.201600665.

[80] X. Wang , G. Zhang , H. Yang , et al., “Quantitative Characterization of Interface Stress Using a Nanoindentation Technique for High Performance Flexible Electronics, ” Journal of Materials Chemistry C 8, no. 35 (2020): 12155–12163, 10.1039/d0tc02834g.

[81] Y. Shi , Y. Tian , Y. Guan , et al., “All‐Polymer Piezoelectric Elastomer with High Stretchability, Low Hysteresis, Self‐Adhesion, and UV‐Blocking as Flexible Sensor, ” ACS Applied Materials & Interfaces 15, no. 36 (2023): 43003–43015, 10.1021/acsami.3c09065. 37650377

[82] Y. Zheng , T. Cui , J. Wang , H. Ge , and Z. Gui , “Unveiling Innovative Design of Customizable Adhesive Flexible Devices from Self‐Healing Ionogels with Robust Adhesion and Sustainability, ” Chemical Engineering Journal 471 (2023): 144617, 10.1016/j.cej.2023.144617.

[83] H. Liu , W. Wang , H. Xiang , et al., “Paper‐Based Flexible Strain and Pressure Sensor with Enhanced Mechanical Strength and Super‐Hydrophobicity That Can Work under Water, ” Journal of Materials Chemistry C 10, no. 10 (2022): 3908–3918, 10.1039/D1TC04697G.

[84] H. Xiang , Z. Li , W. Wang , et al., “Enabling a Paper‐Based Flexible Sensor to Work under Water with Exceptional Long‐Term Durability through Biomimetic Reassembling of Nanomaterials from Natural Wood, ” ACS Sustainable Chemistry & Engineering 11, no. 23 (2023): 8667–8674, 10.1021/acssuschemeng.3c01870.

[85] K. Chen , Z. Yuan , S. Dai , J. Zhou , and K. Yu , “Preparation and Performance of Self‐Cleaning Photothermal‐Induced Self‐Healing Flexible Sensors, ” Composites Science and Technology 242 (2023): 110194, 10.1016/j.compscitech.2023.110194.

[86] C. Hu , Q. Sun , L. Xue , et al., “Bioinspired Flexible Wearable Sensor with High Self‐Cleaning and Antibacterial Performance for Human Motion Sensing, ” ACS Applied Bio Materials 6, no. 12 (2023): 5768–5775, 10.1021/acsabm.3c00873. 38029407

[87] M. Li , W. Liu , Z. Yin , et al., “Facile Fabrication of Superhydrophobic and Photocatalytic Self‐Cleaning Flexible Strain Sensor Membrane for Human Motion, ” Sensors and Actuators a: Physical 363 (2023): 114750, 10.1016/j.sna.2023.114750.

[88] Q. Song , K. Wang , and G. Zhao , “Self‐Adhesive, Conductive, and Antibacterial Hydrogel Nanofiber Composite as a Flexible Strain Sensor, ” ACS Applied Electronic Materials 5, no. 12 (2023): 6947–6954, 10.1021/acsaelm.3c01359.

[89] Z. Zhang , Q. Xie , G. Zhang , C. Ma , and G. Zhang , “Surface‐Enriched Amphiphilic Polysiloxane Coating with Superior Antifouling Ability and Strong Substrate Adhesion, ” ACS Applied Polymer Materials 5, no. 5 (2023): 3524–3533, 10.1021/acsapm.3c00199.

[90] Z. Wang and M. Huang , “From Grape Seed Extracts to Extremely Stable Strain Sensors with Freezing Tolerance, Drying Resistance and Anti‐Oxidation Properties, ” Materials Today Communications 33 (2022): 104551, 10.1016/j.mtcomm.2022.104551.

[91] L.‐Y. Zeng , X. C. Wang , Y. Wen , et al., “Anti‐Freezing Dual‐Network Hydrogels with High‐Strength, Self‐Adhesive and Strain‐Sensitive for Flexible Sensors, ” Carbohydrate Polymers 300 (2023): 120229, 10.1016/j.carbpol.2022.120229. 36372501

[92] G. Sun , P. Wang , Y. Jiang , et al., “Bioinspired Flexible, Breathable, Waterproof and Self‐Cleaning Iontronic Tactile Sensors for Special Underwater Sensing Applications, ” Nano Energy 110 (2023): 108367, 10.1016/j.nanoen.2023.108367.

[93] Y. Yang , H. Sun , C. Shi , Y. Liu , Y. Zhu , and Y. Song , “Self‐Healing Hydrogel with Multiple Adhesion as Sensors for Winter Sports, ” Journal of Colloid and Interface Science 629 (2023): 1021–1031, 10.1016/j.jcis.2022.08.167. 36152615

[94] W. Wang , H. Zhou , Z. Xu , Z. Li , L. Zhang , and P. Wan , “Flexible Conformally Bioadhesive MXene Hydrogel Electronics for Machine Learning‐Facilitated Human‐Interactive Sensing, ” Advanced Materials 36, no. 31 (2024): 2401035, 10.1002/adma.202401035. 38552161

[95] H. Yuk , T. Zhang , G. A. Parada , X. Liu , and X. Zhao , “Skin‐inspired Hydrogel–elastomer Hybrids with Robust Interfaces and Functional Microstructures, ” Nature Communications 7, no. 1 (2016): 12028, 10.1038/ncomms12028. PMC4931236 27345380

[96] X. Li , W. Wang , L. Wu , et al., “Wearable, Self‐Cleaning, Wireless Integrated Tactile Sensory System with Superior Sensitivity, ” Sensors and Actuators a: Physical 331 (2021): 113027, 10.1016/j.sna.2021.113027.

[97] Q. Lin , J. Huang , J. Yang , et al., “Highly Sensitive Flexible Iontronic Pressure Sensor for Fingertip Pulse Monitoring, ” Advanced Healthcare Materials 9, no. 17 (2020): 2001023, 10.1002/adhm.202001023. 32729260

[98] H. Cheng , H. Huang , W. Chen , and S. Lu , “Hygro‐Thermo‐Mechanical Behavior of Adhesive‐Based Flexible Chip‐On‐Flex Packaging, ” Journal of Electronic Materials 44, no. 4 (2015): 1220–1237, 10.1007/s11664-015-3627-6.

[99] S. Wang , J. Jin , D. Mei , and Y. Wang , “Conformal Theoretical Modeling of Arbitrary Shape Flexible Electronic Sensors Mounted onto General Curved Surface Substrates, ” Journal of Applied Mechanics 90, no. 11 (2023): 111007, 10.1115/1.4062905.

[100] H. Hu , X. Guo , Y. Zhang , et al., “Elasto‐Plastic Design of Ultrathin Interlayer for Enhancing Strain Tolerance of Flexible Electronics, ” ACS Nano 17, no. 4 (2023): 3921–3930, 10.1021/acsnano.2c12269. 36762695

[101] Y. Shao , J. Yan , Y. Zhi , et al., “A Universal Packaging Substrate for Mechanically Stable Assembly of Stretchable Electronics, ” Nature Communications 15, no. 1 (2024): 6106, 10.1038/s41467-024-50494-8. PMC11271615 39030235

[102] J. Lee , Y. Lee , J. Seol , Y. Joo , and B. Kim , “Vertical Pattern of Interconnects to Bypass High Strain near a Hard Die on a Flexible Substrate under Mechanical Bending, ” Electronic Materials Letters 20, no. 2 (2024): 122–130, 10.1007/s13391-023-00444-1.

[103] N. Zheng , J. Liu , G. Wang , et al., “Robust UV/Moisture Dual Curable PDMS‐Microcapsule‐Silica Functional Material for Self‐Healing, Antifouling, and Antibacterial Applications, ” Nano Research 16, no. 5 (2023): 7810–7819, 10.1007/s12274-023-5563-8.

[104] M. Qi , R. Yang , Z. Wang , et al., “Bioinspired Self‐Healing Soft Electronics, ” Advanced Functional Materials 33, no. 17 (2023): 2214479, 10.1002/adfm.202214479.

[105] Z. P. Zhang , M. Z. Rong , and M. Q. Zhang , “Self‐Healable Functional Polymers and Polymer‐Based Composites, ” Progress in Polymer Science 144 (2023): 101724, 10.1016/j.progpolymsci.2023.101724.

[106] Q.‐Q. Fu , T. Zhou , Y. Chen , et al., “Homogeneity Permitted Robust Connection for Additive Manufacturing Stretchable Electronics, ” ACS Applied Materials & Interfaces 12, no. 38 (2020): 43152–43159, 10.1021/acsami.0c13071. 32865957

[107] H. Ma , W. Li , Q. Zhu , et al., “Large Area and Flexible Flexion Sensing Matrix for Detection of Strain Distribution in Bendable and Curved Surface, ” ACS Sensors 10, no. 7 (2025): 4896–4905, 10.1021/acssensors.5c00153. 40211439

[108] G. Yang , X. Zheng , J. Li , et al., “Schottky Effect‐Enabled High Unit‐Area Capacitive Interface for Flexible Pressure Sensors, ” Advanced Functional Materials 34, no. 28 (2024): 2401415, 10.1002/adfm.202401415.

[109] H. Yang , S. Ji , I. Chaturvedi , et al., “Adhesive Biocomposite Electrodes on Sweaty Skin for Long‐Term Continuous Electrophysiological Monitoring, ” ACS Materials Letters 2, no. 5 (2020): 478–484, 10.1021/acsmaterialslett.0c00085.

[110] Z. Liu , J. Liu , S. He , X. Wu , X. Wei , and W. Shao , “Self‐Healing and Self‐Adhesion Conductive Hydrogels Reinforced by Carboxylated Carbon Nanotubes for High‐Performance Wearable Strain Sensors, ” ACS Applied Nano Materials 7, no. 7 (2024): 7653–7662, 10.1021/acsanm.4c00369.

[111] A. Roy , K. Manna , P. G. Ray , S. Dhara , and S. Pal , “Β‐Cyclodextrin‐Based Ultrahigh Stretchable, Flexible, Electro‐ And Pressure‐Responsive, Adhesive, Transparent Hydrogel as Motion Sensor, ” ACS Applied Materials & Interfaces 14, no. 15 (2022): 17065–17080, 10.1021/acsami.2c00101. 35394754

[112] X. Xu , P. Jiang , D. Liu , et al., “3D Printable Ionic Conductive Hydrogels with Super Stretch and Self‐Adhesion Performances for Flexible Sensors, ” Giant 17 (2024): 100237, 10.1016/j.giant.2024.100237.

[113] H. Wang , B. Liu , D. Chen , et al., “Low Hysteresis Zwitterionic Supramolecular Polymer Ion‐Conductive Elastomers with Anti‐Freezing Properties, High Stretchability, and Self‐Adhesion for Flexible Electronic Devices, ” Materials Horizons 11, no. 11 (2024): 2628–2642, 10.1039/d4mh00174e. 38501271

[114] R. Xue , N. Zhou , S. Yin , Z. Qian , Z. Dai , and Y. Xiong , “All‐Polymer Dynamical Ionogel‐Like Materials with Benzyl‐Mediated Ultra‐Strong Adhesion for Flexible Sensor Application, ” Chemical Engineering Journal 465 (2023): 143072, 10.1016/j.cej.2023.143072.

[115] T. Ke , L. Zhao , X. Fan , and H. Gu , “Rapid Self‐Healing, Self‐Adhesive, Anti‐Freezing, Moisturizing, Antibacterial and Multi‐Stimuli‐Responsive PVA/Starch/Tea Polyphenol‐Based Composite Conductive Organohydrogel as Flexible Strain Sensor, ” Journal of Materials Science & Technology 135 (2023): 199–212, 10.1016/j.jmst.2022.06.032.

[116] J. Wang , T. Dai , H. Wu , M. Ye , G. Yuan , and H. Jia , “Tannic Acid‐Fe^3+^ Activated Rapid Polymerization of Ionic Conductive Hydrogels with High Mechanical Properties, Self‐Healing, and Self‐Adhesion for Flexible Wearable Sensors, ” Composites Science and Technology 221 (2022): 109345, 10.1016/j.compscitech.2022.109345.

[117] H. Zhou , J. Lai , X. Jin , et al., “Intrinsically Adhesive, Highly Sensitive and Temperature Tolerant Flexible Sensors Based on Double Network Organohydrogels, ” Chemical Engineering Journal 413 (2021): 127544, 10.1016/j.cej.2020.127544.

[118] L. Xiong , S. Jin , F. Zhang , et al., “Bioinspired Fabrication of Self‐Recovery, Adhesive, and Flexible Conductive Hydrogel Sensor Driven by Dynamic Borate Ester Bonds and Tannic Acid‐Mediated Noncovalent Network, ” European Polymer Journal 180 (2022): 111636, 10.1016/j.eurpolymj.2022.111636.

[119] H. Zhou , Z. Jin , Y. Gao , et al., “Thermoresponsive, Magnetic, Adhesive and Conductive Nanocomposite Hydrogels for Wireless and Non‐Contact Flexible Sensors, ” Colloids and Surfaces a: Physicochemical and Engineering Aspects 636 (2022): 128113, 10.1016/j.colsurfa.2021.128113.

[120] H. Zhou , J. Lai , B. Zheng , et al., “From Glutinous‐Rice‐Inspired Adhesive Organohydrogels to Flexible Electronic Devices toward Wearable Sensing, Power Supply, and Energy Storage, ” Advanced Functional Materials 32, no. 1 (2021): 2108423, 10.1002/adfm.202108423.

[121] G. Du , Y. Shao , B. Luo , et al., “Compliant Iontronic Triboelectric Gels with Phase‐Locked Structure Enabled by Competitive Hydrogen Bonding, ” Nano‐Micro Letters 16, no. 1 (2024): 170, 10.1007/s40820-024-01387-4. 38592515 PMC11003937

[122] V. Timosina , T. Cole , H. Lu , et al., “A Non‐Newtonian Liquid Metal Enabled Enhanced Electrography, ” Biosensors and Bioelectronics 235 (2023): 115414, 10.1016/j.bios.2023.115414. 37236012

[123] A. R. Jacob , D. P. Parekh , M. D. Dickey , and L. C. Hsiao , “Interfacial Rheology of Gallium‐Based Liquid Metals, ” Langmuir 35, no. 36 (2019): 11774–11783, 10.1021/acs.langmuir.9b01821. 31407902

[124] B. N. Muller , V. R. Feig , N. S. Colella , G. Traverso , and S. M. Hashmi , “Thiol Coordination Softens Liquid Metal Particles to Improve on‐Demand Conductivity, ” ACS Nano 18, no. 21 (2024): 13768–13780, 10.1021/acsnano.4c01988. 38745441 PMC11140741

[125] Y. Lan , S. Chen , and T. Dong , “Transparent, Stretchable PVC–Liquid Metal Composite Sensor for Smart Wearable Electronics, ” ACS Applied Materials & Interfaces 17, no. 24 (2025): 36047–36058, 10.1021/acsami.5c05797. 40460456

[126] C. Ma , K. Wang , D. Gao , and G. Zhao , “Highly Sensitive and Selective Flexible Anisotropic Strain Sensor Based on Liquid Metal/Conductive Ink for Wearable Applications, ” Composites Part B: Engineering 281 (2024): 111538, 10.1016/j.compositesb.2024.111538.

[127] J. Xu , H. Guo , H. Ding , et al., “Printable and Recyclable Conductive Ink Based on a Liquid Metal with Excellent Surface Wettability for Flexible Electronics, ” ACS Applied Materials & Interfaces 13, no. 6 (2021): 7443–7452, 10.1021/acsami.0c20549. 33528998

[128] Z. Ma , S. Lu , Y. Wu , et al., “Pressure‐Activatable Liquid Metal Composites Flexible Sensor with Antifouling and Drag Reduction Functional Surface, ” ACS Applied Materials & Interfaces 15, no. 47 (2023): 54952–54965, 10.1021/acsami.3c12910. 37966900

[129] X. Kang , L. Sun , Y. Wu , L. Chen , D. Zhang , and J. Yang , “Liquid Metal Elastomer‐Based U‐Shaped Electrode Flexible Strain Sensors with Enhanced Stability and Sensitivity, ” ACS Applied Materials & Interfaces 17, no. 3 (2025): 5527–5537, 10.1021/acsami.4c19546. 39797774

[130] T. Yu , X. Lü , C. Xue , and W. Bao , “Biomimetic Three‐Dimensional Microchannel Non‐Destructive Self‐Healing Flexible Strain Sensor Based on Liquid Metal‐Polydimethylsiloxane Elastomer, ” Chemical Engineering Journal 497 (2024): 154526, 10.1016/j.cej.2024.154526.

[131] J. Luo , Y. Hu , S. Luo , et al., “Strong and Multifunctional Lignin/Liquid Metal Hydrogel Composite as Flexible Strain Sensors, ” ACS Sustainable Chemistry & Engineering 12, no. 18 (2024): 7105–7114, 10.1021/acssuschemeng.4c00940.

[132] X.‐H. Zhang , B. Wang , B. Zhou , et al., “Recent Advances in MXene‐Based Flexible Pressure Sensors for Medical Monitoring, ” Rare Metals 44, no. 6 (2025): 3653–3685, 10.1007/s12598-024-03157-y.

[133] J. Xu , L. Peng , S. Yuan , et al., “Advanced Optical‐Thermal Integrated Flexible Tactile Sensor for High‐Fine Recognition of Liquid Property in Non‐Contact Mode, ” Advanced Functional Materials 34, no. 52 (2024): 2410885, 10.1002/adfm.202410885.

[134] Y. Sun , Y. Yang , D. Yao , et al., “Biomimetic Multilayer Flexible Sensors for Multifunctional Underwater Sensing, ” Chemical Engineering Journal 492 (2024): 152273, 10.1016/j.cej.2024.152273.

[135] M. Jia , C. Yi , Y. Han , et al., “Hierarchical Network Enabled Flexible Textile Pressure Sensor with Ultrabroad Response Range and High‐Temperature Resistance, ” Advanced Science 9, no. 14 (2022): 2105738, 10.1002/advs.202105738. 35289123 PMC9108605

[136] H. Zhou , K. Guo , S. Ma , et al., “A Triple‐Layer Structure Flexible Sensor Based on Nano‐Sintered Silver for Power Electronics with High Temperature Resistance and High Thermal Conductivity, ” Chemical Engineering Journal 432 (2022): 134431, 10.1016/j.cej.2021.134431.

[137] J. S. Benas , F. C. Liang , M. Venkatesan , et al., “Recent Development of Sustainable Self‐Healable Electronic Skin Applications, a Review with Insight, ” Chemical Engineering Journal 466 (2023): 142945, 10.1016/j.cej.2023.142945.

[138] E. Roels , S. Terryn , F. Iida , et al., “Processing of Self‐Healing Polymers for Soft Robotics, ” Advanced Materials 34, no. 1 (2022): 2104798, 10.1002/adma.202104798. 34610181

[139] M. Zhang , L. Zhao , F. Tian , et al., “Bionic Artificial Skin Based on Self‐Healable Ionogel Composites with Tailored Mechanics and Robust Interfaces, ” Advanced Materials 36, no. 35 (2024): 2405776, 10.1002/adma.202405776. 38966888

[140] D. Yang , K. Zhao , R. Yang , et al., “A Rational Design of Bio‐Derived Disulfide CANs for Wearable Capacitive Pressure Sensor, ” Advanced Materials 36, no. 30 (2024): 2403880, 10.1002/adma.202403880. 38723049

[141] C. Zhang , Y. Zhou , H. Han , H. Zheng , W. Xu , and Z. Wang , “Dopamine‐Triggered Hydrogels with High Transparency, Self‐Adhesion, and Thermoresponse as Skinlike Sensors, ” ACS Nano 15, no. 1 (2021): 1785–1794, 10.1021/acsnano.0c09577. 33404217

[142] S. N. Li , Z. R. Yu , B.‐F. Guo , et al., “Environmentally Stable, Mechanically Flexible, Self‐adhesive, and Electrically Conductive Ti_3_C_2_TX MXene Hydrogels for Wide‐temperature Strain Sensing, ” Nano Energy 90 (2021): 106502, 10.1016/j.nanoen.2021.106502.

[143] X. Wang , M. Zhao , L. Zhang , et al., “Liquid Metal Bionic Instant Self‐Healing Flexible Electronics with Full Recyclability and High Reliability, ” Chemical Engineering Journal 431 (2022): 133965, 10.1016/j.cej.2021.133965.

[144] H. Sun , Y. Han , M. Huang , et al., “Highly Stretchable, Environmentally Stable, Self‐Healing and Adhesive Conductive Nanocomposite Organohydrogel for Efficient Multimodal Sensing, ” Chemical Engineering Journal 480 (2024): 148305, 10.1016/j.cej.2023.148305.

[145] C. B. Cooper , S. E. Root , L. Michalek , et al., “Autonomous Alignment and Healing in Multilayer Soft Electronics Using Immiscible Dynamic Polymers, ” Science 380, no. 6648 (2023): 935–941, 10.1126/science.adh0619. 37262169

[146] Y. Shi , Q. Liu , Q. Pan , D. Yang , and T. Wang , “Fabrication of PS‐DVB@Cu Core‐Shell Microsphere for Anisotropic Conductive Adhesives by Electroless Plating with Copper Nanoparticles as Seeds, ” Colloids and Surfaces a: Physicochemical and Engineering Aspects 683 (2024): 133037, 10.1016/j.colsurfa.2023.133037.

[147] H. Ye , J. Chen , S. Wei , et al., “One‐Pot Biosynthesis of Two‐Dimensional Dendrite‐Like Cu/C Hybrids with Excellent Anti‐Oxidation Property for Flexible Electronics, ” Journal of Alloys and Compounds 957 (2023): 170398, 10.1016/j.jallcom.2023.170398.

[148] C. Chen , S. Zhao , T. Sekiguchi , and K. Suganuma , “Large‐scale Bare Cu Bonding by 10 εm‐sized Cu–Ag Composite Paste in Low Temperature Low Pressure Air Conditions, ” Journal of Science: Advanced Materials and Devices 8, no. 3 (2023): 100606, 10.1016/j.jsamd.2023.100606.

[149] W. Li , Q. Cen , W. Li , et al., “A Green Method for Synthesizing Novel Nanoparticles and Their Application in Flexible Conductive Patterns, ” Journal of Materiomics 6, no. 2 (2020): 300–307, 10.1016/j.jmat.2020.02.003.

[150] S. Hong , C. Liu , S. Hao , et al., “Antioxidant High‐Conductivity Copper Paste for Low‐Cost Flexible Printed Electronics, ” npj Flexible Electronics 6, no. 1 (2022): 1–9, 10.1038/s41528-022-00151-1.

[151] W. Ma , Z. Jiang , T. Lu , R. Xiong , and C. Huang , “Lightweight, Elastic and Superhydrophobic Multifunctional Nanofibrous Aerogel for Self‐Cleaning, Oil/Water Separation and Pressure Sensing, ” Chemical Engineering Journal 430 (2022): 132989, 10.1016/j.cej.2021.132989.

[152] G. Shi , T. Zhan , Y. Hu , Z. Guo , S. Wang , and A. Stretchable , “A Stretchable, Self‐adhesive, Conductive Double‐network Hydrogel and Its Application in Flexible Strain Sensors, ” Journal of Polymer Research 30, no. 2 (2023): 61, 10.1007/s10965-023-03441-y.

[153] P. Tan , H. Wang , F. Xiao , et al., “Solution‐Processable, Soft, Self‐Adhesive, and Conductive Polymer Composites for Soft Electronics, ” Nature Communications 13, no. 1 (2022): 358, 10.1038/s41467-022-28027-y. PMC8766561 35042877

[154] X. Zhang , Y. Su , J. Xu , et al., “Self‐Adhesive ILn@MXene Multifunctional Hydrogel with Excellent Dispersibility for Human‐Machine Interaction, Capacitor, Antibacterial and Detecting Various Physiological Electrical Signals in Humans and Animals, ” Nano Energy 133 (2025): 110484, 10.1016/j.nanoen.2024.110484.

[155] X. Huang , C. J. Jiménez , M. Guix , C. M. Xufré , Y. Tuersun , and S. Chu , “Rapidly Polymerized Multifunctional Hydrogel Sensor Initiated by Nanocellulose‐Stabilized MXene‐Coated Liquid Metal for Advanced Wearable Applications, ” Rare Metals 44, no. 9 (2025): 6402–6416, 10.1007/s12598-025-03359-y.

[156] G. Choi , J. Kim , H. Kim , et al., “Motion‐Adaptive Tessellated Skin Patches with Switchable Adhesion for Wearable Electronics, ” Advanced Materials 37, no. 4 (2025): 2412271, 10.1002/adma.202412271. 39428834 PMC11775872

[157] T. Sun , J. Zhang , Y. Chen , et al., “Bioinspired, Ultra‐Sensitive Flexible Strain Sensor Based on Ceramic Fiber Paper with Superhydrophobic and High‐Temperature‐Resistant Properties, ” Advanced Materials Technologies 8, no. 3 (2023): 2200972, 10.1002/admt.202200972.

[158] N. Sakhuja , R. Kumar , P. Katare , and N. Bhat , “Structure‐Driven, Flexible, Multilayered, Paper‐Based Pressure Sensor for Human–Machine Interfacing, ” ACS Sustainable Chemistry & Engineering 10, no. 30 (2022): 9697–9706, 10.1021/acssuschemeng.1c08491.

[159] H. Guan , J. Meng , Z. Cheng , and X. Wang , “Processing Natural Wood into a High‐Performance Flexible Pressure Sensor, ” ACS Applied Materials & Interfaces 12, no. 41 (2020): 46357–46365, 10.1021/acsami.0c12561. 32967417

[160] M. Wang , X. Wang , Z. He , et al., “Stretchable, Washable, and Anti‐Ultraviolet I‐Textile‐Based Wearable Device for Motion Monitoring, ” ACS Applied Materials & Interfaces 16, no. 10 (2024): 13052–13059, 10.1021/acsami.3c18203. 38414333

[161] X. Zhu , T. Cui , F. Su , and B. He , “Robust Polyethylene Sensor Complex for Multi‐Dimensional Monitoring, ” Chemical Engineering Journal 463 (2023): 142407, 10.1016/j.cej.2023.142407.

[162] M. Dirany , L. Dies , F. Restagno , L. Léger , C. Poulard , and G. Miquelard‐Garnier , “Chemical Modification of PDMS Surface without Impacting the Viscoelasticity: Model Systems for a Better Understanding of Elastomer/Elastomer Adhesion and Friction, ” Colloids and Surfaces A: Physicochemical and Engineering Aspects 468 (2015): 174–183, 10.1016/j.colsurfa.2014.12.036.

[163] Y. Zhang , T. Zhang , H. Shi , Q. Liu , and T. Wang , “Fabrication of Flexible Copper Patterns by Electroless Plating with Copper Nanoparticles as Seeds, ” Applied Surface Science 547 (2021): 149220, 10.1016/j.apsusc.2021.149220.

[164] W. Yao , Y. Kang , S. Park , O. L. Li , and Y. Cho , “Development of a Robust, Self‐Cleaning, Amphiphobic, and Electrically Conductive Coating on a Flexible Polymer Substrate, ” Materials & Design 182 (2019): 108023, 10.1016/j.matdes.2019.108023.

[165] B. K. Lee , J. H. Ryu , I.‐B. Baek , et al., “Silicone‐Based Adhesives with Highly Tunable Adhesion Force for Skin‐Contact Applications, ” Advanced Healthcare Materials 6, no. 22 (2017): 1700621, 10.1002/adhm.201700621. 28795496

[166] H. Madadi and J. Casals‐Terré , “Long‐Term Behavior of Nonionic Surfactant‐Added PDMS for Self‐Driven Microchips, ” Microsystem Technologies 19, no. 1 (2013): 143–150, 10.1007/s00542-012-1641-7.

[167] J. Seo and L. P. Lee , “Effects on Wettability by Surfactant Accumulation/Depletion in Bulk Polydimethylsiloxane (PDMS), ” Sensors and Actuators B: Chemical 119, no. 1 (2006): 192–198, 10.1016/j.snb.2005.12.019.

[168] H. Hu , S. Li , C. Ying , et al., “Hydrophilic PDMS with a Sandwich‐Like Structure and no Loss of Mechanical Properties and Optical Transparency, ” Applied Surface Science 503 (2020): 144126, 10.1016/j.apsusc.2019.144126.

[169] C. Duque Londono , S. F. Cones , J. Deng , et al., “Bioadhesive Interface for Marine Sensors on Diverse Soft Fragile Species, ” Nature Communications 15, no. 1 (2024): 2958, 10.1038/s41467-024-46833-4. PMC11021473 38627374

[170] Y. Dong , D. Xu , H. Yu , Q. Mi , F. Zou , and X. Yao , “Highly Sensitive, Scrub‐Resistant, Robust Breathable Wearable Silk Yarn Sensors via Interfacial Multiple Covalent Reactions for Health Management, ” Nano Energy 115 (2023): 108723, 10.1016/j.nanoen.2023.108723.

[171] T. Q. Trung , N. T. Tien , D. Kim , M. Jang , O. J. Yoon , and N. E. Lee , “A Flexible Reduced Graphene Oxide Field‐Effect Transistor for Ultrasensitive Strain Sensing, ” Advanced Functional Materials 24, no. 1 (2014): 117–124, 10.1002/adfm.201301845.

[172] Y. Lei , J. Yang , Y. Xiong , et al., “Surface Engineering AgNW Transparent Conductive Films for Triboelectric Nanogenerator and Self‐Powered Pressure Sensor, ” Chemical Engineering Journal 462 (2023): 142170, 10.1016/j.cej.2023.142170.

[173] I. Byun , A. W. Coleman , and B. Kim , “Transfer of Thin Au Films to Polydimethylsiloxane (PDMS) with Reliable Bonding Using (3‐Mercaptopropyl)Trimethoxysilane (MPTMS) as a Molecular Adhesive, ” Journal of Micromechanics and Microengineering 23, no. 8 (2013): 085016, 10.1088/0960-1317/23/8/085016.

[174] Y. T. Kwon , Y. S. Kim , Y. Lee , et al., “Ultrahigh Conductivity and Superior Interfacial Adhesion of a Nanostructured, Photonic‐Sintered Copper Membrane for Printed Flexible Hybrid Electronics, ” ACS Applied Materials & Interfaces 10, no. 50 (2018): 44071–44079, 10.1021/acsami.8b17164. 30452228

[175] Y. Wang , Y. Wang , J.‐J. Chen , et al., “A Facile Process Combined with Inkjet Printing, Surface Modification and Electroless Deposition to Fabricate Adhesion‐Enhanced Copper Patterns on Flexible Polymer Substrates for Functional Flexible Electronics, ” Electrochimica Acta 218 (2016): 24–31, 10.1016/j.electacta.2016.08.143.

[176] Y. Ding , C. Xue , X. Guo , X. Wang , S. Jia , and Q. An , “Fabrication of TPE/CNTs Film at Air/Water Interface for Flexible and Superhydrophobic Wearable Sensors, ” Chemical Engineering Journal 409 (2021): 128199, 10.1016/j.cej.2020.128199.

[177] Y.‐R. Ding , C. H. Xue , Q. Q. Fan , et al., “Fabrication of Superhydrophobic Conductive Film at Air/Water Interface for Flexible and Wearable Sensors, ” Chemical Engineering Journal 404 (2021): 126489, 10.1016/j.cej.2020.126489.

[178] J. Wang , M. Tenjimbayashi , Y. Tokura , et al., “Bionic Fish‐Scale Surface Structures Fabricated via Air/Water Interface for Flexible and Ultrasensitive Pressure Sensors, ” ACS Applied Materials & Interfaces 10, no. 36 (2018): 30689–30697, 10.1021/acsami.8b08933. 30003780

[179] M. Tang , Z. Jiang , Z. Wang , et al., “High‐Adhesion PDMS/Ag Conductive Composites for Flexible Hybrid Integration, ” Chemical Engineering Journal 451 (2023): 138730, 10.1016/j.cej.2022.138730.

[180] X. Pan , Y. Wang , Z. Xu , et al., “Improving Electronics Stability via Covalent‐Linked Interfaces for Ultra‐Robust Pressure Sensing Array, ” Chemical Engineering Journal 489 (2024): 151354, 10.1016/j.cej.2024.151354.

[181] H. Feng , P. Liu , X. Guo , et al., “PSS Modified by 3‐Aminopropyltrimethoxysilane Linking Large‐Area GNPs/PSS to Silicone Rubber with Stable Interface Combination for High Sensitivity Flexible Resistive Sensor, ” Chemical Engineering Journal 465 (2023): 143009, 10.1016/j.cej.2023.143009.

[182] Y. Cao , H. Zhong , B. Chen , X. Lin , J. Shen , and M. Ye , “Liquid Metal‐Assisted Hydrothermal Preparation of Cobalt Disulfide on the Polymer Tape Surface for Flexible Sensor, ” Nano Research 16, no. 5 (2023): 7575–7582, 10.1007/s12274-022-5357-1.

[183] H. Wang , X. Dou , Z. Wang , et al., “Boosting Sensitivity and Durability of Pressure Sensors Based on Compressible Cu Sponges by Strengthening Adhesion of “Rigid‐Soft” Interfaces, ” Small 19, no. 47 (2023): 2303234, 10.1002/smll.202303234. 37501331

[184] L. Mao , T. Pan , L. Lin , et al., “Simultaneously Enhancing Sensitivity and Operation Range of Flexible Pressure Sensor by Constructing a Magnetic‐Guided Microstructure in Laser‐Induced Graphene Composite, ” Chemical Engineering Journal 481 (2024): 148639, 10.1016/j.cej.2024.148639.

[185] S. Wakabayashi , T. Arie , S. Akita , K. Nakajima , and K. Takei , “A Multitasking Flexible Sensor via Reservoir Computing, ” Advanced Materials 34, no. 26 (2022): 2201663, 10.1002/adma.202201663. 35442552

[186] J. Zhu , Y. Xiao , X. Zhang , et al., “Direct Laser Processing and Functionalizing PI/PDMS Composites for an on‐Demand, Programmable, Recyclable Device Platform, ” Advanced Materials 36, no. 35 (2024): 2400236, 10.1002/adma.202400236. PMC11361840 38563243

[187] K. M. Clark , D. T. Nekoba , K. L. Viernes , J. Zhou , and T. R. Ray , “Fabrication of High‐Resolution, Flexible, Laser‐Induced Graphene Sensors via Stencil Masking, ” Biosensors and Bioelectronics 264 (2024): 116649, 10.1016/j.bios.2024.116649. 39137522 PMC11368413

[188] L. Cheng , C. S. Yeung , L. Huang , et al., “Flash Healing of Laser‐Induced Graphene, ” Nature Communications 15, no. 1 (2024): 2925, 10.1038/s41467-024-47341-1. PMC10995154 38575649

[189] C. Lu , Y. Gao , S. Yu , H. Zhou , X. Wang , and L. Li , “Non‐Fluorinated Flexible Superhydrophobic Surface with Excellent Mechanical Durability and Self‐Cleaning Performance, ” ACS Applied Materials & Interfaces 14, no. 3 (2022): 4750–4758, 10.1021/acsami.1c21840. 35029969

[190] Q. Xiang , S. Luo , Y. Xue , and N. Liao , “Flexible and Sensitive Three‐Dimension Structured Indium Tin Oxide/Zinc Aluminum Oxide/Cu Composites Thin‐Films Pressure Sensors for Healthcare Monitoring, ” Surfaces and Interfaces 52 (2024): 104854, 10.1016/j.surfin.2024.104854.

[191] C. Chen , R. Chen , W. Gu , B. Liu , and H. Ji , “In Situ SEM Observation and Interface Adhesion Strength Analysis of a Serpentine Interconnect on a Flexible Electronic Composite Film, ” Applied Physics Express 14, no. 1 (2021): 017002, 10.35848/1882-0786/abd370.

[192] T. Matsumae , M. Fujino , and T. Suga , “Room‐Temperature Bonding Method for Polymer Substrate of Flexible Electronics by Surface Activation Using Nano‐Adhesion Layers, ” Japanese Journal of Applied Physics 54, no. 10 (2015): 101602, 10.7567/JJAP.54.101602.

[193] N. Bhandaru , N. Agrawal , M. Banik , R. Mukherjee , and A. Sharma , “Hydrophobic Recovery of Cross‐Linked Polydimethylsiloxane Films and Its Consequence in Soft Nano Patterning, ” Bulletin of Materials Science 43, no. 1 (2020): 186, 10.1007/s12034-020-02162-y.

[194] H. T. Kim and O. C. Jeong , “PDMS Surface Modification Using Atmospheric Pressure Plasma, ” Microelectronic Engineering 88, no. 8 (2011): 2281–2285, 10.1016/j.mee.2011.02.084.

[195] X. Chen , Q. Zeng , J. Shao , et al., “Channel‐Crack‐Designed Suspended Sensing Membrane as a Fully Flexible Vibration Sensor with High Sensitivity and Dynamic Range, ” ACS Applied Materials & Interfaces 13, no. 29 (2021): 34637–34647, 10.1021/acsami.1c09963. 34269049

[196] C. Sun , J. Zhang , Y. Zhang , et al., “Design and Fabrication of Flexible Strain Sensor Based on ZnO‐Decorated PVDF via Atomic Layer Deposition, ” Applied Surface Science 562 (2021): 150126, 10.1016/j.apsusc.2021.150126.

[197] J. Liu , Y. Ge , D. Zhang , et al., “Plasma Cleaning and Self‐Limited Welding of Silver Nanowire Films for Flexible Transparent Conductors, ” ACS Applied Nano Materials 4, no. 2 (2021): 1664–1671, 10.1021/acsanm.0c03137.

[198] F.‐T. Zhang , L. Xu , J. H. Chen , et al., “Adhesion‐Enhanced Flexible Conductive Metal Patterns on Polyimide Substrate through Direct Writing Catalysts with Novel Surface‐Modification Electroless Deposition, ” ChemistrySelect 3, no. 26 (2018): 7612–7618, 10.1002/slct.201801081.

[199] H. Liu , M. Takakuwa , M. Yamamoto , et al., “Ultrathin Bending Sensor with Ultrahigh Robustness and Reliability for Robotic Applications, ” npj Flexible Electronics 9, no. 1 (2025): 123, 10.1038/s41528-025-00498-1.

[200] J. Kim , T. Lee , T. Kim , and K. Paik , “The Effect of Anisotropic Conductive Films Adhesion on the Bending Reliability of Chip‐in‐Flex Packages for Wearable Electronics Applications, ” IEEE Transactions on Components, Packaging and Manufacturing Technology 7, no. 10 (2017): 1583–1591, 10.1109/TCPMT.2017.2718186.

[201] S. Erlenbach , K. Mondal , J. Ma , et al., “Flexible‐to‐Stretchable Mechanical and Electrical Interconnects, ” ACS Applied Materials & Interfaces 15, no. 4 (2023): 6005–6012, 10.1021/acsami.2c14260. 36599089

[202] S. Chung , C. Riley , and H. Taylor , “A Bioinspired Triple‐Hierarchical Superhydrophobic Surface with Exceptional Robustness to Water Impingement, ” Advanced Engineering Materials 25, no. 3 (2023): 2201315, 10.1002/adem.202201315.

[203] J. H. Kim , H. E. Jeong , S. M. Kim , and S. M. Kang , “Enhanced Directional Adhesion Behavior of Mushroom‐Shaped Microline Arrays, ” International Journal of Precision Engineering and Manufacturing‐Green Technology 7, no. 1 (2020): 239–245, 10.1007/s40684-019-00112-6.

[204] S. Li , H. Tian , J. Shao , H. Liu , D. Wang , and W. Zhang , “Switchable Adhesion for Nonflat Surfaces Mimicking Geckos″ Adhesive Structures and Toe Muscles, ” ACS Applied Materials & Interfaces 12, no. 35 (2020): 39745–39755, 10.1021/acsami.0c08686. 32666785

[205] D. Wang , H. Hu , S. Li , et al., “Sensing‐Triggered Stiffness‐Tunable Smart Adhesives, ” Science Advances 9, no. 11 (2023): adf4051, 10.1126/sciadv.adf4051. PMC10017039 36921055

[206] S. Park , J. Kim , S. H. Lee , et al., “Water‐Repellent and Self‐Attachable Flexible Conductive Patch, ” Applied Physics Letters 123, no. 7 (2023): 71601, 10.1063/5.0160217.

[207] S. Li , H. Liu , H. Tian , et al., “Dytiscus Lapponicus‐Inspired Structure with High Adhesion in Dry and Underwater Environments, ” ACS Applied Materials & Interfaces 13, no. 35 (2021): 42287–42296, 10.1021/acsami.1c13604. 34455771

[208] M. Seong , C. Jeong , H. Yi , et al., “Adhesion of Bioinspired Nanocomposite Microstructure at High Temperatures, ” Applied Surface Science 413 (2017): 275–283, 10.1016/j.apsusc.2017.04.036.

[209] I. Hwang , M. Seong , H. Yi , et al., “Low‐Resistant Electrical and Robust Mechanical Contacts of Self‐Attachable Flexible Transparent Electrodes with Patternable Circuits, ” Advanced Functional Materials 30, no. 17 (2020): 2000458, 10.1002/adfm.202000458.

[210] Y. Liu , Q. He , Y. Jia , J. Wang , and L. Yin , “A Bioinspired Fish Scale‐Based Flexible Strain Sensor for Human Motion Signal Detection, ” Surfaces and Interfaces 62 (2025): 106256, 10.1016/j.surfin.2025.106256.

[211] M. Cheng , Y. Liu , B. Zhong , et al., “Superhydrophobic Surface with Controllable Adhesion for Anti‐Roof‐Collapse Application in Flexible Microfluidics, ” Advanced Materials Interfaces 6, no. 22 (2019): 1901178, 10.1002/admi.201901178.

[212] L. Ma and N. Soin , “Recent Progress in Printed Physical Sensing Electronics for Wearable Health‐Monitoring Devices: a Review, ” IEEE Sensors Journal 22, no. 5 (2022): 3844–3859, 10.1109/JSEN.2022.3142328.

[213] X. Wang , G. Wu , X. Zhang , et al., “Traditional Chinese Medicine (TCM)‐Inspired Fully Printed Soft Pressure Sensor Array with Self‐Adaptive Pressurization for Highly Reliable Individualized Long‐Term Pulse Diagnostics, ” Advanced Materials 37, no. 1 (2024): 2410312, 10.1002/adma.202410312. 39344553

[214] M. Ma , R. Sun , S. Li , et al., “Fabricating of Double Layered Flexible Pressure Sensor with a High‐Sensitivity Based on Inkjet Printed Micro‐Concave Structure, ” Sensors and Actuators A: Physical 351 (2023): 114161, 10.1016/j.sna.2023.114161.

[215] Y. Jin , S. Xue , and Y. He , “Flexible Pressure Sensors Enhanced by 3D‐Printed Microstructures, ” Advanced Materials 37, no. 27 (2025): 2500076, 10.1002/adma.202500076. 40249136

[216] Q. Wang , Q. Li , J. Zou , et al., “A Multi‐Metric Evaluation Framework Guides the High‐Throughput Design of Versatile Flexible Pressure Sensors, ” Composites Part B: Engineering 312 (2026): 113327, 10.1016/j.compositesb.2025.113327.

[217] Z. Hanif , M. Z. Tariq , D. Choi , M. La , and S. J. Park , “Solution‐Processed Deposition Based on Plant Polyphenol for Silver Conductive Coating and Its Application on Human Motions Detecting Sensor, ” Composites Science and Technology 201 (2021): 108550, 10.1016/j.compscitech.2020.108550.

[218] S. Khan , L. Lorenzelli , and R. S. Dahiya , “Screen Printed Flexible Pressure Sensors Skin, ” in 2014 25th Annual SEMI Advanced Semiconductor Manufacturing Conference (ASMC), (2014): 219–224, 10.1109/ASMC.2014.6847002.

[219] M. Li , S. Chen , B. Fan , B. Wu , and X. Guo , “Printed Flexible Strain Sensor Array for Bendable Interactive Surface, ” Advanced Functional Materials 30, no. 34 (2020): 2003214, 10.1002/adfm.202003214.

[220] Y. Zhang , B. Zhang , Y. Lv , P. Wang , T. Liu , and C. Meng , “Flexible and Breathable MXene‐Modified Paper‐Based Piezoresistive Pressure Sensors Integrated into Airbag Pillow for Sleep Monitoring, ” Soft Science 5, no. 2 (2025): 17, 10.20517/ss.2024.68.

[221] T. Yang , Y. Z. Yu , L. S. Zhu , X. Wu , X. H. Wang , and J. Zhang , “Fabrication of Silver Interdigitated Electrodes on Polyimide Films via Surface Modification and Ion‐Exchange Technique and Its Flexible Humidity Sensor Application, ” Sensors and Actuators B: Chemical 208 (2015): 327–333, 10.1016/j.snb.2014.11.043.

[222] T. H. Kang , H. Chang , D. Choi , et al., “Hydrogel‐Templated Transfer‐Printing of Conductive Nanonetworks for Wearable Sensors on Topographic Flexible Substrates, ” Nano Letters 19, no. 6 (2019): 3684–3691, 10.1021/acs.nanolett.9b00764. 31117752

[223] M. E. Imanian , M. Kardan‐Halvaei , F. Nasrollahi , A. Imanian , H. Montazerian , and V. Nasrollahi , “3D Printed Flexible Wearable Sensors Based on Triply Periodic Minimal Surface Structures for Biomonitoring Applications, ” Smart Materials and Structures 32, no. 1 (2023): 015015, 10.1088/1361-665X/aca6bc.

[224] B. Zhou , Y. Li , D. Zhang , et al., “Interfacial Adhesion Enhanced Flexible Polycarbonate/Carbon Nanotubes Transparent Conductive Film for Vapor Sensing, ” Composites Communications 15 (2019): 80–86, 10.1016/j.coco.2019.06.012.

[225] F. Fan , L. Chen , Y. Wang , et al., “Damage‐Free Dry Transfer Printing of Ultrathin Films with on‐Demand Interfacial Adhesion: Principles and Applications, ” Soft Science 5, no. 4 (2025): 52, 10.20517/ss.2025.52.

[226] O. Bezsmertna , R. Xu , E. S. Oliveros Mata , et al., “Versatile Green Transfer of Magnetoelectronics with Loss‐Free Performance and High Adhesion for Interactive Electronics, ” Advanced Functional Materials 35, no. 36 (2025): 2502947, 10.1002/adfm.202502947.

[227] X. Liu , J. Liu , J. Wang , et al., “Bioinspired, Microstructured Silk Fibroin Adhesives for Flexible Skin Sensors, ” ACS Applied Materials & Interfaces 12, no. 5 (2020): 5601–5609, 10.1021/acsami.9b21197. 31927972

[228] K. Huang , X. Cai , R. Shang , et al., “Printed High‐Adhesion Flexible Electrodes Based on an Interlocking Structure for Self‐Powered Intelligent Movement Monitoring, ” ACS Applied Materials & Interfaces 15, no. 50 (2023): 58583–58592, 10.1021/acsami.3c13467. 38079512

[229] S. J. Park , T. Ko , J. Yoon , M. Moon , K. H. Oh , and J. H. Han , “Highly Adhesive and High Fatigue‐Resistant Copper/PET Flexible Electronic Substrates, ” Applied Surface Science 427 (2018): 1–9, 10.1016/j.apsusc.2017.08.195.

[230] S. Pan , F. Zhang , P. Cai , et al., “Mechanically Interlocked Hydrogel–Elastomer Hybrids for on‐Skin Electronics, ” Advanced Functional Materials 30, no. 29 (2020): 1909540, 10.1002/adfm.201909540.

[231] L. Tang , H. Wang , J. Ren , and X. Jiang , “Highly Robust Soft‐Rigid Connections via Mechanical Interlocking for Assembling Ultra‐Stretchable Displays, ” npj Flexible Electronics 8, no. 1 (2024): 50, 10.1038/s41528-024-00337-9.

[232] C. Yan , X. Li , X. Wang , et al., “Extremely Stable, Multidirectional, All‐in‐One Piezoelectric Bending Sensor with Cycle up to Million Level, ” Advanced Functional Materials 34, no. 49 (2024): 2409093, 10.1002/adfm.202409093.

[233] O. Gul , M. Song , C. Y. Gu , et al., “Bioinspired Omnidirectional Interface Engineered Flexible Island for Highly Stretchable Electronics, ” Small 21, no. 32 (2025): 2410247, 10.1002/smll.202410247. 39891029

[234] M. Zhu , S. Ji , Y. Luo , et al., “A Mechanically Interlocking Strategy Based on Conductive Microbridges for Stretchable Electronics, ” Advanced Materials 34, no. 7 (2022): 2101339, 10.1002/adma.202101339. 34978104

[235] L. Luo , Z. Wu , Q. Ding , et al., “In Situ Structural Densification of Hydrogel Network and Its Interface with Electrodes for High‐Performance Multimodal Artificial Skin, ” ACS Nano 18, no. 24 (2024): 15754–15768, 10.1021/acsnano.4c02359. 38830235

[236] Y. Zhang , J. Yang , X. Hou , et al., “Highly Stable Flexible Pressure Sensors with a Quasi‐Homogeneous Composition and Interlinked Interfaces, ” Nature Communications 13, no. 1 (2022): 1317, 10.1038/s41467-022-29093-y. PMC8913661 35273183

[237] Y. Zhang , X. Zhou , N. Zhang , et al., “Ultrafast Piezocapacitive Soft Pressure Sensors with over 10 kHz Bandwidth via Bonded Microstructured Interfaces, ” Nature Communications 15, no. 1 (2024): 3048, 10.1038/s41467-024-47408-z. PMC11001880 38589497

[238] Y. Gong , L. Yu , X. Lyu , et al., “A Mechanically Robust, Self‐Healing, and Adhesive Biomimetic Camouflage Ionic Conductor for Aquatic Environments, ” Advanced Functional Materials 33, no. 47 (2023): 2305314, 10.1002/adfm.202305314.

[239] S. Ji , C. Wan , T. Wang , et al., “Water‐Resistant Conformal Hybrid Electrodes for Aquatic Endurable Electrocardiographic Monitoring, ” Advanced Materials 32, no. 26 (2020): 2001496, 10.1002/adma.202001496. 32449249

[240] H. Kim , Y. Lee , S. Jung , and H. Lee , “Micropatterned Hydrogel‐Elastomer Hybrids for Flexible Wet‐Style Superhydrophobic Antifogging Tapes, ” Advanced Functional Materials 34, no. 34 (2024): 2401869, 10.1002/adfm.202401869.

[241] T. Liu , M. Liu , S. Dou , et al., “Triboelectric‐Nanogenerator‐Based Soft Energy‐Harvesting Skin Enabled by Toughly Bonded Elastomer/Hydrogel Hybrids, ” ACS Nano 12, no. 3 (2018): 2818–2826, 10.1021/acsnano.8b00108. 29494127

[242] M. H. Schneider , Y. Tran , and P. Tabeling , “Benzophenone Absorption and Diffusion in Poly(Dimethylsiloxane) and Its Role in Graft Photo‐Polymerization for Surface Modification, ” Langmuir 27, no. 3 (2011): 1232–1240, 10.1021/la103345k. 21207954

[243] H. Nakano , S. Kakinoki , and Y. Iwasaki , “Long‐Lasting Hydrophilic Surface Generated on Poly(Dimethyl Siloxane) with Photoreactive Zwitterionic Polymers, ” Colloids and Surfaces B: Biointerfaces 205 (2021): 111900, 10.1016/j.colsurfb.2021.111900. 34102530

[244] S. H. Han , S. I. Kim , H. R. Lee , et al., “Hydrogel‐Based Iontronics on a Polydimethylsiloxane Microchip, ” ACS Applied Materials & Interfaces 13, no. 5 (2021): 6606–6614, 10.1021/acsami.0c19892. 33496567

[245] M. Farman , R. P. Surendra , A. K. Upadhyay , P. Kumar , and E. Thouti , “All‐Polydimethylsiloxane‐Based Highly Flexible and Stable Capacitive Pressure Sensors with Engineered Interfaces for Conformable Electronic Skin, ” ACS Applied Materials & Interfaces 15, no. 28 (2023): 34195–34205, 10.1021/acsami.3c04227. 37415563

[246] J. Li , G. Zhang , Z. Cui , et al., “High Performance and Multifunction Moisture‐Driven Yin–Yang‐Interface Actuators Derived from Polyacrylamide Hydrogel, ” Small 19, no. 38 (2023): 2303228, 10.1002/smll.202303228. 37194983

[247] Y. Jiang , S. Ji , J. Sun , et al., “A Universal Interface for Plug‐and‐Play Assembly of Stretchable Devices, ” Nature 614, no. 7948 (2023): 456–462, 10.1038/s41586-022-05579-z. 36792740

[248] J. Cao , X. Liu , J. Qiu , et al., “Anti‐Friction Gold‐Based Stretchable Electronics Enabled by Interfacial Diffusion‐Induced Cohesion, ” Nature Communications 15, no. 1 (2024): 1116, 10.1038/s41467-024-45393-x. PMC10847152 38321072

[249] X. Liu , Q. Wang , S. Zhou , et al., “Stiffness and Interface Engineered Soft Electronics with Large‐Scale Robust Deformability, ” Advanced Materials 36, no. 40 (2024): 2407886, 10.1002/adma.202407886. 39180261

[250] M. Takakuwa , D. Inoue , L. Sun , et al., “Robust Full‐Surface Bonding of Substrate and Electrode for Ultra‐Flexible Sensor Integration, ” Advanced Materials 37, no. 49 (2025): 2417590, 10.1002/adma.202417590. 39967358 PMC12691895

[251] H. Zhang , Y. Shao , R. Xia , G. Chen , X. Xiang , and Y. Yu , “Stretchable Electrodes with Interfacial Percolation Network, ” Advanced Materials 36, no. 26 (2024): 2401550, 10.1002/adma.202401550. 38591837

[252] J. He , J. Huang , R. Li , et al., “Hysteresis‐Free and Dynamically Resilient Strain Sensor Enabled by Interfacial Coordination, ” Science Advances 12, no. 1 (2026): aea2450, 10.1126/sciadv.aea2450. PMC12757069 41477844

[253] Z. Li , P. Zhang , Y. Shao , Z. Guo , and X. Pu , “Stretchable Iontronics with Robust Interface Bonding between Dielectric and Ion‐Conducting Elastomers, ” Nano Research 16, no. 9 (2023): 11862–11870, 10.1007/s12274-023-5612-3.

[254] Q. Wang , P. Guo , Q. Li , J. Liang , and W. Wu , “Interfacial Adhesive Adaptation Strategies for Flexible Multilayer Pressure Sensors in Sleep Monitoring, ” ACS Sensors 10, no. 4 (2025): 2667–2677, 10.1021/acssensors.4c03273. 39977611

[255] R. Zhou , Y. Jin , W. Zeng , et al., “Janus Hydrophobic Structural Gel with Asymmetric Adhesion in Air/Underwater for Reliable Mechanosensing, ” Advanced Functional Materials 34, no. 33 (2024): 2316687, 10.1002/adfm.202316687.

[256] M. Huang , S. Liu , Y. Chi , et al., “Multimodal Sensing Conductive Organohydrogel Electronics Based on Chitosan‐Encapsulated Mxene Nanocomposites for Deep Learning‐Enhanced Ball Sports Recognition, ” Soft Science 5, no. 2 (2025): 24, 10.20517/ss.2025.07.

[257] H. He , T. Yang , T. Liu , et al., “Soft‐Hard Janus Nanoparticles Triggered Hierarchical Conductors with Large Stretchability, High Sensitivity, and Superior Mechanical Properties, ” Advanced Materials 36, no. 15 (2024): 2312278, 10.1002/adma.202312278. 38266185

